# Diagnostikalgorithmus für das Vorgehen bei Patient:innen über 65 Jahren mit leichtem Schädelhirntrauma in der Notaufnahme

**DOI:** 10.1007/s00113-026-01737-4

**Published:** 2026-08-03

**Authors:** Anne Neubert, Astrid Göldner, Stefanie Williams, Michael Bernhard, Carina Jaekel, Denise Schulz, Joachim Windolf, Dan Bieler

**Affiliations:** 1 Klinik für Orthopädie und Unfallchirurgie, Medizinische Fakultät und Universitätsklinikum Düsseldorf, Heinrich-Heine-Universität Düsseldorf, Düsseldorf, Deutschland; 2 TraumaEvidence @ Deutsche Gesellschaft für Unfallchirurgie, Berlin, Deutschland; 3 Klinik für Gynäkologie, Helios Krankenhaus Krefeld, Krefeld, Deutschland; 4 Stowers Institute for Medical Research, Kansas City, MO USA; 5 Zentrale Notaufnahme, Medizinische Fakultät und Universitätsklinikum Düsseldorf, Heinrich-Heine-Universität Düsseldorf, Düsseldorf, Deutschland; 6 Universitätsklinikum RTWH Aachen, Aachen, Deutschland; 7 Klinik für Unfallchirurgie und Orthopädie, Hand‑, Wiederherstellungs- und Plastische Chirurgie, Bundeswehrzentralkrankenhaus Koblenz, Koblenz, Deutschland

**Keywords:** Geriatrisches Trauma, Computertomographie des Schädels, Notaufnahme, Diagnostikalgorithmus, Evidence-based medicine, Geriatric trauma, Cranial computer tomography, Emergency department, Diagnostic algorithm, Evidence-based medicine

## Abstract

**Hintergrund:**

Leichte Schädelhirntraumata (SHT) nach Niedrigrasanztraumata, wie ebenerdigen Stürzen, betreffen häufig ältere Patienten in der Notaufnahme. Aufgrund des demografischen Wandels ist mit der Zunahme dieser Traumafolge in Notaufnahmen zu rechnen. Ziel der vorliegenden Studie war die Entwicklung eines evidenzbasierten Diagnostikalgorithmus für Patienten ≥ 65 Jahre mit leichtem SHT, die sich in der Notaufnahme vorstellen.

**Methoden:**

Für drei diagnostische Fragestellungen wurden *Systematic Reviews* mit Literaturrecherchen in MEDLINE (Englisch/Deutsch) und einer Handsuche der Quellenangaben durchgeführt. Studien wurden mit dem *Methodological Index for Non-randomized Studies* bewertet. Die Datensynthese erfolgte mittels *vote counting based on direction of effect* und der Bewertung klinischer Einflussfaktoren.

**Ergebnisse:**

Aus der Datenbanksuche wurden 52 Studien mit 142.037 Patienten eingeschlossen. Für die initiale kraniale Bildgebung mittels cCT könnte ein Vorgehen anhand von sechs klinischen Einflussfaktoren eine Alternative zur routinemäßigen Bildgebung darstellen. Bei asymptomatischen, neurologisch unauffälligen Patienten ist eine neurologische Überwachung ohne standardisierte Kontroll-cCT denkbar; zur optimalen Gestaltung liegen jedoch keine belastbaren Daten vor. Die Modalitäten einer neurologischen Überwachung müssen weiter untersucht werden. Die Rolle von Antikoagulation und Thrombozytenaggregationshemmung bleibt unklar.

**Schlussfolgerung:**

Die Ergebnisse bilden eine Grundlage für einen Algorithmus zur Diagnostik des leichten SHT bei älteren Patienten. Die evidenzbasierte Ausgestaltung der neurologischen Überwachung sowie der Einfluss von Gerinnungsmedikation erfordern weitere Forschung.

**Graphic abstract:**

**Figure:**
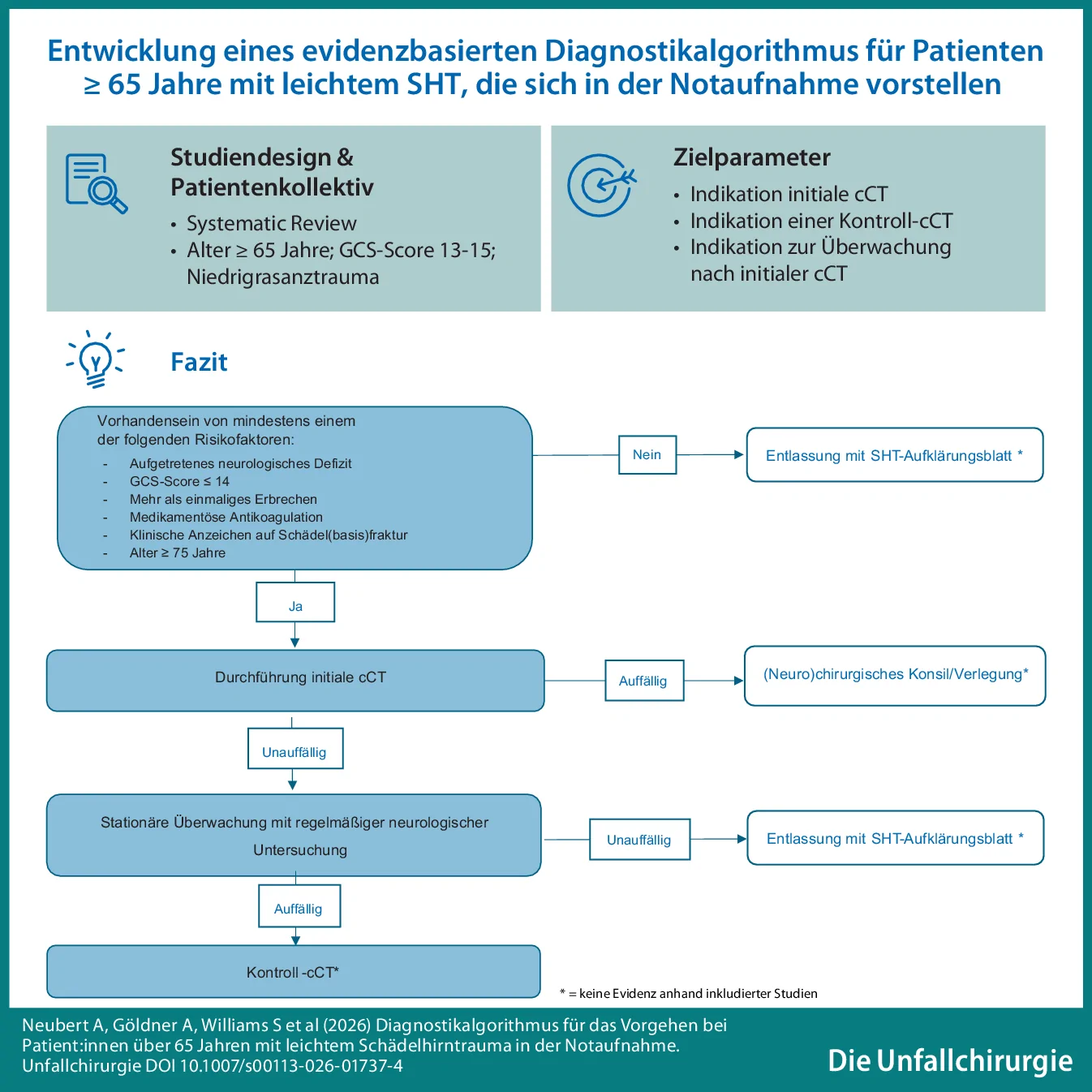

**Zusatzmaterial online:**

Die Online-Version dieses Beitrags (https://doi.org/10.1007/s00113-026-01737-4) enthält weitere Daten zum Beitrag.

## Abstract (en)

**Background:**

Mild traumatic brain injury (TBI) following low-impact trauma, such as falls from ground level, frequently affects older patients in the emergency department. Due to the demographic change, an increase in this type of injury in emergency departments is to be expected. The aim of this study was to develop an evidence-based diagnostic algorithm for patients ≥ 65 years of age with mild TBI who present to the emergency department.

**Methods:**

Systematic reviews with literature searches in MEDLINE (English/German) and manual searches of references were conducted for three diagnostic questions. Studies were evaluated using the * Methodological index for nonrandomized studies*. Data synthesis was performed using *vote counting based on direction of effect* and the evaluation of clinical influencing factors.

**Results:**

A database search yielded 52 studies involving 142,037 patients. For initial cranial imaging using cranial computed tomography (cCT), an approach based on six clinical influencing factors could be an alternative to routine imaging. In asymptomatic patients with no neurological abnormalities, neurological monitoring without standardized control cCT is conceivable; however, there are no reliable data available on the optimal design. The modalities of neurological monitoring need to be further investigated. The role of anticoagulation and antiplatelet treatment remains unclear.

**Conclusion:**

The results form the basis for an algorithm for the diagnosis of mild TBI in older patients. The evidence-based design of neurological monitoring and the influence of anticoagulant medication require further research.

## Einleitung

Aus einem Niedrigrasanztrauma, wie einem ebenerdigen Sturz, resultiert häufig ein leichtes Schädelhirntrauma (SHT). Das leichte SHT ist definiert mit einem Punktwert von 13 bis 15 auf der *Glasgow Coma Scale *(GCS) und kann mit Einschränkungen des Bewusstseins und/oder des Kurzzeitgedächtnisses einhergehen [1]. Neben akuten Symptomen wie Kopfschmerzen kann es auch zu schwerwiegenden Komplikationen, allen voran intrakraniellen Blutungen, kommen.

In Deutschland erleiden jährlich ca. 332/100.000 Einwohner ein SHT, davon fallen ungefähr 90 % auf leichte SHT [2, 3]. Während früher vor allem Verkehrsunfälle ursächlich waren, werden in aktuelleren Studien vermehrt Stürze als Hauptunfallmechanismus genannt, insbesondere bei geriatrischen Patienten [4, 5]. Diese Patienten ≥ 65 Jahren scheinen aufgrund von Vorerkrankungen und medikamentöser Antikoagulation besonders gefährdet für Komplikationen wie Verletzungen der Halswirbelsäule (HWS) und intrakranielle Blutungen zu sein [6–8]. Der demografische Wandel legt eine Zunahme der Fallzahlen in Zukunft nahe, bei gleichzeitig bereits jetzt überlasteten Notaufnahmen in Deutschland [9–12]. Idealerweise berücksichtigt das diagnostische Vorgehen die individuellen Risikofaktoren der Patienten und verhindert so Überdiagnostik bei gleichzeitiger Rücksichtnahme auf personelle und finanzielle Ressourcen in der Notaufnahme.

Ziel der vorliegenden Studie war daher die Entwicklung eines Algorithmus zur Diagnostik von Patienten ≥ 65 Jahren mit leichtem SHT nach Niedrigrasanztrauma in der Notaufnahme. Es wurden die folgenden Forschungsfragen untersucht: Welche Patienten ≥ 65 Jahre mit leichtem SHT sollten 1.) eine initiale CT des Kopfes (cCT) erhalten, 2.) eine Kontroll-cCT erhalten, und zu welchem Zeitpunkt, und 3.) stationär überwacht werden und wie lange?

## Methodik

In dieser Studie wird entsprechend der Weltgesundheitsorganisation (WHO) das leichte SHT als die Folge traumatischer Krafteinwirkung auf den Kopf, bei Patienten mit einem GCS-Punktwert von 13 bis 15 bei Aufnahme, gegebenenfalls mit Einschränkungen des Bewusstseins und/oder Kurzzeitgedächtnisses, verstanden [13].

Zur Beantwortung der Forschungsfragen wurden *Systematic Reviews* durchgeführt. Die systematischen Literatursuchen erfolgten in MEDLINE mittels PubMed (Zusatzmaterial online: Supplement 1 – Suchstrategie). Publikationen auf Deutsch und Englisch wurden berücksichtigt. Literaturreviews, Fallberichte, Kommentare und Leserbriefe und Populationen < 18 Jahre wurden ausgeschlossen. *Systematic Reviews* wurden ebenfalls ausgeschlossen, um eine doppelte Wertung von Studienergebnissen zu vermeiden. Tab. 1 fasst Forschungsfragen sowie Ein- und Ausschlusskriterien der Suchen zusammen.

**Tab. 1:** Forschungsfragen mit Ein- und Ausschlusskriterien

Forschungsfrage	Ein- und Ausschlusskriterien
F1: Welche Patienten ≥ 65 Jahren mit leichtem SHT sollten eine initiale cCT erhalten?	*Einschluss:* Publikation untersucht mindestens einen der folgenden Punkte: – Indikation initiale cCT – Indikation einer Kontroll-cCT – Indikation zur Überwachung nach initialer cCT Population: GCS-Punktwert 13–15 (oder separate Daten zu dieser Gruppe); durchschnittliches Alter ≥ 65 Jahre (oder separate Daten zu dieser Gruppe); möglichst mehrheitlicher Unfallmechanismus Niedrigrasanztrauma *Ausschluss:* Population mit einem durchschnittlichen Alter unter 65 Jahren; > 5 % GCS-Punktwert ≤ 12; > 5 % der eingeschlossenen Patienten erlitten ein Polytrauma (definiert als ISS ≥ 16)
F2: Sollten Patienten ≥ 65 Jahren mit leichtem SHT eine Kontroll-cCT erhalten und, wenn ja, zu welchem Zeitpunkt?	
F3: Sollten Patienten ≥ 65 Jahren mit leichtem SHT stationär überwacht werden und, wenn ja, wie lange?	

*SHT* Schädelhirntrauma, *cCT* kraniale Computertomographie, *GCS* Glasgow Coma Scale, *ISS* Injury Severity Score

Publikationen wurden unabhängig voneinander von zwei Autorinnen (AN & AG) in einem zweistufigen Prozess (1. Titel/Abstrakt, 2. Volltext) selektiert. Bei Konflikten wurde eine dritte Autorin (CJ) hinzugezogen. AG führte anschließend eine Handsuche der Quellenverzeichnisse der eingeschlossenen Publikationen sowie potenziell einschlusswürdiger *Systematic Reviews* durch. Die Studien wurden im Anschluss einer oder mehreren Forschungsfrage(n) zugeordnet.

Die Datenextraktion der Studien erfolgte durch AG anhand von an die Forschungsfragen angepassten Evidenztabellen (Zusatzmaterial online: Supplement 2 – Evidenztabellen), eine weitere Autorin (SW) kontrollierte auf Vollständigkeit und Korrektheit. Bei Unklarheiten wurden die korrespondierenden Autoren der Primärstudien kontaktiert.

Die Bewertung der Berichterstattung erfolgte mithilfe des *Methodological Index for Non-Randomized Studies (MINORS)* [14]. AG und SW bestimmten unabhängig voneinander die Punktwerte. Konflikte wurden in Diskussionen gelöst. Im MINORS können maximal 16 (24) Punkte (bei Studien mit Vergleichsgruppe) erreicht werden. Zur Vergleichbarkeit der Berichterstattung der einzelnen Studien erfolgte folgende Einteilung:

- < 8 bzw. < 14 von 16 bzw. 24 möglichen Punkten: insuffiziente Berichterstattung
- 9 bis 12 bzw. 14 bis 20 von 16 bzw. 24 möglichen Punkten: moderate Berichterstattung
- ≥ 13 bzw. ≥ 20 von 16 bzw. 24 möglichen Punkten: suffiziente Berichterstattung

Statistische Medianwerte wurden, wo möglich, nach der *Quantile-estimation*(QE)-Methode in den Mittelwert umgerechnet [16]. Zur Analyse klinischer Einflussfaktoren wurde ein modifiziertes Bewertungsschema nach Ariëns et al. (2000) [17] und Clay et al. (2010) [18] angewandt (Zusatzmaterial online: Supplement 3 – Bewertungsschema Evidenz). Hierbei wurden alle Einflussfaktoren berücksichtigt, die in der Primärstudie in einer multivariaten Regressionsanalyse statistisch signifikant mit dem jeweiligen Endpunkt assoziiert waren (Zusatzmaterial online: Supplement 4 – Regressionsanalysen Primärstudien). In den Algorithmus flossen nur Einflussfaktoren mit Evidenzstufe 1 (starke Evidenz) oder Evidenzstufe 2 (moderate Evidenz) ein. Basierend auf der ungenauen und teilweise fehlenden Berichterstattung vieler der eingeschlossenen Studien, die darüber hinaus häufig retrospektiv waren, wurde von einer Metaanalyse abgesehen. Es wurde eine qualitative Analyse durchgeführt. Die Datenanalyse und -synthese erfolgten somit mithilfe der Methode des *vote counting based on the direction of effect* (VC) [19]. Die Empfehlung einer Maßnahme wurde nicht anhand von *p*-Werten oder statistischer Signifikanz, sondern anhand der Richtung des beobachteten Effekts festgemacht.

## Ergebnisse

### Literatursuche

**Abb. 1:**
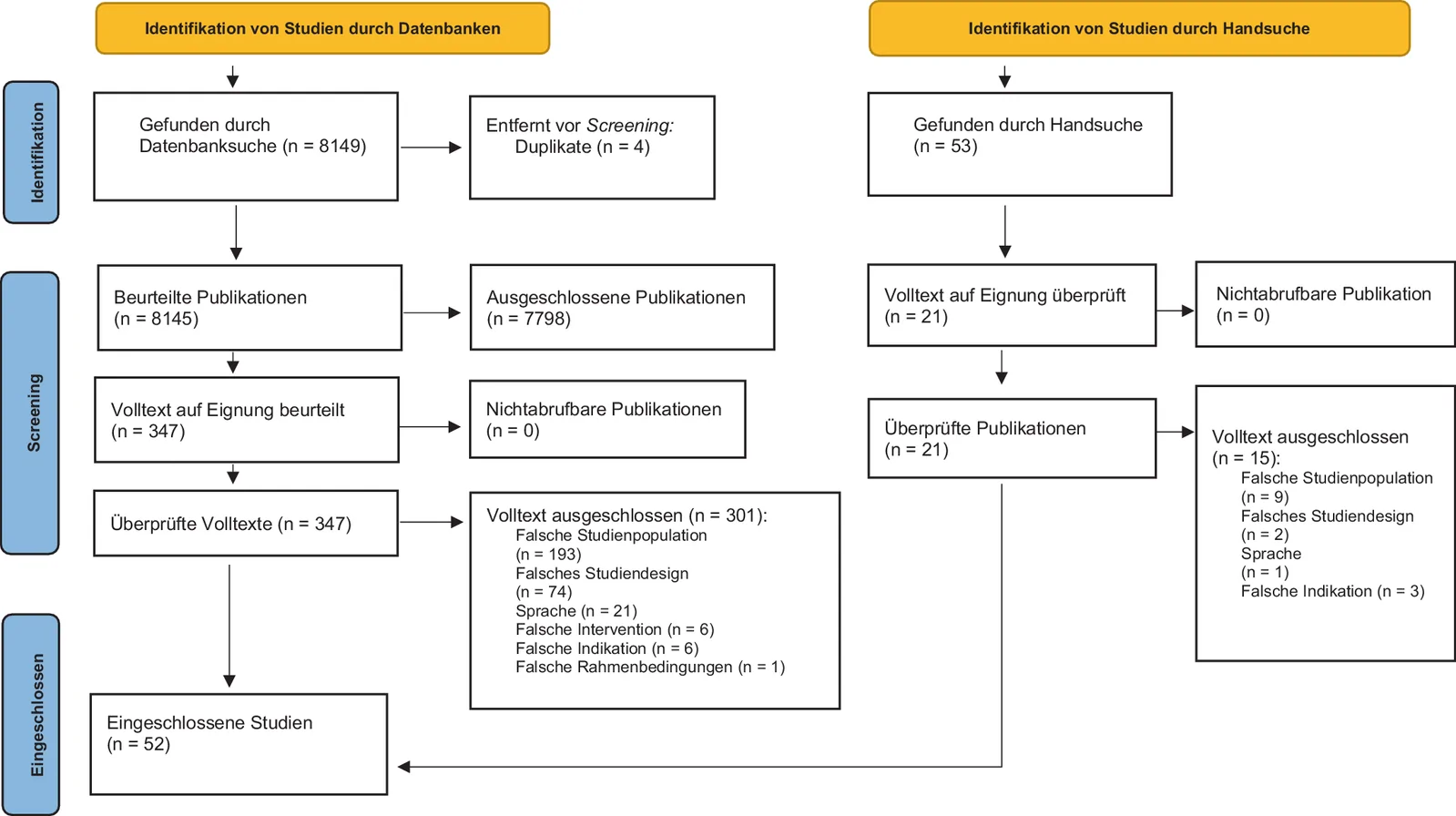
Flussdiagramm – Literatursuche – basierend auf PRISMA-Richtlinien von Page et al. [20]

Zur Beantwortung der Forschungsfragen F1–F3 wurde eine Literatursuche durchgeführt mit insgesamt 8149 Treffern. Nach dem zweistufigem Selektionsprozess und Handsuche wurden 52 Studien mit insgesamt 142.037 Patienten eingeschlossen (Abb. 1: PRISMA-Flussdiagramm). Vier Studien konnten zu keiner Forschungsfrage gruppiert werden, weshalb die Daten nicht in den Algorithmus eingeflossen sind (Zusatzmaterial online: Supplement 7 – sonstige Publikationen).

### F1: Indikation zur initialen cCT

Zu F1 wurden 30 Studien mit insgesamt 38.183 Patienten gruppiert. Demografische Daten und die MINORS-Gesamtpunktzahlen sind Tab. 2 zu entnehmen. Vier Studien wiesen eine moderate [21–24], die verbleibenden 26 Studien eine suffiziente Berichterstattung auf [15, 25–49] (Zusatzmaterial online: Supplement 5 – MINORS).

**Tab. 2:** F1 Studienpopulationen + MINORS-Gesamtpunktzahl

Referenz	Land	Studiendesign	Anzahl der Patienten	Durchschnittliches Alter (Jahre)	Anteil männlicher Patienten (%)	GCS-Verteilung	Unfallmechanismus (%)	Niedrigrasanztrauma (%)	MINORS-Gesamtpunktzahl
Alrahji 2015 [25]	Canada	R‑M-KS	176	79	39,8	15	83	–	13/16
Brewer 2011 [22]	USA	R‑U-QS	141	79	47,5	15	100	91,5	12/16
Brown 2011 [23]	UK	P‑U-FS	66	74,8	59	–	–	–	12/16
Cipriano 2018 [26]	Italien	P‑U-KS	206	81,5	40,8	15	99,0	91,3	22/24
						14	1,0		
Cipriano 2021 [15]	Italien	P‑U-KS	473	81,8	43,6	–	–	77,2	24/24
Colas 2021 [27]	Frankreich	P‑U-QS	840	82,1	37,9	15	92,6	93,2	16/16
						14	4,9		
						13	2,5		
Fabbri 2010 [21]	Italien	P‑U-KS	14.228	–	–	–	–	–	12/16
Fournier 2019 [28]	Canada	R‑U-KS	104	76,9	57,7	–	–	–	15/16
Galliazzo 2019 [49]	Italien	R‑U-KS	1846	65,7	50,1	15	98,1	–	20/24
						14	1,6		
						13	0,3		
Hamden 2014 [29]	USA	P‑U-KS	799	84	33,2	–	–	100	22/24
Ibañez 2018 [30]	Spanien	P‑U-KS	504	79,4	35,5	15	83,5	87,3	14/16
						14	16,3		
Lee 2009 [21]	Korea	R‑U-KS	898	–	58,8	15	81,3	–	14/16
						14	11,9		
						13	6,8		
Leitner 2021 [32]	Österreich	R‑U-KS	1788	58	55,7	–	–	–	20/24
Mack 2003 [33]	USA	R‑U-KS	133	80,4	33,8	15	85	75	20/24
						14	12		
						13	3		
Mason 2017 [34]	UK	R‑M-KS	3534	74,6	49,2	15	81,2	91,6	14/16
						14	7,8		
						13	0,7		
						< 13	1,7		
						Nicht berichtet	8,6		
McCammack 2015 [35]	USA	R‑U-KS	144	74	46,5	–	–	86,1	22/24
Moore 2012 [47]	USA	R‑U-QS	101	–	–	–	–	–	20/24
Mori 2021 [36]	Japan	R‑U-FKS	1118	–	49,3	15	70,3	69,7	21/24
						14	27,9		
						13	1,9		
Nishijima 2012 [48]	USA	P‑M-KS	1064	75,4	47,1	15	87,6	83,3	21/24
						13–15	97,3		
						9–12	1,7		
						8–3	1,0		
Nishijima 2013 [37]	USA	P‑M-QS	982	75,4	47,3	–	*–*	–	13/16
O’Brien 2020 [24]	Canada	R‑M-KS	311	80,1	35,7	–	–	74	12/16
Pages 2020 [38]	Frankreich	R‑U-KS	500	85	40	–	*–*	–	20/24
Reddy 2014 [39]	USA	R‑U-KS	562	83,4	46,8	–	–	100	19/24
Riccardi 2013 [40]	Italien	R‑U-KS	2149	81	44,6	15	100	–	20/24
Sauter 2016 [41]	Schweiz	R‑U-QS	260	78,8	–	–	*–*	–	20/24
Savioli 2020 [42]	Italien	R‑U-KS	2851	64	47	–	–	–	20/24
Teeratakulpisarn 2021 [43]	Thailand	R‑U-KS	100	–	–	–	–	–	20/24
Turcato 2019 [44]	Italien	R‑U-FS	451	83	47,1	–	–	93,0	20/24
Turcato 2021 [45]	Italien	R‑M-KS	1146	–	45,7	–	–	70,0	20/24
Yuksen 2018 [46]	Thailand	R‑U-KS	708	–	54,8	15	81,3	–	20/24
						14	6		
						13	12,7		

*GCS* Glasgow Coma Scale, – nicht berichtet; Code Studiendesign: *R* retrospektiv, *P* prospektiv, *U* unizentrisch, *M* multizentrisch, *KS* Kohortenstudie, *FS* Fallserie, *QS* Querschnittstudie, *FKS* Fall-Kontroll-Studie, *MINORS* Methodological Index for Non-Randomized Studies

Mit Ausnahme von einer Studie [24] untersuchten alle zu F1 eingeschlossenen Studien klinische Einflussfaktoren für eine auffällige initiale cCT. Entsprechend dem Bewertungsschema der Evidenz klinischer Einflussfaktoren sprachen sechs Risikofaktoren für das Vorliegen einer pathologischen cCT der Evidenzstufe 1 (starke Evidenz):

- „Aufgetretenes neurologisches Defizit“,
- „GCS-Punktwert ≤ 14“,
- „mehr als einmaliges Erbrechen“,
- „medikamentöse Antikoagulation“,
- „Alter ≥ 65 Jahre“ und
- „klinische Anzeichen einer Schädel(basis)fraktur“.

Zwei Einflussfaktoren entsprachen Evidenzstufe 2 (moderate Evidenz): „Alter ≥ 75 Jahre“ und „Hochrasanztrauma“. Die verbleibenden Einflussfaktoren wurden den Evidenzstufen 3–4 zugeordnet bzw. als inkonsistente Evidenz eingestuft (Zusatzmaterial online: Supplement 5 – MINORS; Zusatzmaterial online: Supplement 8 – Multivariate Regressionsanalysen Primärstudien).

Im VC wurden für F1 zwei Empfehlungen untersucht: Empfehlung für eine initiale cCT bei vorhandenen Einflussfaktoren oder die Empfehlung für eine initiale cCT für alle Patienten, unabhängig von individuellen Einflussfaktoren (Zusatzmaterial online: Supplement 6 – *Vote counting based on direction of effect*). Sieben Studien sprachen eine Empfehlung zur Indikation zur initialen cCT anhand von individuellen Einflussfaktoren auch bei Patienten ≥ 65 Jahren aus [21, 23, 28, 35, 36, 38, 40]; vier Studien sprachen sich gegen die Empfehlung aus [22, 33, 37, 41]. Jede dieser Studien schloss auch oder ausschließlich antikoagulierte Patienten ein, was als unabhängiger Risikofaktor für eine pathologische initiale cCT identifiziert werden konnte. Die verbleibenden Studien gaben keine Empfehlung ab.

Demgegenüber empfahlen acht Studien, unabhängig von klinischen Einflussfaktoren für alle Patienten eine initiale cCT durchzuführen [22, 24, 31, 33, 37, 41, 47, 48], wobei auch hier sieben der acht Studien auch oder ausschließlich Patienten unter Antikoagulation untersuchten. Drei Studien sprachen sich gegen die automatische initiale Bildgebung bei allen Patienten aus [21, 28, 38]. Die verbleibenden Studien gaben keine Empfehlung ab.

Demnach empfehlen sieben Studien, die Indikation zur initialen Bildgebung anhand klinischer Einflussfaktoren zu stellen. Exklusive der Studien mit antikoagulierten Patienten spricht sich eine Studie für eine automatische initiale cCT bei allen Patienten aus. Acht Einflussfaktoren haben Evidenzstufe 1 oder 2 erreicht.

### F2 und F3: Indikation zur stationären Überwachung und Kontroll-cCT

Aufgrund vieler Überschneidungen der eingeschlossenen Studien wurden F2 und F3 zusammengelegt mit 21 Studien, die insgesamt 9108 Patienten (Tab. 3) untersuchten. Im MINORS wurde die Berichterstattung von 13 Studien als moderat [50–62] und von acht Studien als suffizient bewertet [26, 32, 35, 63–67] (Zusatzmaterial online: Supplement 5 – MINORS).

**Tab. 3:** F2 + F3 Studienpopulationen + MINORS-Gesamtpunktzahl

Referenz	Land	Studiendesign	Anzahl der Patienten	Durchschnittliches Alter (Jahre)	Anteil männlicher Patienten (%)	GCS-Verteilung		Unfallmechanismus Niedrigrasanztrauma (%)	MINORS Gesamtpunktzahl
							(%)		
Bauman 2017 [59]	USA	P‑U-FS	1501	79,9	39	–	*–*	–	12/16
Campiglio 2017 [63]	Italien	R‑U-FS	284	79,1	35,2	15	76,7	79,2	14/16
						14	23,2		
Cipriano 2018 [26]	Italien	P‑U-KS	206	81,5	40,8	15	99	91,3	22/24
						14	1		
Cohan 2020 [61]	USA	R‑U-KS	332	77,5	49	–	–	88,6	18/24
Covino 2021 [64]	Italien	R‑U-QS	685	75,8	48	–	–	–	20/24
Huang 2019 [52]	USA	R‑U-QS	204	71	49	15	94	–	10/16
						14	5		
						13	1		
Kaen 2010 [53]	Spanien	P‑U-QS	137	76	33	15	89	89	12/16
						14	11		
Leitner 2021 [32]	Österreich	R‑U-KS	1788	58	55,7	–	–	–	20/24
Lim 2016 [65]	Singapur	R‑U-KS	298	71	45,6	15	99	91,6	14/16
						14	1		
Macedo 2017 [54]	USA	P‑U-KS	51	–	41	15	94	–	11/16
						14	4		
						13	2		
Marques 2021 [55]	Portugal	R‑U-FS	201	81,6	43,3	–	–	99	10/16
McCammack 2015 [35]	USA	R‑U-KS	144	74	46,5	–	–	86,1	22/24
Menditto 2012 [56]	Italien	P‑U-FS	87	80,5	32	15	100	21	12/16
Mourad 2021 [62]	USA	R‑U-KS	498	76	44,4	–	–	96,6	17/24
Scantling 2017 [66]	USA	R‑U-QS	234	80,9	43,5	15	88	80	13/16
						14	12		
Schoonman 2014 [57]	Niederlande	R‑U-FS	211	77	46	–	–	73,5	12/16
Singleton 2021 (58)	USA	P‑U-KS	138	64,6	53,6	–	–	71,7	19/24
Tauber 2009 [67]	Österreich	P‑U-QS	100	81	39	15	100	96	13/16
Turcato 2022 [25]	Italien	R‑M-KS	1426	83	45,2	–	–	74,9	10/16
Valiuddin 2021 [60]	USA	R‑U-KS	287	80,1	45,6	–	–	94,1	18/24
Yun 2018 [50]	USA	R‑U-QS	296	73,5	65	15	95	–	17/24
						14	3		
						13	2		

*GCS Glasgow Coma Scale*, – nicht berichtet; Code Studiendesign: *R* retrospektiv, *P* prospektiv, *U* unizentrisch, *M* multizentrisch, *KS* Kohortenstudie, *FS* Fallserie, *QS* Querschnittsstudie, *FKS* Fall-Kontroll-Studie, *MINORS* Methodological Index for Non-Randomized Studies

Es wurde unterschieden zwischen Studien, die nur Patienten mit initial unauffälliger cCT einschlossen, und Studien, die auch oder ausschließlich Patienten mit initial auffälliger cCT untersuchten.

Zwölf Studien untersuchten die Inzidenz neu aufgetretener Pathologien nach initial unauffälliger cCT; alle Patienten standen unter Antikoagulation [51–53, 55–57, 60, 61, 64–67]. Neu aufgetretene Pathologien wurden in 0,3–0,6 % der Fälle beschrieben. Neurochirurgische Interventionen waren in 0–1,1 % der Fälle notwendig, es verstarben zwischen 0 und 1,0 % der Patienten. Kontroll-cCT wurden nach sechs [60, 61], zwölf [66] und 24 Stunden [52, 53, 55, 64] durchgeführt. Die Grundlage für cCT-Kontroll-Intervalle und Überwachungsmodalitäten bildeten in den eingeschlossenen Studien interne Vorgaben, die nicht weiter erläutert wurden, weshalb sie in der vorliegenden Studie nicht weiter analysiert werden konnten.

Neun weitere Studien untersuchten auch oder ausschließlich Patienten mit Pathologien in der initialen cCT, davon sieben Studien explizit antikoagulierte Patienten [26, 32, 35, 50, 54, 58, 59, 62, 63]. Es wurde von zwischen 2,0 und 63,7 % initial auffälligen cCT berichtet, zwei Studien schlossen ausschließlich Patienten mit Pathologien in der initialen Bildgebung ein [50, 58]. In sechs Studien wurden nach neu aufgetretener Blutung in der Kontroll-cCT keine chirurgischen Interventionen durchgeführt [26, 35, 58, 59, 62, 63]; drei Studien machten keine Angaben [32, 50, 54]. In vier Studien wurden keine Todesfälle nach verzögert identifizierter Blutung berichtet [35, 59, 62, 63], die verbleibenden Studien waren ohne Angaben. Auch für die Studien zu initial pathologischer cCT gilt, dass nicht ausreichend über cCT-Kontroll-Intervalle und Überwachungsmodalitäten berichtet wurde, um hier weiterführende Analysen zu ermöglichen.

Im VC konnten folgende Empfehlungen abgeleitet werden (Zusatzmaterial online: Supplement 6 – *Vote counting based on direction of effect*): Drei Studien empfehlen eine automatische Kontroll-cCT für alle Patienten [32, 56, 67]; elf Studien halten einen Verzicht auf die Kontroll-cCT bei neurologisch unauffälligen Patienten für möglicherweise gerechtfertigt [26, 51–53, 55, 59–62, 64, 66]. Für unauffällige Patienten wird von sieben Studien eine Überwachung ohne Kontroll-cCT für möglich erachtet oder empfohlen [26, 53, 59, 64–67]. Drei Studien empfehlen die Entlassung neurologisch unauffälliger Patienten ohne Überwachung oder Kontroll-cCT [55, 55, 60]. Aufgrund nicht berichteter Daten lassen sich keine einheitlichen Empfehlungen zu Überwachungszeiträumen, Zeitfenstern für eine Kontroll-cCT und Überwachungsmodalitäten ableiten.

### Algorithmus

**Abb. 2:**
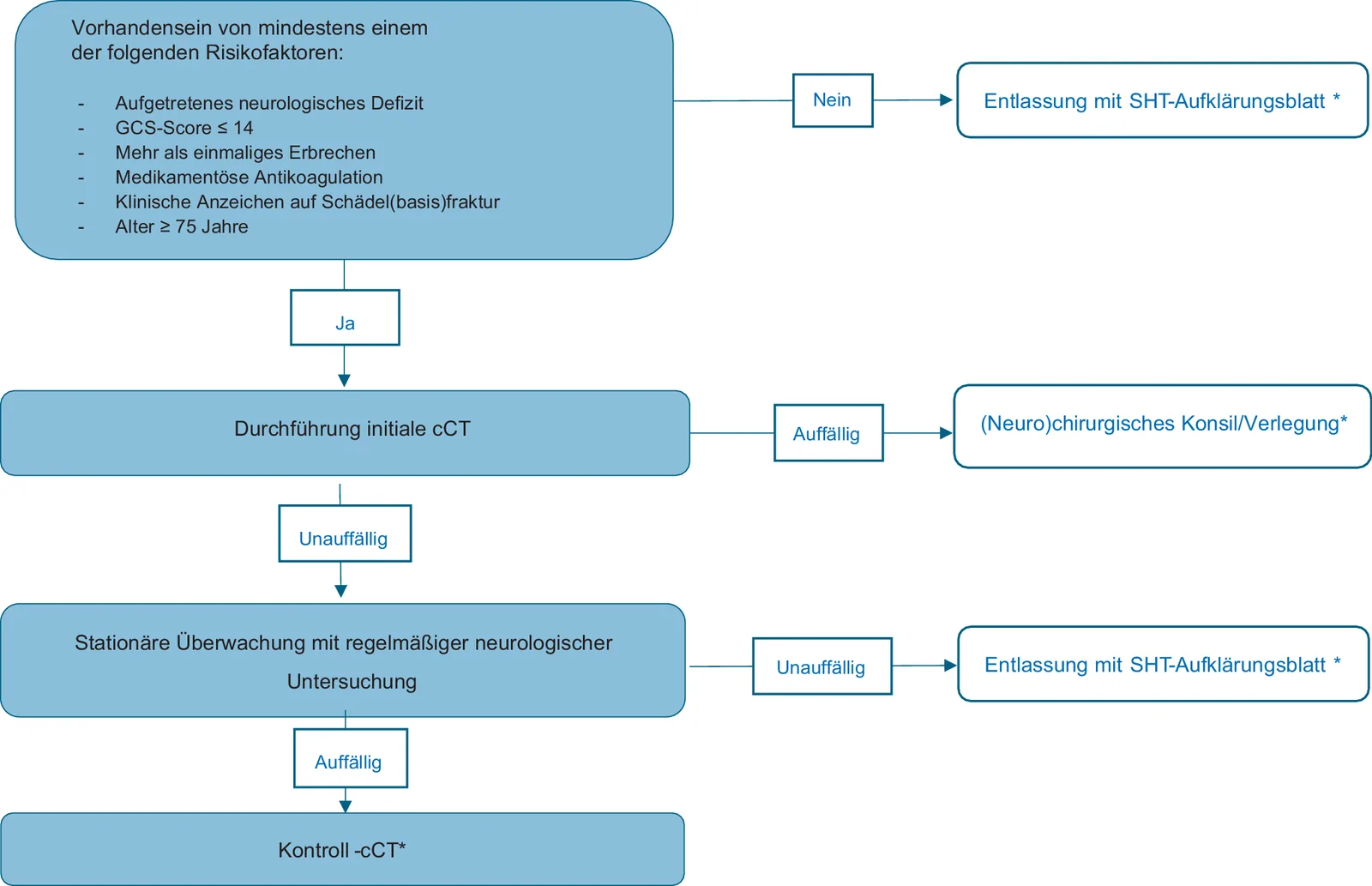
Diagnostikalgorithmus: Patienten ≥ 65 Jahre mit leichtem SHT nach Niedrigrasanztrauma. *cCT* kraniale Computertomographie; *GCS* Glasgow Coma Scale; *SHT* Schädelhirntrauma; * = keine Evidenz anhand inkludierter Studien

Abb. 2 zeigt den Algorithmus, basierend auf der identifizierten Evidenz.

## Diskussion

Der vorliegende *Systematic Review* identifizierte insgesamt 52 Studien, basierend auf den Fragestellungen F1–F3 wurden diese zugeordnet und analysiert. Die Ergebnisse ermöglichten die Erstellung eines Algorithmus, der eine evidenzbasierte Grundlage für die Vorgehensweise bei leichtem SHT bei über 65-Jährigen in der Notaufnahme ermöglicht. Im Folgenden werden die Ergebnisse der einzelnen Fragestellungen in Bezug auf den Algorithmus diskutiert.

Die identifizierte Evidenz zur Fragestellung F1 – Indikation für eine initiale cCT deutet darauf hin, dass auch bei Patienten ≥ 65 Jahren die Indikation zur initialen cCT anhand von klinischen Einflussfaktoren abhängig gemacht werden könnte. Hierfür konnten sechs klinische Einflussfaktoren identifiziert werden. Dem gegenüber steht die automatische Durchführung einer cCT bei allen Patienten ≥ 65 Jahren mit leichtem SHT – dem Vorgehen der meisten klinischen Entscheidungshilfen wie der *Canadian Head-Rule *[68] oder der *NEXUS-II*-Kriterien [69] und den *New Orleans Criteria* [70], die darüber hinaus nur unzureichend für die geriatrische Population validiert sind [71]. Alle Patienten ≥ 60 bzw. 65 Jahre erhalten in den genannten Entscheidungshilfen automatisch eine cCT, basierend auf Studien, die deutlich jüngere Patientenkollektiven mit Durchschnittsaltern zwischen 36 bis 39 Jahren untersuchten [68–70]. Im vorliegenden Algorithmus wird auch bei den ≥ 65-Jährigen – basierend auf der identifizierten Evidenz – ein Vorgehen anhand von klinischen Einflussfaktoren, analog zur Versorgung jüngerer Patienten, vorgeschlagen. Dies könnte Patienten vor unnötiger Strahlenbelastung schützen und wertvolle Ressourcen der Notfallversorgung schonen. Dem gegenüber steht das Risiko, klinisch signifikante Pathologien nicht zu erkennen, mit den daraus resultierenden Konsequenzen für Morbidität und Mortalität. Mower et al. (2024) zeigen jedoch in ihrer Evaluation der *NEXUS-II*-Kriterien, dass ältere Patienten selbst bei Verletzungsmechanismen mit geringem Risiko ein hohes Risiko für intrakranielle Verletzungen aufweisen, jedoch die klinische Beurteilung häufig sehr unzuverlässig ist, weshalb schwerwiegende Verletzungen unterschätzt werden [72]. Dementsprechend sollten die klinischen Einflussfaktoren möglichst zuverlässig intrakranielle Verletzungen identifizieren. Fünf der sechs Einflussfaktoren basieren auf starker Evidenz: „Aufgetretenes neurologisches Defizit“, „GCS-Punktwert ≤ 14“, „Mehr als einmaliges Erbrechen“, „Medikamentöse Antikoagulation“ und „Klinische Anzeichen auf Schädel(basis)fraktur“. Der verbleibende Einflussfaktor „Alter ≥ 75 Jahre“ basiert auf moderater Evidenz. Weitere untersuchte Einflussfaktoren wurden aufgrund limitierter oder inkonsistenter Evidenz nicht in den Algorithmus aufgenommen. Zum Teil handelt es sich jedoch um verbreitete und plausible Risikofaktoren (Amnesie, Bewusstseinsverlust), weshalb sie in Studien mit größeren Populationen erneut untersucht werden sollten, um die Evidenz dieser Faktoren zu untermauern. Die Sicherheit und Effektivität der Indikationsstellung zur initialen cCT anhand von Einflussfaktoren bei Patienten ≥ 65 Jahren muss vor der klinischen Anwendung in multizentrischen prospektiven Studien validiert werden. Insbesondere die pathophysiologischen Einflüsse degenerativer Prozesse, wie Hirnatrophie, ein dadurch bedingter vergrößerter Subarachnoidalraum und das größere Risiko einreißender Brückenvenen, müssen weiter untersucht werden. Welche Einbußen das Vorgehen nach Einflussfaktoren in Bezug auf die diagnostische Sensitivität hat, ist, basierend auf den vorliegenden Daten, nicht belegbar. Zur Validierung des Algorithmus sowie zur Bestimmung der Sensitivität und Spezifität des Algorithmus bedarf es multizentrischer Studien.

Um das Vorgehen bei initial unauffälliger cCT festzulegen (F2 – Indikation zur Kontroll-cCT), ist das Risiko für verzögert auftretende Pathologien relevant. Hierfür variierten die Fallzahlen in den eingeschlossenen Studien zwischen 0,3 und 0,6 %, wobei insgesamt geringe prozentuale Werte für chirurgische Interventionen und Mortalität berichtet wurden. Dies legt nahe, dass nicht jede identifizierte Pathologie klinische Relevanz hat. Entsprechend dem VC wird statt einer routinemäßigen Kontroll-cCT eine neurologische Überwachung empfohlen. Dies deckt sich mit der Empfehlung von Huang et al. (2020), die in ihrem *Systematic Review* Patienten unter Thrombozytenaggregation untersuchten [73]. Auch Colombo et al. (2021) kommen zu dem Ergebnis, dass eine routinemäßige Kontroll-cCT bei der geringen Anzahl verspätet auftretender intrakranieller Blutungen nicht zu rechtfertigen sei [74]. Aufgrund mangelnder Evidenz kann jedoch keine Angabe zur Gestaltung der neurologischen Überwachung gemacht werden.

Die Notwendigkeit einer neurologischen Überwachung (F3 – und Überwachung) wird bei asymptomatischen, neurologisch unauffälligen Patienten ≥ 65 Jahren ebenfalls infrage gestellt [75]. Die Option einer frühzeitigen Entlassung sollte in zukünftigen Studien genauer untersucht werden. Bei initial auffälliger cCT wurden Daten aus drei Studien eingeschlossen, mit Blutungsprogression in 8,8–27,1 % der Fälle. Bei geringer Datenmenge und divergierenden Handlungsempfehlungen der Primärstudien konnte für den Algorithmus kein evidenzbasierter Pfad abgeleitet werden. Stattdessen erfolgt die Empfehlung zur neurochirurgischen Vorstellung im Einklang mit den *NICE-Guidelines *[76].

„Medikamentöse Antikoagulation“ zeigte sich als Einflussfaktor mit starker Evidenz. Manche von den eingeschlossenen Studien deuten jedoch daraufhin, dass je nach Medikament der Einfluss variieren kann. Der Einfluss verschiedener Antikoagulanzien und Thrombozytenaggregationshemmer war jedoch nicht Gegenstand der vorliegenden Forschungsarbeit und, basierend auf den eingeschlossenen Studien, auch nicht beurteilbar, da diese zu inkonsistent berichten haben. Viele der eingeschlossenen Studien untersuchten zwar diese wachsende, heterogene Patientengruppe, oftmals wurden jedoch keine genauen Angaben zur Medikation gemacht. Der Einfluss verschiedener Medikamentengruppen auf die untersuchten Endpunkte und diagnostischen Fragestellungen sollte in Zukunft eingehender untersucht werden. Zusammenfassend kann postuliert werden, dass der entwickelte Algorithmus eine evidenzbasierte Grundlage für das Vorgehen bei Patienten ≥ 65 Jahre mit leichtem SHT in der Notaufnahme bildet. Zukünftige Studien sollten gezielt den Einfluss verschiedenen Antikoagulanzien und Thrombozytenaggregationshemmer auf das Blutungsrisiko bei ≥ 65 Jahre mit leichtem SHT untersuchen, um dies zukunftsweisend differenzierter adressieren zu können.

Viele der identifizierten Vorgehensweisen entsprechen vermutlich der gelebten Praxis in vielen deutschen Krankenhäusern, jedoch ist dies die erste Arbeit, die diese Vorgehensweise mit Evidenz belegt. Das Vorgehen bei einer initialen cCT bildet jedoch eine Neuerung in Bezug auf die bislang am meisten verwendeten Entscheidungstools wie z. B. *NEXUS II.* Die Ergebnisse dieser Studie bilden damit einen Vorschlag für eine evidenzbasierte Vorgehensweise ab, dies muss jedoch zunächst in einer Beobachtungsstudie validiert sowie die Sensitivität und Spezifität des Algorithmus getestet werden.

### Limitationen und Stärken

Zu den Stärken der Studie zählt das systematische methodische Vorgehen zur Erstellung des Algorithmus. Pfade, die sich als Konsequenz der evidenzbasierten Elemente ergaben, für die jedoch keine entsprechenden Studien vorliegen, sind klar als solche gekennzeichnet und sollten in Zukunft eingehender untersucht werden. Das Problem variabler Definitionen und Terminologien wird bereits bei dem Konzept des „leichten SHT“ und der Einteilung in die SHT-Schweregrade deutlich, jedoch waren auch klinische Einflussfaktoren teils nicht eindeutig definiert oder wurden in den Primärstudien unterschiedlich benannt. Es wurde sich im Algorithmus um möglichst umfassende Benennungen der Einflussfaktoren bemüht, es ist aber nicht auszuschließen, dass Einflussfaktoren teils anders interpretiert wurden als von den Autoren der Primärstudien beabsichtigt.

Eine weitere Schwäche ergibt sich aus der ungenauen oder fehlenden Berichterstattung in den Primärstudien. Dies fiel unter anderem bei ungenauen Angaben statistischer Werte auf, was zum Beispiel die Bewertung der Evidenz klinischer Einflussfaktoren limitierte. Bei den eingeschlossenen Studien handelt es sich größtenteils um retrospektive Studien, was das Risiko fehlender Daten und das Auftreten von Verzerrung erhöht [77]. Die Studien variieren teils stark in der Größe der Studienpopulation, es konnten jedoch überwiegend Studien mit Populationen > 500 Patienten eingeschlossen werden. Dies reduziert das Risiko für *Overfitting (Überanpassung) *in den Regressionsanalysen, da die Anzahl der Prädiktoren, die in einem Modell in Zusammenhang mit einem *Outcome* gebracht werden können, immer auch von der Größe der Stichprobe abhängt – komplexere multivariate Regressions-Modelle setzen größere Stichproben voraus.

Die in den *Systematic Review* eingeschlossenen Primärstudien unterscheiden nicht zwischen einer per se „gesunden“ Patientenpopulation und einer dementiell erkrankten Patientenpopulation. Jedoch ist dies für die Indikationsstellung der Diagnostik bedeutsam, da gerade bei dementiell erkrankten Patienten die Anamneseerhebung, die klinische Untersuchung sowie die Beurteilbarkeit eingeschränkt sind. Bei einer Überarbeitung des Algorithmus und auch bei der Testung des solchen sollte demnach auf diesen Aspekt geachtet werden. Bei einer Überarbeitung sollte im Speziellen nach Studien zu dementiell erkrankten Studienpopulationen mit leichten SHT gesucht werden, um diesen Aspekt adäquat abbilden zu können. Insbesondere da diese Patientenpopulation einen erheblichen Anteil, der in einer Notaufnahme mit SHT vorgestellten Patienten darstellt [78]. Bei den Daten zu F1–F3 kann davon ausgegangen werden, dass die Ergebnisse weitgehend generalisierbar sind. Hier zeigte sich ein ausgeglichenes Geschlechter- und GCS-Verhältnis, und es konnten einige multizentrische Studien eingeschlossen werden. Die Anwendbarkeit auf die deutschen Notaufnahmen wurde erhöht durch den Einschluss von Studien mit Standorten aller Versorgungsstufen.

## Ausblick

Basierend auf der Evidenz der eingeschlossenen Studien war es möglich, einen Algorithmus für die Notaufnahme für Patienten ≥ 65 Jahre mit einem leichten SHT zu erstellen. Dieser Algorithmus ermöglicht ein strukturiertes evidenzbasiertes Vorgehen in der Diagnostik dieser wachsenden Patientengruppe. Bei Vorliegen von mindestens einem der folgenden klinischen Einflussfaktoren sollte in der untersuchten Patientenpopulation eine initiale cCT durchgeführt werden: aufgetretenes neurologisches Defizit, GCS ≤ 14, mehr als einmaliges Erbrechen, medikamentöse Antikoagulation, klinische Anzeichen auf Schädel(basis)fraktur und Alter ≥ 75 Jahren. Bei Pathologien der initialen cCT wird eine neurochirurgische Vorstellung empfohlen. Ist die initiale cCT unauffällig, sollte eine neurologische Überwachung erfolgen, deren Gestaltung aufgrund mangelnder Evidenz nicht weiter definiert werden kann. Bei neurologisch unauffälligen Patienten kann auf eine Kontroll-cCT verzichtet werden. Die Berücksichtigung individueller Risikofaktoren der Patienten beim diagnostischen Vorgehen kann Überdiagnostik reduzieren und gleichzeitig personelle und finanzielle Ressourcen in der Notaufnahme schützen. Der Algorithmus sollten jedoch vor Anwendung in einer Beobachtungsstudie validiert werden.

## Fazit für die Praxis

- Basierend auf der vorhandenen Evidenz wurde ein Algorithmus für die Notaufnahme für Patienten ≥ 65 Jahren mit einem leichten SHT erstellt.
- Die Analyse wurde durch Probleme in der Berichterstattung eingeschlossener Studien, Risiko für Verzerrung und heterogenen Terminologien sowie Definitionen eingeschränkt.
- Die Evidenz legt nahe, dass die initiale cCT ggf. risikofaktorenbasiert durchgeführt werden kann.
- Die Berücksichtigung mittels individueller Risikofaktoren der Patienten kann Überdiagnostik reduzieren und gleichzeitig personelle und finanzielle Ressourcen in der Notaufnahme schützen.

## Supplementary Information

ESM1: Suchstrategie

ESM2: Evidenztabellen

ESM3: Bewertungsschema Evidenz

ESM4: Regressionsanalysen Primärstudien

ESM5: MINORS

ESM6: Vote counting based on direction of effect

ESM7: Sonstige Publikationen

ESM8: Multivariate Regressionsanalysen Primärstudien

## Data Availability

Alle Daten, die die Ergebnisse dieser Studie stützen, sind in der Veröffentlichung und dem ergänzenden Online-Zusatzmaterial enthalten.

## Abkürzungen

CT: Computertomographie
cCT: Kraniale Computertomographie
FKS: Fall-Kontroll-Studie
FS: Fallserie
GCS: *Glasgow Coma Scale*
*HWS*: *Halswirbelsäule*
*ISS*: *Injury Severity Score*
KS: Kohortenstudie
M: Multizentrisch
MINORS: *Methodological Index for Non-Randomized Studies*
*mTBI*: *Mild traumatic brain injury*
*PRISMA*: *Preferred Reporting Items for Systematic reviews and Meta-Analyses*
P: Prospektiv
*QE*: *Quantile estimation*
*QS*: *Querschnittstudie*
*R*: *Retrospective*
*SHT*: *Schädelhirntrauma*
*VC*: *Vote counting based on the direction of effect*
U: Unizentrisch
WHO: Weltgesundheitsorganisation
