# Supplementary material for: Diagnostic algorithm for the management of patients ≥ 65 years with a mild traumatic brain injury in the emergency department
Source: Unfallchirurgie (Heidelb). 2026 Aug 3;129(10):696–708. [Article in German] doi: 10.1007/s00113-026-01737-4 (PMC13601116; doi:10.1007/s00113-026-01737-4)
Supplement: Supplementary file 1 [file 113_2026_1737_MOESM1_ESM.pdf]

Appendix 1- Suchstrategie

F1 – F3 in MEDLINE

1. head[tiab] OR brain[tiab] OR cranial[tiab]

2. CT[tiab] OR "Computed Tomography"[tiab] OR "tomography, x-ray computed"[mh] OR "craniocerebral trauma/diagnostic imaging"[mh]

3. #1 AND #2

4. "mild brain injury"[TIAB] OR "mild head trauma"[TIAB] OR "minor head injury"[TIAB] OR "minor head trauma"[TIAB] OR "mild traumatic brain injury"[TIAB] OR "mild head injury"[TIAB] OR "concussion mild"[TIAB] OR "brain concussion"[mh] OR "brain injuries"[mh]

4. #3 AND #4

07.03.2022: 8149 Hits
