# Supplementary material for: Diagnostic algorithm for the management of patients ≥ 65 years with a mild traumatic brain injury in the emergency department
Source: Unfallchirurgie (Heidelb). 2026 Aug 3;129(10):696–708. [Article in German] doi: 10.1007/s00113-026-01737-4 (PMC13601116; doi:10.1007/s00113-026-01737-4)
Supplement: Supplementary file 2 [file 113_2026_1737_MOESM2_ESM.pdf]

Appendix 2 – Evidenztabellen

F1

(1) Reference, (2) study type, (3) OCEMB Level of Evidence, (4) study aim, (5) country	Inclusion criteria, exclusion criteria	Study population	(1) Incidence intracranial lesions (+ surgical intervention, mortality) (2) results indication cCT	(1) Critical Appraisal, (2) Author's conclusion
(1) Alrajhi, K. N., Perry, J. J., & Forster, A. J. (2015). Intracranial bleeds after minor and minimal head injury in patients on warfarin. The Journal of emergency medicine, 48(2), 137-142.<br><br>(2) retrospective multicenter cohort study<br><br>(3) LoE 3<br><br>(4) „The objectives of this study were to (…) 2) compare the clinical features associated with patients with and without intracranial bleeding."	Inclusion: minor or minimal head trauma, warfarin therapy, INR ≥ 1.5, GCS ≥ 13<br><br>Exclusion: penetrating injury, new FND, history of structural brain abnormalities, previous intracranial surgeries, direct referrals to neurosurgery from peripheral hospitals	N= 176<br><br>Study site: 2 urban tertiary care EDs affiliated with University of Ottawa<br><br>Characteristics:<br>X̄ age 79.0 y. (SD 13.2);<br>39.8% male;<br>83.0% GCS 15;<br>5.1% dangerous mechanism of injury	(1) Inzidenz pos. cCT:<br>89.2% had initial cCT; 15.9% had ICH.<br>- 21.9% ICH in minor head injuries (GCS 13-15, LOC, amnesia or confusion)<br>- 4.8% ICH in minimal head injuries (GCS 15, no LOC, amnesia etc.)<br><br>Of pat. with ICH:<br>- 17.9% had neurosurgical intervention<br>- 7.1% died<br><br>4 RHCT in pat. with neg. initial cCT following 2 weeks: no ICH (95% CI 0 – 49.0%)<br><br>(2) Ergebnisse:	(1) MINORS: 13 von 16 Punkten<br><br>(2) Author's conclusion:<br>„The prevalence of intracranial bleeds in anticoagulated patients presenting to EDs after minor or minimal head injury on warfarin is high and carries a high morbidity and mortality. Loss of consciousness may be associated with higher rates of intracranial bleeds."

<u>(5)</u> Canada			Comparison of ICH vs. no-ICH:<br>- ICH pat. were younger (70.2y. vs. 80.3y., p=0.007)<br>ICH pat. had more LOC (28.6% vs. 10.8%, p=0.03)	
(1) Brewer, E. S., Reznikov, B., Liberman, R. F., Baker, R. A., Rosenblatt, M. S., David, C. A., & Flacke, S. (2011). Incidence and predictors of intracranial hemorrhage after minor head trauma in patients taking anticoagulant and antiplatelet medication. Journal of Trauma and Acute Care Surgery, 70(1), E1-E5.<br><br>(2) retrospective single-center cross-sectional study<br><br>(3) LoE 3<br><br>(4) „[…] to (1) determine the frequency of intracranial hemorrhage in this patient population and its impact on clinical management, (2) identify predictors of	<u>Inclusion:</u> GCS 15, taking warfarin or clopidogrel, had cCT, included in trauma registry (seen by trauma service)<br><br><u>Exclusion:</u> -	<u>N=</u> 141<br><br><u>Study site:</u> trauma registry of a Level 2 trauma center<br><br><u>Characteristics:</u><br>$\bar{X}$ age 79y. (range 36-101y.);<br>47.5% male;<br>91.5% falls;<br>59.57% warfarin,<br>25.53% AP therapy,<br>14.89% combined therapy	<u>(1) Inzidenz pos. cCT:</u><br>29% ICH (n=41), 5 neurosurgical interventions, 4 died (2 not because of ICH)<br><br><u>(2) Ergebnisse:</u><br>LOC was the only predictor for a positive cCT (Wald = 7.468, β = 1.179, p = 0.008)	(1) MINORS: 12 von 16 Punkten<br><br><u>(2) Author's conclusion:</u> „[…] head CTs should be strongly considered in these patients, particularly if there is a history of LOC. There was no statistically significant difference between antiplatelet versus anticoagulant usage and a positive CT result."

positive imaging findings […]“<br><br>(5) USA				
(1) Brown, A. J., Witham, M. D., & George, J. (2011). Development of a risk score to guide brain imaging in older patients admitted with falls and confusion. The British Journal of Radiology, 84(1004), 756-757.<br><br>(2) prospective single-center case-series<br><br>(3) LoE 4<br><br>(4) „We therefore developed a risk scoring system […] for use in older patients admitted to hospital with a fall who are also confused.“<br><br>(5) United Kingdom	Inclusion: admitted for fall, confused, received cCT<br><br>Exclusion: -	N= 66<br><br>Study site: large medical admission unit<br><br>Characteristics:<br>$\overline{X}$ age 74,8 y. (SD 12.0; range 42 – 97y.);<br>82% >65y.;<br>59% male;<br>64% lived in own home	(1) Inzidenz pos. cCT:<br>20% sign. new pathology on cCT<br><br>(2) Ergebnisse:<br>Pos. associated with pos. cCT (p<0.2):<br>- atrial fibrillation (+1P.)<br>- history of falls (+1P.),<br>- face/scalp trauma (+1P.),<br>- new FND (+3P.),<br>- warfarin (+1P.),<br>- dementia (+1P).,<br>- GCS<14 (+1P.),<br>- antecedent dementia (-1P.)<br><br>Cut-off: 3 P.; sens. 83%, spec. 89%, PPV 63%, NPV 96%	(1) MINORS: 12 von 16 Punkten<br><br>(2) Author’s conclusion:<br>„The score […] has a high negative predicfive value […]. A score below three therefore potenfially allows CT scanning to be safely omitted […] risk score requires validation […] Until this is done, our risk score should not be adopted into practice“
(1) Cipriano, A., Park, N., Pecori, A., Bionda, A., Bardini, M., Frassi, F., ... & Ghiadoni, L. (2021). Predictors of post-traumatic complication of mild brain	Inclusion: >18y., mTBI (GCS 13-15), pat. on OAT, first ED access fort hat trauma<br><br>Exclusion: presentation >48h. after trauma,	N= 473<br><br>Study site: ED of a level 2 trauma center<br>All pat. had initical cCT within 6h after accessing ED.	(1) Inzidenz pos. cCT:<br>10.6% immediate ICH (95% CI 8.1 – 13.7); n=50 (DOAK=17, VKA =33). 3 died (prevalence 0.6%), 2 neurosurgical interventions	(1) MINORS: 24 von 24 Punkten<br><br>(2) Author’s conclusion: „In conclusion, the observed

injury in anticoagulated patients: DOACs are safer than VKAs. Internal and Emergency Medicine, 16, 1061-1070.<br><br><u>(2)</u> prospective single-center cohort study<br><br><u>(3)</u> LoE 3<br><br><u>(4)</u> „Therefore, the aim of our study was to compare risk of bleeding in individuals on DOACs or VKAs as well as to ascertain potential factors that may allow risk stratification.“<br><br><u>(5)</u> Italien	ineffective OAT (inadequate VKA intake > 1 week before trauma or >24h. from last dose of DOAK), INR < 1.5 in VKA users, heart valve prosthesis as indiciation for OAT, associated with AP therapy	Pat. with neg. initial cCT remained in ED for observation (neurological exam every 4-6h. for 24h.). Asymptomatic pat. with GCS 15 were discharged after 24h. without RHCT<br><br><u>Characteristics:</u><br>X̅ age 81.8y. (SD 8.7y.);<br>43.6% male;<br>77.2% accidental fall<br>43.8% VKA<br>- 98.1% warfarin,<br>- 1.9% acenocoumarol<br>56.2% DOAK<br>- 35.5% apixaban,<br>- 28.2% rivaroxaban,<br>- 32.0% dabigatran,<br>- 4.5% edoxaban	- Incidence pos. cCT in GCS 15: 10.2%<br>- Incidence pos. cCT in GCS 13-14: 27.3%<br><br>Incidence of delayed ICH in 1 month following trauma was 1.0% (n=4). 3 pat. had delayed ICH during 24h. observation without symptoms; 1 pat. 8 d. later, died<br><br><u>(2) Ergebnisse:</u><br>Independent RF for ICH in multivariate regression analysis:<br>- PTA (p=0.009)<br>- Trauma above clavicles (p=0.004)<br>- High blood glucose (p=0.004)<br>- High blood pressure at arrival (p=0.019)<br>- Low prothrombin activity (p=0.020)	prevalence rate of immediate intracranial hemorrhagic complications in patients on OAT suffering an MTBI is relatively high and clinically meaningful with more attention to be paid to subjects on VKAs, those with high blood pressure and hyperglycemia at admission, post-traumatic amnesia, low prothrombin activity, and evidence of trauma above the clavicles.“
(1) Cipriano, A., Pecori, A., Bionda, A. E., Bardini, M., Frassi, F., Leoli, F., ... & Santini, M. (2018). Intracranial hemorrhage in anticoagulated patients with	<u>Inclusion:</u> >18y., mTBI (blunt head injury with GCS 13-15), on OAT, single patient visit at the ED for trauma	<u>N=</u> 206<br><br><u>Study site:</u> ED of a level 2 trauma center<br>Hospital protocol: MTBI on OAT receive inital cCT +	<u>(1) Inzidenz pos. cCT:</u><br>11.2% immediate ICH on initial cCT (95% CI 6.5 – 15.5%);<br>in those with immediate ICH 4.3% mortality;	<u>(1)</u> MINORS: 22 von 24 Punkten<br><br><u>(2) Author’s conclusion:</u><br>„High-energy impact, evidence of trauma above

mild traumatic brain injury: significant differences between direct oral anticoagulants and vitamin K antagonists. Internal and Emergency Medicine, 13, 1077-1087.<br><br>(2) prospective single-center cohort study<br><br>(3) LoE 3<br><br>(4) „[…] to evaluate the effectiveness and safety of a clinical management protocol according to which patients with MTBI on OAT undergo a CT scan at presentation […]to identify possible pre-traumatic bleeding risk factors that might predict a worse outcome in patients on OAT suffering MTBI.“<br><br>(5) Italien	<u>Exclusion:</u> presentation >48h. after trauma, ineffective OAT (inadequate VKA intake > 1 week before trauma or last dose of DOAC > 24h.), INR < 1.5<br><br>Exclusion to long-term follow-up: immediate traumatic ICH at initial cCT	RHCT after 24 h. of observation (neurological exam every 4-6h.). GCS 15 and asymptomatic: no RHCT after 24 h. observation<br><br><u>Characteristics:</u><br>$\bar{X}$ age 81.53 ± 8.44y.;<br>40.8% male;<br>91.3% accidental fall/syncope;<br>99.0% GCS 15,<br>1.0% GCS 14;<br>58.7% VKA;<br>41.3% DOAK;<br>4.9% concomitant AP therapy (3.4% ASS, 1.5% CP)	in general population 0.5% mortality<br><br><u>(2) Ergebnisse:</u><br>Sign. higher prevalence of following RF in pat. with immediate ICH:<br>- High-energy impact (RR 3.47, 95% CI 1.56 – 7.75),<br>- Trauma above the clavicles (RR 3.90, 95% CI 1.20 – 12.68),<br>- Clin. Relevant fractures (RR 2.85, 95% CI 1.33 – 6.09)<br>- PTA (RR 2.58, 95% CI 1.07 – 6.21)	the clavicles, simultaneous presence of fractures and the<br>presence of PTA are predictors of hemorrhagic complications. These factors could be more closely addressed in patients on OAT referring to ED for MTBI.“
(1) Colas, L., Graf, S., Ding, J., Bertolotti, G., Thellier, N., Budzik, J. F., & Verclytte, S. (2021). Limited benefit of	<u>Inclusion:</u> >18y., with AP/AC/DOAC therapy after mTBI (GCS 13-15)	<u>N=</u> 840<br><br><u>Study site:</u> university hospital	<u>(1) Inzidenz pos. cCT:</u><br>5.8% ICH, no surgical interventions, no death	(1) MINORS: 16 von 16 Punkten

systematic head CT for mild traumatic brain injury in patients under antithrombotic therapy. Journal of Neuroradiology.<br><br>(2) prospective single-center cross-sectional study<br><br>(3) LoE 2<br><br>(4) „[…] determine predictability of IH based on clinical data […]“<br><br>(5) Frankreich	<u>Exclusion:</u> -	<u>Characteristics:</u><br>$\overline{X}$ age 82.1y.;<br>37.9% male;<br>93.2% low energy trauma;<br>92.6% GCS 15,<br>4.9% GCS 14,<br>2.5% GCS 13	<u>(2) Ergebnisse:</u><br>no predictive factors could be found	<u>(2) Author's conclusion:</u><br>„These data raise ques-tions about the benefit of performing a CT head scan as a standard systematic practice for the management of mTBI patients takingAP/AC/DOAC.“
(1) Fabbri, A., Servadei, F., Marchesini, G., Stein, S. C., & Vandelli, A. (2010). Predicting intracranial lesions by antiplatelet agents in subjects with mild head injury. Journal of Neurology, Neurosurgery & Psychiatry, 81(11), 1275-1279.<br><br>(2) prospective single-center cohort study<br><br>(3) LoE 3	<u>Inclusion:</u> ≥10y., attended ED within 24h. of MHI (acute injury of the head, GCS 14-15)<br><br><u>Exclusion:</u> unclear mechanism of injury, unstable vital signs, GCS <14, penetrating injuries, discharge against medical advice, duplicate records for re-attending for post-concussive symptoms	<u>N=</u> 14228 (5180 > 65y.)<br><br><u>Study site:</u> ED of local health district (level 1: 48 000 children and adults per year)<br><br><u>Characteristics:</u><br>36.4% ≥65y.;<br>37.8% falls or accidental injuries;<br>24.7% antiplatelet drug use in ≥65<y.	<u>(1) Inzidenz pos. cCT:</u><br>6.1% injuries, of those 17.3% neurosurgical intervention;<br>0.6% unfavourable outcome (death, permanent vegetative state, severly disabled)<br><br><u>(2) Ergebnisse:</u><br>Sign. associated with intracranial lesions:<br>- GCS 14 (OR 17.8, 95% CI 13.3 – 23.9),	(1) MINORS: 12 von 16 Punkten<br><br><u>(2) Author's conclusion:</u> „In conclusion, antiplatelet drug therapy is a significant risk factor for the development of intracranial lesions after MHI […]antiplatelet drug use, coupled with age ≥75 years, independent of amnesia and loss of consciousness, […] together with the classical main prediction variables,

(4) „The aim of the study was to test the effect of chronic use of antiplatelet drugs on the risk of intracranial lesions in subjects with MHI."<br><br>(5) Italien			- suspected scull fracture (OR 13.9, 95% CI 8.9 – 21.7),<br>- neurodeficit (OR 115.7, 95% CI 76.9 – 174.1),<br>- LOC (3.0, 95% CI 2.3 - 3.9)<br><br>Sens. 98.4%, Spec. 41.1%.<br><br>+ age ≥75y. and antiplatelet use: Sens 99.7%, spec. 54.0%	reduces to a minimum (0.2:1000) the number of cases with undetermined intracranial lesions, with a CT ordering rate of 49% […]"
(1) Fournier, N., Gariepy, C., Prévost, J. F., Belhumeur, V., Fortier, É., Carmichael, P. H., ... & Émond, M. (2019). Adapting the Canadian CT head rule age criteria for mild traumatic brain injury. Emergency Medicine Journal, 36(10), 617-619.<br><br>(2) retrospective single-center cohort study<br><br>(3) LoE 3<br><br>(4) „[…] to evaluate the impact of increasing the CCHR age criterion to 75	<u>Inclusion:</u> confirmed mTBI 24h. prior to consultation, had cCT in ED<br><br><u>Exclusion:</u> coagulopathy, anticoagulants, no cCT, delay of >24h. between injury and consultation	<u>N=</u> 104<br><br><u>Study site:</u> ED of a level 1 trauma center<br><br><u>Characteristics:</u><br>$\bar{X}$ age 76.9 y. (SD 7.4);<br>57.7% males;<br>71.2% at least one positive CCHR-criterion;<br>40.4% dangerous mechanism of injury;<br>29.8% GCS<15 2h. postinjury; 28.8% no positive CCHR-criteria	(1) <u>Inzidenz pos. cCT:</u> 30.8% clinically important brain injury<br><br><u>(2) Ergebnisse:</u><br>- 65y. cut-off: 100% sens. (95% CI 89.1 – 100), 0% spec. (95% CI 0 – 5.0)<br>- 70y. cut-off: 100% sens. (95% CI 89.1 -100), 4.2% spec. (95% CI 0.9 – 11.7)<br>- 75y. cut-off: 100% sens. (95% CI 89.1 – 100), spec. 13.9% (95% CI 6.9 – 24.1)	(1) MINORS: 15 von 16 Punkten<br><br><u>(2) Author's conclusion:</u><br>„Increasing the CCHR age criteria to 75 years old for patients with minor head injury may be safe and could avoid unnecessary CT scans."

years old on its predictive capacity and CT usage.“<br><br>(5) Canada				
(1) Galliazzo, S., Bianchi, M. D., Virano, A., Trucchi, A., Donadini, M. P., Dentali, F., ... & Ageno, W. (2019). Intracranial bleeding risk after minor traumatic brain injury in patients on antithrombotic drugs. Thrombosis Research, 174, 113-120.<br><br>(2) retrospective single-center cohort study<br><br>(3) LoE 3<br><br>(4) „The primary goal of our investigation was to comparatively assess intracranial bleeding prevalence after mTBI with a GCS score ≥13 in patients treated with vitamin K antagonists (VKAs), direct oral anticoagulants (DOACs), antiplatelets and in patients not receiving antithrombotic	Inclusion: >18y., mTBI with GCS 13-15<br><br>Exclusion: NMH therapy	N= 1846<br><br>Study site: ED of a teaching hospital<br>Hospital protocol: urgent cCT for all pat. with GCS 13-14. No cCT mandatory in pat with GCS 15 and no pre- and posttraumatic TBI-factors<br>Coagulopathies/ AC: 24h. observation and RHCT<br><br>Characteristics:<br>median age 71y. (IQR 46-83); 50.1% male;<br>98.1% GCS 15,<br>1.6% GCS 14,<br>0.3% GCS 13;<br>66.2% no-antithrombotic use;<br>22.0% AP (ASS, ticlopidine, indobufen, CP, prasugrel, ticagrelor),<br>6.5% VKA (warfarin, acenocumarol),	(1) Inzidenz pos. cCT:<br>- 4.9% ICH on initial cCT (95% CI 3.9 – 6.2), no neurosurgical intervention, 1 pat. died<br>- 22.3% had RHCT, 3 had delayed ICH (0.7%)<br><br>(2) Ergebnisse:<br>No medication was sign. ass. with increased risk of ICH.<br><br>Independently ass. with ICH:<br>- GCS < 15 (OR 7.95, 95% CI 3.12 – 20.28),<br>- PTA (OR 6.49, 95% CI 3.57 – 11.82),<br>- Vomiting (OR 4.45, 95% CI 1.47 – 13.50).<br>- Clinical signs of cranial fractures (OR 8.41, 95% CI 2.12 – 33.33)	(1) MINORS: 20 von 24 Punkten<br><br>(2) Author's conclusion: „In summary, antithrombotic therapy based on VKAs, DOACs and/or antiplatelets does not appear to independently increase the bleeding risk after a mTBI. Other clinical predictors need to be taken into account to stratify intracranial bleeding risk in order to identify all highrisk subjects who would benefit from a CT scan after acute mTBI irrespective of any concomitant antithrombotic therapy.“

drugs. Secondary aim was to identify potential clinical predictors for intracranial bleeding complications related to a mTBI.“<br><br>(5) Italien		2.8% DOAK (apixaban, dabigatran, edoxaban, rivaroxaban),<br>2.5% double antithrombotic therapy (dual antiplatelet therapy or antiplatelet and oral anticoagulant)		
(1) Hamden, K., Agresti, D., Jeanmonod, R., Woods, D., Reiter, M., & Jeanmonod, D. (2014). Characteristics of elderly fall patients with baseline mental status: high-risk features for intracranial injury. The American Journal of Emergency Medicine, 32(8), 890-894.<br><br>(2) prospective single-center cohort study<br><br>(3) LoE 3<br><br>(4) „[…] We further sought to determine which historical and physical findings were most predictive of ICI in these patients who present at baseline mental status after a fall.“	Inclusion: ≥65y., ED with fall, baseline neurological status<br><br>Exclusion: met major trauma criteria, triaged to trauma bay, acute change in baseline neurological functioning	N= 799<br><br>Study site: Level 1 community trauma center with annual ED census of 75.000<br><br>Characteristics:<br>median age 85y. (IQR 79-90); 33.2%male;<br>62.3% unwitnessed fall;<br>76.7% presented within 6h. after fall;<br>56.3% lived in own home;<br>62.7% on AP or AC therapy	(1) Inzidenz pos. cCT:<br>3.4% (n=27) intracranial injuries on cCT. 2 Pat. had neurosurgical interventions, 1 died<br><br>(2) Ergebnisse:<br>Stat. sign. associated with ICI:<br>- LOC (OR 2.77, 95% CI 1.23 – 6.27)<br>- signs of trauma to head/face (OR 13.2, 95% CI 2.7 – 64.1)<br><br>Sens. 92.6%, spec. 40.2%, PPV 5.2%, NPV 5.2%	(1) MINORS: 22 von 24 Punkten<br><br>(2) Author’s conclusion:<br>„The incidence of CT identified ICI in elder patients who are at their baseline mental status presenting to the ED after a fall is low. Signs of trauma to the head and face or loss of consciousness is predictive of ICI. […]“<br><br>(3) Algorithmus: Gruppe 3, RF für ICH

(5) USA				
(1) Ibañez Pérez De La Blanca, M. A., Fernández Mondéjar, E., Gómez Jimènez, F. J., Alonso Morales, J. M., Lombardo, M. D. Q., & Viso Rodriguez, J. L. (2018). Risk factors for intracranial lesions and mortality in older patients with mild traumatic brain injuries. Brain injury, 32(1), 99-104.<br><br>(2) prospective single-center cohort study<br><br>(3) LoE 3<br><br>(4) „The objective of this study was to identify the risk factors for post-MTBI intracranial lesion (ICL) in over 60 year-olds and determine whether the presence of pre-existing chronic diseases contributes to modifying MTBI outcomes."	<u>Inclusion:</u> ≥60y., within 24h. of a mTBI (GCS 14-15)<br><br><u>Exclusion:</u> chron. subdural hematoma	<u>N=</u> 504<br><br><u>Study site:</u> university hospital<br><br><u>Characteristics:</u><br>X̅ age 79.37(±8.06y.);<br>35.5% males;<br>87.3% falls from <1meter;<br>83.5% GCS 15,<br>16.3% GCS 14;<br>21.7% AC therapy;<br>26.8% AP therapy	<u>(1) Inzidenz pos. cCT:</u><br>5.35% cranial fractures, 63% of these had ICL (keine Information der Gesamtinzidenz für ICL)<br><br><u>(2) Ergebnisse:</u><br>sign. RF for ICL with score:<br>- traffic accident (OR 3.05, 95% CI 1.16 – 8.01) (3P.),<br>- GCS 14 (OR 3.942, 95% CI 2.20 – 7.07) (4P.),<br>- LOC (OR 1.99, 95% CI 1.21 – 3.25) (2P.),<br>- nausea (OR 3.65, 95% CI 2.03 – 6.57) (4P.),<br>- antiplatelet treatment (OR 1.72, 95% CI 1.03 - 2.87) (2P.),<br>- SSRI/Benzodiazepine (OR 1.68, 95% CI 1.04 – 2.17) (-1P.)<br><br>≥1 P. moderate/high risk of ICL. Sens. 80,2%; spec. 46,2%; PPV 32.4%; NPV 87.9%	(1) MINORS: 14 von 16 Punkten<br><br><u>(2) Author's conclusion:</u><br>„MTBI management should be distinct in over-60 year-olds, who may not present typical symptoms, with frequent comorbidities. Knowledge of risk factors for post-MTBI ICL, associated with higher mortality, is important to support clinical decision-making."

(5) Spanien				
(1) Lee, Y. B., & Kwon, S. J. (2009). A more detailed classification of mild head injury in adults and treatment guidelines. Journal of Korean Neurosurgical Society, 46(5), 451-458.<br><br>(2) retrospective single-center cohort study<br><br>(3) LoE 3<br><br>(4) „[…] to analyze the importance of clinical findings in the detection of patients at risk for intracranial lesions after MHI.“<br><br>(5) Korea	<u>Inclusion:</u> MHI patients (GCS 13-15 at admission) with or without LOC<br><br><u>Exclusion:</u> <16y., penetrating cranial trauma, admission less than 12h.	<u>N=</u> 898<br><br><u>Study site:</u> university hospital<br><br><u>Characteristics:</u><br>$\bar{X}$ age 49.3±21.7y.;<br>26% >65y. (n=228);<br>58.8% male;<br>81.3% GCS 15,<br>11.92% GCS 14,<br>6.79% GCS 13	<u>(1) Inzidenz pos. cCT:</u><br>34% pos. cCT (n=304);<br>- 19% in GCS 15,<br>- 74% in GCS 14,<br>- 88% in GCS 13<br>28 Pat. received neurosurgical interventions<br><br><u>(2) Ergebnisse:</u><br>sign. independent risk factors for abnormal cCT:<br>- >65y. (OR 6.4, 95% CI 3.81 – 10.75)<br>- GCS (OR 11.1, 95% CI 4.22 – 29.25)<br>- LOC (OR 1.84, 95% CI 1.24 -2.71)<br>- skull fracture (OR 25.5, 95% CI 12.92 – 50.18)	(1) MINORS: 14 von 16 Punkten<br><br><u>(2) Author's conclusion:</u> „[…] GCS score, LOC, patient age (> 65 years old) and skull fracture were identified as independent risk factors in patients presenting with intracranial lesions following MHI. […]CT scan is recommended for all head injury patients […]“
(1) Leitner, L., El-Shabrawi, J. H., Bratschitsch, G., Eibinger, N., Klim, S., Leithner, A., & Puchwein, P. (2021). Risk adapted diagnostics and hospitalization following mild traumatic brain injury. Archives of orthopaedic and	<u>Inclusion:</u> >18y. mTBI (GCS 13-15) indicated for cCT and/or hospitalization<br><br><u>Exclusion:</u> -	<u>N=</u> 1788<br><br><u>Study site:</u> Level 1 trauma center<br>Initial cCT according to guidelines of German Society for Neurosurgery	<u>(1) Inzidenz pos. cCT:</u><br>>65y.: 63.7% ICH; 8.5% mortality in those with ICH<br><br><u>(2) Ergebnisse:</u><br>sign. risk factors for ICH in initial cCT >65y.:	(1) MINORS: 20 von 24 Punkten<br><br><u>(2) Author's conclusion:</u><br>„Evidence, retrieved from mild TBI cases treated at our Level I Trauma Center within a decade, allowed the

trauma surgery, 141, 619-627.<br><br>(2) retrospective single-center cohort study<br><br>(3) LoE 3<br><br>(4) „[…] analysis of representative subgroups, their risk factors predicting ICH, CT progression and mortality, to allow (1) improved risk estimation on hospitalized mild TBI patients and (2) reduction of unnecessary diagnostics and hospitalization in low-risk cases.“<br><br>(5) Österreich		(mainly findings from CCHR and NOC)<br><br><u>Characteristics:</u><br>X̄ age 58y. (±22.7y., 44.5% >65y.);<br>55.7% male	- age (OR 1.01 per year; p= 0.009),<br>- anticoagulation (OR 1.1; p=0.007),<br>- neurocranial fracture (OR 1.2; p<0.001)	identification of risk factors correlated with complications and mortality, enabling more risk-adjusted hospitalization and diagnostics.“
(1) Mack, L. R., Chan, S. B., Silva, J. C., & Hogan, T. M. (2003). The use of head computed tomography in elderly patients sustaining minor head trauma. The Journal of emergency medicine, 24(2), 157-162.	<u>Inclusion:</u> MHT (GCS 13-15), ≥65y., cCT while in ED or during the same hospital stay<br><br><u>Exclusion:</u> GCS<13, no head injury	<u>N=</u> 133<br><br><u>Study site:</u> ED at a community teaching hospital in a large metropolitan area (ED sees approx. 34,000 pat./y.)<br><br><u>Characteristics:</u><br>X̄ age 80.4y. (SD 6.9);	<u>(1) Inzidenz pos. cCT:</u><br>14.3% ICI, 3.01% neurosurgical interventions<br><br><u>(2) Ergebnisse:</u><br>- Chronic altered mental status (OR 292.1, CI 6.87 - >999)<br>no statistically sign. differences with respect to	(1) MINORS: 20 von 24 Punkten<br><br><u>(2) Author’s conclusion:</u> „At this point, due to lack of reliable clinical predictors of ICI, a head CT scan is recommended on all elderly patients sustaining MHT.“

(2) retrospective single-center cohort study<br><br>(3) LoE 3<br><br>(4) „[…]to determine whether historical or clinical criteria exist that differentiate elderly patients with MHT who sustain ICI from those who escape injury; […] to determine whether current indications for head CT scan within the general population are applicable to geriatric patients.“<br><br>(5) USA		33.8% male;<br>85% GCS 15;<br>12% GCS 14;<br>3% GCS 13;<br>75% falls	use of anticoagulants or alcohol and subsequent CT scan findings. 11/ 19 patients (58%) with ICI experienced<br>no LOC.	
(1) Mason, S., Kuczawski, M., Teare, M. D., Stevenson, M., Goodacre, S., Ramlakhan, S., ... & Rothwell, J. (2017). AHEAD study: an observational study of the management of anticoagulated patients who suffer head injury. BMJ open, 7(1), e014324.	Inclusion: ≥16y., attending ED with head trauma, taking warfarin<br><br>Exclusion: penetrating injury, head trauma following a spontaneous intracranial event	N= 3534<br><br>Study site: 33 hospital sites in England and Scotland<br><br>Characteristics: median age 79y. (IQR 12, range 18 – 101y.);<br>49.2% male;<br>91.6% fall;	(1) Inzidenz pos. cCT:<br>59.8% had cCT<br>- 5.4% intracranial abnormality likely due to injury<br>- 0.5% neurosurgery<br>- 1.0% related head injury reattendance<br>- 1.2% head injury-related death	(1) MINORS: 14 von 16 Punkten<br><br>(2) Author’s conclusion: „This study has shown that (1) head injury symptoms and GCS can be used to predict adverse outcome in anticoagulated patients suffering blunt head trauma, (2) INR does not predict

(2) Retrospective multicenter cohort study<br><br>(3) LoE 3<br><br>(4) „The study aimed to determine the rate of adverse outcome associated with head injury. The primary outcome of interest was the rate of adverse outcome […]. Identifying risk factors for adverse outcome was a secondary objective.“<br><br>(5) UK		68.7% presenting with no symptoms;<br>81.2% GCS 15,<br>7.8% GCS 14,<br>0.7% GCS 13,<br>1.7% GCS < 13,<br>8.6% not reported;<br>57.2% time to scan 1-4h.	(2) Ergebnisse:<br>- Overall adverse outcome rate: 5.9% for whole cohort<br>- Adverse outcome risk for pat. with GCS 15 and no symptoms: 2.7% (95% CI 2.1 – 3.6)<br><br>Stat. sign. RF for adverse outcomes in pat. with GCS 15:<br>- Vomiting (RR 3.00, 95% CI 1.68 – 5.41)<br>- Amnesia (RR 4.90, 95% CI 3.34 – 7.19)<br>- LOC (RR 3.50, 95% CI 2.26 – 5.41)	adverse outcome in those patients with GCS=15, (3) patients with GCS=15 and no symptoms have a low risk of adverse outcome regardless of INR (2.7%). Therefore, use of CT scanning in low-risk patients may be of limited value, but the decision to recommend CT scanning in guidance should take into account the potential benefits, harms and costs of CT scanning.“<br><br>(3) Algorithmus: Gruppe 3, RF für ICH
(1) McCammack, K. C., Sadler, C., Guo, Y., Ramaswamy, R. S., & Farid, N. (2015). Routine repeat head CT may not be indicated in patients on anticoagulant/antiplatelet therapy following mild traumatic brain injury. Western Journal of Emergency Medicine, 16(1), 43.	Inclusion: mild closed TBI (GCS 13-15, initial and follow-up (6h.) cCT, ACAP<br><br>Exclusion: -	N= 144<br><br>Study site: university hospital<br>Hospital protocol: ACAP pat. receive initial cCT and 6h. RHCT<br><br>Characteristics:<br>$\overline{X}$ age 74y. (median 77y., range 25-96y.);<br>46.5% male;<br>86.1% fall/syncope	(1) Inzidenz pos. cCT:<br>6.9% initial ICH (6 stability of findings, 3 interval worsening, 1 improved)<br><br>(2) Ergebnisse:<br>sign. associated with ICH:<br>- decreased mean GCS at presentation (p<0.001),<br>- neurological examination findings (OR 24.62, 95% CI 5.8 – 104.42),	(1) MINORS: 22 von 24 Punkten<br><br>(2) Author's conclusion: „[…] for patients with normal presentation GCS and neurological examinations, no associated LOC, on single anticoagulant therapy and no<br>antiplatelet agent, we may be able to forego initial imaging altogether. „

(2) retrospective single-center cohort study<br><br>(3) LoE 3<br><br>(4) „[…] We also aimed to assess whether or not clinical and laboratory data may be able to predict the presence or absence of ICH.“<br><br>(5) USA			- LOC (OR 9.44, 95% CI 2.57 – 34.73),<br>ASS (OR 4.74, 95% CI 1.16 – 19.36) and clopidogrel (OR 11.17, 95% CI 2.33 – 53.59) and combined treatments (OR 4.11, 95% CI 1.59 – 10.82)	
(1) Moore, M. M., Pasquale, M. D., & Badellino, M. (2012). Impact of age and anticoagulation: need for neurosurgical intervention in trauma patients with mild traumatic brain injury. Journal of Trauma and Acute Care Surgery, 73(1), 126-130.<br><br>(2) retrospective single-center cross-sectional study<br><br>(3) LoE 3<br><br>(4) „We sought to investigate the impact of age and use of anticoagulation	<u>Inclusion:</u> ≥14y., GCS 14-15, underwent neurosurgical intervention<br><br><u>Exclusion:</u> -	<u>N=</u> 101<br><br><u>Study site:</u> level 1 trauma service<br><br><u>Characteristics:</u><br>64.36% >65y., of those 55.38% on AC therapy:<br>- 7 ASS<br>- 3 CP<br>- 7 ASS & CP<br>- 18 coumadin<br>1 ASS & coumadin	<u>(1) Inzidenz pos. cCT:</u><br>-<br><br><u>(2) Ergebnisse:</u><br>- 63.7% of all pat. requiering neurosurgical interventions were ≥ 65y.<br>- Overall rate of neurosurgical intervention was 3.3% in this age group: 5.35% in those on AC, 2.18% in those not on AC<br>Mortality AC + >65y.: 27.7%	<u>(1)</u> MINORS: 20 von 24 Punkten<br><br><u>(2) Author's conclusion:</u><br>„Our study suggests that patients aged 65 years or older who present with a GCS score of 14 to 15 should undergo screening CT of the head regardless of whether or not they are taking anticoagulants.“

and antiplatelet therapy and the need for neurosurgical intervention in patients presenting with mild head injury defined as GCS score of 14 to 15.<br><br>(5) USA				
(1) Mori, K., Abe, T., Matsumoto, J., Takahashi, K., & Takeuchi, I. (2021). Indications for Computed Tomography in Older Adult Patients With Minor Head Injury in the Emergency Department. Academic Emergency Medicine, 28(4), 435-443.<br><br>(2) retrospective/prospective single-center case-control study<br><br>(3) LoE 4<br><br>(4) „aimed to develop a risk stratification rule to reduce unnecessary head CT scans in older adult patients with minor head injury […]“	<u>Inclusion:</u> ≥65y., MHI (GCS 13-15) within 24h. of trauma, chief complaint of head injury in ED, had cCT<br><br><u>Exclusion:</u> GCS<13, anticoagulant therapy, FND, seizures, epilepsy, penetrating injury, depressed fracture, unknown mechanism of injuy	<u>N=</u> 1118 (DG: 538, VG: 580)<br><br><u>Study site:</u> ED of teaching hospital<br><br><u>Characteristics:</u><br>Diagnostic group:<br>median age 81y. (75-87y.);<br>49.3% male;<br>69.7% ground-level fall;<br>70.3% GCS 15,<br>27.9% GCS 14,<br>1.9% GCS 13;<br>20.1% AP therapy	<u>(1) Inzidenz pos. cCT:</u><br>10,4% acute traumatic lesions in DG, no surgical intervention or death<br><br><u>(2) Ergebnisse:</u><br>1P for each sign. RF:<br>- high-risk mechanism of injury (OR 3.03, 95% CI 1.33 – 6.92),<br>- vomiting (OR 28.45, 95% CI 6.76 – 119.77),<br>- witnessed LOC (OR 5.47, 95% CI 1.92 – 15.56),<br>- anterogr. Amnesia (OR 3.97, 95% CI 1.15 – 13.62)<br>≥ 1P: cCT with sens. 69.6%; spec. 68.9%; PPV 20.6%; NPV 95.1%	(1) MINORS: 21 von 24 Punkten<br><br><u>(2) Author’s conclusion:</u><br>„Eligible older adult patients with minor head injury can forgo CT head imaging with high negative predictive value if they have no high-risk mechanisms of injury, vomiting, witnessed loss of consciousness, or anterograde amnesia.“

(5) Japan				
(1) Nishijima, D. K., Offerman, S. R., Ballard, D. W., Vinson, D. R., Chettipally, U. K., Rauchwerger, A. S., ... & Clinical Research in Emergency Services and Treatment (CREST) Network. (2013). Risk of traumatic intracranial hemorrhage in patients with head injury and preinjury warfarin or clopidogrel use. Academic Emergency Medicine, 20(2), 140-145.<br><br>(2) prospective multicenter cross-sectional study<br><br>(3) LoE 3<br><br>(4) „The objective of this study was to prospectively derive a clinical prediction rule to identify adult patients with mild head injury and preinjury warfarin or clopidogrel use at low risk for tICH.“	<u>Inclusion:</u> ≥18y. with preinjury warfarin or clopidogrel use (within 7d.) and blunt mild head injury (GCS 13-15)<br><br><u>Exclusion:</u> no initial cCT, transferred from outside facilities	<u>N=</u> 982<br><br><u>Study site:</u> two trauma centers, 4 community hospitals<br><br><u>Characteristics:</u><br>$\overline{X}$ age 75.4y. (SD ±12.6y.);<br>47.3% male;<br>83.65% normal mental status/GCS 15;<br>72.7% warfarin,<br>28.4% CP,<br>4.6% concomitant ASS use	<u>(1) Inzidenz pos. cCT:</u><br>6.1% immediate tICH, 12 pat. of those (20%) received neurosurgical interventions, 10 (16.7%) died<br><br><u>(2) Ergebnisse:</u><br>sign. associated with tICH:<br>- Vomiting (aOR 3.68, 95% CI 1.55 – 8.76)<br>- Abnormal mental status (aOR 3.08, 95% CI 1.60 – 5.94)<br><br>Sens. 37%, spec. 87%; PPV 16%; NPV 95%	(1) MINORS: 13 von 16 Punkten<br><br><u>(2) Author's conclusion:</u> „[…] we were unable to identify a subset of patients with mild head trauma and preinjury warfarin or clopidogrel use who are at low risk for immediate traumatic intracranial hemorrhage. We thus recommend urgent and liberal cranial computed tomography imaging in this patient population, even in the absence of clinical findings.“

(5) USA				
(1) Nishijima, D. K., Offerman, S. R., Ballard, D. W., Vinson, D. R., Chettipally, U. K., Rauchwerger, A. S., ... & Holmes, J. F. (2012). Immediate and delayed traumatic intracranial hemorrhage in patients with head trauma and preinjury warfarin or clopidogrel use. Annals of emergency medicine, 59(6), 460-468.<br><br>(2) prospective multicenter cohort study<br><br>(3) LoE 3<br><br>(4) „Therefore, we assessed the prevalence and incidence of immediate and delayed traumatic intracranial hemorrhage in patients with blunt head trauma who were receiving either warfarin or clopidogrel. Warfarin and clopidogrel cohorts were compared. […]“	<u>Inclusion:</u> ≥ 18y., in ED with blunt head trauma, preinjury warfarin or CP (within previous 7 days)<br><br><u>Exclusion:</u> pat. with known injuries transferred from outside facilities, concomitant warfarin or CP	<u>N=</u> 1064<br><br><u>Study site:</u> 2 trauma centers (level 1 and 2), 4 community hospitals<br>cCT, hospital admission, RHCT at discretion of treating physician<br><br><u>Characteristics:</u><br>X̄ age 75.4y. (SD 12.7y.);<br>47.1% male;<br>83.3% ground-level fall;<br>87.6% GCS 15,<br>97.3% GCS 13-15,<br>1.7% GCS 9-12,<br>1.0% GCS 8-3;<br>94.0% received initial cCT;<br>34.2% admitted to hospital;<br>72.2% warfarin,<br>27.8% CP,<br>4.0% concomitant ASS	<u>(1) Inzidenz pos. cCT:</u><br>- 7.0% immediate ICH<br>- 21.4% inhospital mortality after immediate ICH<br>- 17.1% neurosurgical intervention after immediate ICH<br>- 0.4% delayed ICH<br><br><u>(2) Ergebnisse:</u><br>- Prevalence of immediate ICH stat. sign. higher in pat. taking CP than warfarin (RR 2.31, 95% CI 1.48 – 3.63)<br>- Stratified analysis confirmed increased risk for immediate ICH in pat. on CP compared to pat. on warfarin across all strata (≥ 65y.; GCS 13-15; GCS 15; groundlevel fall; evidence of trauma above the clavicles; without concomitant ASS use; pat. evaluated at community hospital; warfarin pat. with INR ≥1.3; warfarin pat. with INR ≥2.0)	<u>(1)</u> MINORS: 21 von 24 Punkten<br><br><u>(2) Author's conclusion:</u> „[…] Routine cranial CT imaging is generally indicated in patients with blunt head trauma who are receiving clopidogrel or warfarin, regardless of the clinical findings.“

(5) USA				
(1) O'Brien, T., Mitra, B., Le Sage, N., Tardif, P. A., Emond, M., D'Astous, M., & Mercier, E. (2020). Clinically significant traumatic intracranial hemorrhage following minor head trauma in older adults: a retrospective cohort study. Brain injury, 34(6), 836-841.<br><br>(2) retrospective multicenter cohort study<br><br>(3) LoE 3<br><br>(4) „[…] to determine the incidence of clinically significant T-ICH in older adults with minor head trauma. The association of anticoagulation and antiplatelet therapies on the incidence of T-ICH and variables potentially influencing the use of brain CT […]"<br><br>(5) Canada	<u>Inclusion:</u> ≥65y., blunt head trauma, presenting less than 7d. postinjury<br><br><u>Exclusion:</u> signs of TBI (GCS<15, LOC, amnesia, vomiting, confusion, vertigo), transferred from other hospital, suspected basilar skull fracture, intracranial anatomic abnormalities, genetic coagulation disorders, witnessed seizures	<u>N=</u> 311<br><br><u>Study site:</u> 5 hospitals (1 with neurosurgical unit), attend to > 235,000 pat./y.<br><br><u>Characteristics:</u><br>$\bar{X}$ age 80.1±7.9y.;<br>35.7% male;<br>74% ground level fall;<br>66.2% received cCT;<br>21.2% known incognitive impairment;<br>71.1% laceration or bruising to the head;<br>22.1% AC therapy,<br>41.8% AT therapy,<br>5.1% combination of AC/AT therapy	<u>(1) Inzidenz pos. cCT:</u><br>6.4% clinically sign. T-ICH in all pat. (9.7% in those with cCT); 2 pat. needed neurosurgical intervention (1 of them refused and died)<br><br><u>(2) Ergebnisse:</u><br>6.4% clin. sign. T-ICH in Pat. with minor head trauma;<br>no association in univariate and multivariate analysis between T-ICH and<br>- AP/AC therapy,<br>- age,<br>- mechanism of injury<br>- extracranial injuries.	(1) MINORS: 12 von 16 Punkten<br><br><u>(2) Author's conclusion:</u> „[…] the incidence of clinically significant T-ICH was 6.4%. Brain CT should be considered in older adults following a head trauma, with a low threshold for CT request even in the absence of TBI signs or symptoms."

(1) Pages, P. J., Boncoeur-Martel, M. P., Dalmay, F., Salle, H., Caire, F., Mounayer, C., & Rouchaud, A. (2020). Relevance of emergency head CT scan for fall in the elderly person. Journal of Neuroradiology, 47(1), 54-58.<br><br>(2) retrospective single-center cohort study<br><br>(3) LoE 3<br><br>(4) „to identify specific clinical, epidemiological or biological criteria favoring the presence of post-traumatic lesions. […] identifying a time interval between […] trauma and the CT scan to optimize the detection of post-traumatic lesions“.<br><br>(5) Frankreich	Inclusion: ≥65y., had cCT for fall from own height<br><br>Exclusion: <65y.	N= 500<br><br>Study site: university hospital<br><br>Characteristics:<br>X̄ age 85y.(±8y.);<br>40% male;<br>35.6% antiplatelet medication;<br>30.2% anticoagulant medication;<br>mean GCS 14.8 (range 3-15);<br>45.4% returned home,<br>53.4% hospitalization	(1) Inzidenz pos. cCT:<br>7,6% PTL; 0.6% urgent surgical intervention<br><br>(2) Ergebnisse:<br>sign. association (p < 0.001) between PTL and at least one of the following:<br>- male sex (RR 2.19, 95% CI 1.28 – 1.97)<br>- past PTL (RR 7.17, 95% CI 1.98 – 26.05)<br>- consciousness impairment (RR 1.56, 95% CI 1.28 – 1.97)<br>- FND (RR 6.36, 95% CI 1.13 – 35.92)<br><br>„The ROC curve […] isolated a threshold of 5 hours from which the detection of lesions would be optimal“	(1) MINORS: 20 von 24 Punkten<br><br>(2) Author's conclusion:<br>„The systematic indication of an emergency head CT scan for trauma in elderly patients presents a low diagnostic and therapeutic yield and is not relevant. Male sex, consciousness impairment, focal neurological deficit, past history of post-traumatic brain injury and time-interval between head trauma and CT are statistically related to the presence of lesions and should therefore be taken into account.“
(1) Reddy, S., Sharma, R., Grotts, J., Ferrigno, L., & Kaminski, S. (2014). Incidence of intracranial	Inclusion: GLF as mechanism of injury, ≥ 65y., taking warfarin/CP/ASS	N= 562<br><br>Study site: Level 2 trauma center	(1) Inzidenz pos. cCT:<br>15% positive initial cCT; 2.3% mortality (sign. higher in	(1) MINORS: 19 von 24 Punkten

hemorrhage and outcomes after ground-level falls in geriatric trauma patients taking preinjury anticoagulants and antiplatelet agents. The American surgeon, 80(10), 975-978.<br><br>(2) retrospective single-center cohort study<br><br>(3) LoE 3<br><br>(4) „We performed this retrospective study to evaluate risk factors for ICH in geriatric GLF patients, including preinjury use of specific anticoagulant or antiplatelet agents."<br><br>(5) USA	<u>Exclusion:</u> -	All pat. received an initial cCT<br><br><u>Characteristics:</u><br>X̄ age 83.4 y. (SD 7.7y.);<br>46.8% male;<br>X̄ GCS 14.6 (SD 1.5);<br>38.8% warfarin,<br>16.9% CP,<br>44.3% ASS	those with ICH, p<0.001)<br><br><u>(2) Ergebnisse:</u><br>- No stat. sign. difference between anticoagulation type and rate of ICH<br>- No stat. sign. difference in admission INR in those on warfarin and rate of ICH<br>- Multivariate analysis for ICH vs non-ICH: GCS <15 (OR 2.29, 95% CI 1.32 – 3.98), LOC (OR 3.14, 95% CI 1.81 – 5.46), Hypertensions (OR 1.91, 95% CI 1.06 – 3.42)<br>- No other characteristic was sign. ass. with ICH	<u>(2) Author's conclusion:</u> „We noted that a history of hypertension, loss of consciousness (LOC), and a presenting GCS below 15 were found to be predictors for ICH."
(1) Riccardi, A., Frumento, F., Guiddo, G., Spinola, M. B., Corti, L., Minuto, P., & Lerza, R. (2013). Minor head injury in the elderly at very low risk: a retrospective study of 6 years in an emergency department (ED). The	<u>Inclusion:</u> GCS 15, only minor wounds, no FND, no history of neurological disease or neurosurgical intervention, no coagulation disorders, no oral anticoagulants, no	<u>N=</u> 2149<br><br><u>Study site:</u> ED eines Krankenhauses der lokalen Gesundheitsbehörde, ca. 450 Betten	<u>(1) Inzidenz pos. cCT:</u><br>2,18% path. acute findings on cCT; 3 pat. (0.14%) underwent neurosurgery<br><br><u>(2) Ergebnisse:</u><br>- >80y.: sign. increase to 3,33% path. cCT (OR	(1) MINORS: 20 von 24 Punkten<br><br><u>(2) Author's conclusion:</u> „In conclusion, our results suggest that elderly patients between 65 and 79 years old without risk factors

American journal of emergency medicine, 31(1), 37-41.<br><br>(2) retrospective single-center cohort study<br><br>(3) LoE 3<br><br>(4) „[…] aim of better investigating this group of patients for further risk factors and possibly selecting the use of CT.“<br><br>(5) Italien	sympotms after head injury (except pain in injury site)<br><br>Exclusion: GCS<15, dangerous mechanism of injury (u.a. fall > 1 meter), deep wounds, signs of skull fractures, neurological deficits, neurological disorders and interventions, oral anticoagulants, coagulopathy, symptoms related to injury (u.a. vomiting, LOC, diplopia), assumption of alcohol or drugs	Characteristics:<br>$\bar{X}$ age 81y. (SD 7.7);<br>44.6% male;<br>28.71% on AP therapy	5.22, 95% CI 2.21 – 12.34)<br>- 3.31% path. cCT in pat. on ASS (OR 2.01, 95% CI 1.07 – 3.74)<br><br>No correlation between time of presentation at ED after trauma and path. cCT:<br>- Mean time: 5h. (95% CI 4.76 – 5.24, SD 5,74)<br>- ≤3h.: 24 pos. cCT in 983 pat. (OR 1.24, 95% CI 0.7 – 2.22)<br>>3h.: 23 pos. cCT in 1166 pat. (OR 0.8, 95% CI 0.45 – 1.43)	linked to a MHI could be managed like younger patients because the rate of early complications detected by the CT scan is very low. […] In very old patients and in the group receiving antiplatelet therapy, neuroimaging is mandatory […]“<br><br>(3) Algorithmus: Gruppe 2, Indikation cCT nach RF
(1) Sauter, T. C., Ziegenhorn, S., Ahmad, S. S., Hautz, W. E., Ricklin, M. E., Leichtle, A. B., ... & Exadaktylos, A. K. (2016). Age is not associated with intracranial haemorrhage in patients with mild traumatic brain injury and oral anticoagulation. Journal of negative results in biomedicine, 15(1), 1-5.	Inclusion: TBI (trauma to the head with LOC, amnesia and vegetative symptoms), prehospital use of rivaroxaban, apixaban, dabigatran or phenprocoumon<br><br>Exclusion: transferred from other hospitals, INR <2 on phenprocoumon	N= 260 (n=200 with mTBI: GCS 13-15)<br><br>Study site: ED of level 1 university hospital<br><br>Characteristics (only mTBI):<br>ICH group (n=86):<br>$\bar{X}$ age 80.2y. (SD 10.3);<br>$\bar{X}$ GCS 14.4 (SD 0.7);<br>$\bar{X}$ creatinine 95.5 (SD 41.5);<br>$\bar{X}$ INR 2.79 (SD 0.97)<br><br>Non-ICH group (n=114):	(1) Inzidenz pos. cCT:<br>43% ICH in mTBI (unklar wann cCT gemacht wurde)<br><br>(2) Ergebnisse (mTBI):<br>ass. with ICH:<br>- GCS (OR 0.419, CI 0.258 – 0.680)<br>- Thromboembolic event as reason for AC therapy (OR 0.486, CI 0.257 – 0.918)	(1) MINORS: 20 von 24 Punkten<br><br>(2) Author’s conclusion:<br>„Our study showed no association between age and intracranial bleeding. Therefore, diagnostic imaging with CCT is still necessary for patients of all ages with mild TBI, especially with therapeutic anticoagulation because of thromboembolic events as

(2) retrospective single-center cross-sectional study<br><br>(3) LoE 3<br><br>(4) „We hypothesized that age may not be associated with ICH in anticoagulated patients with mild TBI and therefore tested whether age as one of the high risk factors of the Canadian head CT rule is a risk factor for ICH in a patient population with mild traumatic brain injury on oral anticoagulants.“<br><br>(5) Schweiz		X̄ age 77.3y. (SD 11.8);<br>X̄ GCS 14.8 (SD 0.5);<br>X̄ creatinine 103.7 (SD 35.2);<br>X̄ INR 2.66 (SD 1.40)	Not ass.: age, type of accident, additional ASS or CP	nearly half of anticoagulated patients with mild TBI had ICH on CCT.“
(1) Savioli, G., Ceresa, I. F., Ciceri, L., Sciutti, F., Belliato, M., Iotti, G. A., ... & Bressan, M. A. (2020). Mild head trauma in elderly patients: Experience of an emergency department. Heliyon, 6(7), e04226.<br><br>(2) retrospective single-center cohort study<br><br>(3) LoE 3	Inclusion: ≥ 18y., GCS > 13<br><br>Exclusion: clinical suspicion of depressed skull fracture, clinical signs of base skull fracture, ICH without trauma, face or neck trauma that did not affect skull	N= 2851<br><br>Study site: ED of a major trauma reference center<br><br>Characteristics:<br>X̄ age 64y.;<br>47% male;<br>54.71% falls<br><br>Elderly (≥75y., n=1094):<br>36% male;<br>77% falls;	(1) Inzidenz pos. cCT:<br>- Elderly pat. 12.1%<br>- Younger pat. 5.1%<br><br>(2) Ergebnisse:<br>- 1-year increase in age raised risk of ICH by 2% on average (OR 1023/year, p< 0.001)<br>- ICH rate increased sign. after age of 75y., by 180% (OR 2.82; p< 0.001)	(1) MINORS: 20 von 24 Punkten<br><br>(2) Author’s conclusion:<br>„The data show that the age is an<br>important independent risk factor in patients with mild head trauma. In particular age over 75 is a risk factor for worse outcomes such as intracranial bleeding,

(4) „Our primary objective was to evaluate the difference in the incidence of posttraumatic intracranial bleeding after MHI in elderly and nonelderly patients. […]“<br><br>(5) Italien		97% GCS 15, 3% GCS 14;<br>39% AP, 13% VKA, 7% DOAK, 41% no therapy<br><br>Young (<75y., n=1231):<br>60% male;<br>28% falls;<br>99% GCS 15, 1% GCS 14;<br>6% AP, 1% VKA, 1% DOAK, 92% no therapy	- Elderly had higher hospitalization rates (11.7% vs. 5.5%, p<0.05) and higher readmission rates within 30 d. (6.8% vs. 3.2%, p<0.05)	hospitalization and readmission […]“
(1) Teeratakulpisarn, P., Angkasith, P., Wannakul, T., Tanmit, P., Prasertcharoensuk, S., Thanapaisal, C., ... & Sawanyawisuth, K. (2021). What are the strongest indicators of intracerebral hemorrhage in mild traumatic brain injury?. Trauma Surgery & Acute Care Open, 6(1), e000717.<br><br>(2) retrospective single-center cohort study<br><br>(3) LoE 3<br><br>(4) „This study aimed to evaluate the predictors of ICH based on baseline	<u>Inclusion:</u> >18y., mTBI (GCS 13-15), high risk of ICH (GCS 13-14 after 2h. of observation, open skull or base of skull fracture, vomiting >2x times, FND, post-traumatic seizure, ≥60y., decrease of GCS >2 without identifiable cause, high mechanism of injury)<br><br><u>Exclusion:</u> -	<u>N=</u> 100<br><br><u>Study site:</u> university hospital<br><br><u>Characteristics:</u><br>nICHg (n=76):<br>median age 72 (IQR 63-80), 47.4% male,<br>77.03% fall;<br>10.53% alcohol consumption<br><br>ICHg (n=24):<br>median age 62 (IQR 49-71), 70.83% traffic-related injury;<br>20.83% alcohol consumption	<u>(1) Inzidenz pos. cCT:</u><br>24% ICH<br><br><u>(2) Ergebnisse:</u><br>Predictive of ICH:<br>- GCS 13-14 after 2-hour observation (aOR 18.10, 95% CI 1.91 – 171.29)<br>- Open skull or base of skull fracture (aOR 8.24, 95% CI 1.33 – 50.75)	(1) MINORS: 20 von 24 Punkten<br><br><u>(2) Author’s conclusion:</u><br>„Open skull or base of skull fracture and GCS score of 13 to 14 after 2 hours of observation were the two strongest predictors of ICH in mild TBI among the examined indicators for CT of the brain.“

characteristics/injury-related factors, indications for brain CT, and a combination of the two to find the strongest indicator.“<br><br>(5) Thailand				
(1) Turcato, G., Zaboli, A., Pfeifer, N., Maccagnani, A., Tenci, A., Giudiceandrea, A., ... & Brigo, F. (2021). Decision tree analysis to predict the risk of intracranial haemorrhage after mild traumatic brain injury in patients taking DOACs. The American Journal of Emergency Medicine, 50, 388-393.<br><br>(2) retrospective multicenter case study<br><br>(3) LoE 4<br><br>(4) „[…] the aim of the present study was to use decision tree analysis with the chi-square automatic interaction detection (CHAID) method to analyse	<u>Inclusion:</u> pat. on DOAK, evaluated for a mTBI (GCS 14-15)<br><br><u>Exclusion:</u> <18y., ED presentation >48h. after trauma, FND, ineffective anticoagulation (last DOAK intake more than 24h. before the trauma)	<u>N=</u> 1146<br><br><u>Study site:</u> 4 EDs (50,000 – 90,000 admissions per year) Hospital protocol: pat. on OAT receive initial cCT and at least 24h. of observation, possibly RHCT<br><br><u>Characteristics:</u><br>Median age 83y. (IQR 78-88y.);<br>45.7% male;<br>70.0% ground fall;<br>35.3% apixaban,<br>20.1% dabigatran,<br>10.8% edoxaban,<br>33.9% rivaroxaban;<br>77% received RHCT	<u>(1) Inzidenz pos. cCT:</u><br>- 6.5% global ICH<br>- 5.7% early ICH<br>- 1.3% delayed ICH (77% of pat. had RCHT)<br>- 1.4% requiring neurosurgery/death due to ICH<br><br><u>(2) Ergebnisse:</u><br>Stat. sign. in univariate analysis:<br>- Major trauma dynamic ($p<0.001$)<br>- Previous neurosurgery ($p=0.003$)<br>- Post-traumatic transitory LOC ($p<0.001$)<br>- PTA ($p<0.001$)<br>- GCS <15 ($p<0.001$)<br>- Visible trauma above the clavicles ($p<0.001$)<br>- Vomiting ($p<0.001$)<br>- Post-traumatic seizure ($p=0.006$)	<u>(1)</u> MINORS: 20 von 24 Punkten<br><br><u>(2) Author’s conclusion:</u> „[…] Post-traumatic amnesia and post-traumatic LOC were the strongest predictors of posttraumatic ICH and could potentially be used to guide the selection of patients to submitted to head CT. The absence of visible trauma above the clavicles, the absence of previous neurosurgery and the absence of a major trauma dynamic may be considered when considering the possible negativity of an MTBI. […]“

the role of pre- and post-traumatic risk factors in predicting the risk of post-traumatic ICH after MTBI in patients taking DOACs.“<br><br>(5) Italien			- Signs of possible fracture of the skull base (p<0.001)<br>- HAS-BLED score ≥ 3 (p=0.008)<br>- Systolic BP (p=0.028)<br><br>CHAID decision tree:<br>Most important risk factors: post-traumatic transient LOC, PTA	
(1) Turcato, G., Zannoni, M., Zaboli, A., Zorzi, E., Ricci, G., Pfeifer, N., ... & Bonora, A. (2019). Direct oral anticoagulant treatment and mild traumatic brain injury: risk of early and delayed bleeding and the severity of injuries compared with vitamin K antagonists. The Journal of emergency medicine, 57(6), 817-824.<br><br>(2) retrospective single-center cohort study<br><br>(3) LoE 3<br><br>(4) „The aim of the present study is to evaluate the	<u>Inclusion:</u> AC therapy, mTBI (GCS 13-15)<br><br><u>Exclusion:</u> mTBI occured >24h. prior to the trauma, not undergoing cCT for 24h., INR < 1.5 in VKA pat., last dose of DOAK taken >24h. before, transfer by other ED, not completing the study	<u>N=</u> 451<br><br><u>Study site:</u> ED of university hospital<br>Hospital protocol: all pat. on AC therapy have initial cCT and RHCT after 24h. observation<br><br><u>Characteristics:</u><br>84.26% >75y.;<br>47.1% male;<br>71.0% accidental fall,<br>22.0% fall due to syncope;<br>59.4% on VKA,<br>40.6% on DOAK	<u>(1) Inzidenz pos. cCT:</u><br>- 9.1% immediate ICH (5.5% in DOAK vs. 11.6% in VKA, p=0.030)<br>- 3.2% delayed ICH (no difference), no neurosurgical intervention, no death<br>- 12.0 global ICH (7.7% in DOAK vs. 14.9% in VKA, p=0.026)<br>- 1.6% neurosurgical interventions, 0.7% death due to ICH (no difference)<br><br><u>(2) Ergebnisse:</u><br>Independent RF for global ICH:<br>- VKA therapy (OR 2.327, 95% CI 1.117 – 4.847),	<u>(1)</u> MINORS: 20 von 24 Punkten<br><br><u>(2) Author’s conclusion:</u><br>„Oral anticoagulant therapy is a risk factor for ICH after minor traumatic brain injury. Patients treated with DOACs seem to have lower risk of posttraumatic bleeding compared with patients treated with VKAs, reinforcing the initial indications on the greater safety of DOACs compared with VKAs. […] Finally, even in anticoagulated patients, studying the pre- and posttrauma risk factors can help the clinician stratify

differences in the risk of developing early, delayed, and global bleeding after a minor head injury in patients treated with DOACs, as compared with those treated with VKAs.“<br><br>(5) Italien			- High-energy impact (OR 11.229, 95% CI 3.265 – 38.617),<br>- Amnesia (OR 2.814, 95% CI 1.102 – 6.556),<br>- LOC (OR 5.286, 95% CI 1.102 – 25.348),<br>- GCS < 15 (OR 4.719, 95% CI 1.938 – 11.492)<br>- Lesion above clavicles (OR 2.742, 95% CI 1.297 – 5.797)<br>- Corrected AUC for the prediction tool: 0.817	and individualize the ICH risk of each patient quickly and safely.“
(1) Yuksen, C., Sittichanbuncha, Y., Patumanond, J., Muengtaweepongsa, S., & Sawanyawisuth, K. (2018). Clinical predictive score of intracranial hemorrhage in mild traumatic brain injury. Therapeutics and clinical risk management, 213-218.<br><br>(2) retrospective single-center cohort study<br><br>(3) LoE 3<br><br>(4) „This study was aimed to evaluate which clinical	<u>Inclusion:</u> >15y., had cCT after mTBI<br><br><u>Exclusion:</u> mTBI without symptoms	<u>N=</u> 708<br><br><u>Study site:</u> ED of a university-affiliated super tertiary care hospital<br><br><u>Characteristics:</u><br>CT+ (n=100, 14.1%):<br>58% > 60y.;<br>64% male;<br>71% GCS 15,<br>7% GCS 14,<br>22% GCS 13;<br>27.0% ASS,<br>3% CP,<br>2% Warfarin<br><br>CT- (n=608, 85.6%):	<u>(1) Inzidenz pos. cCT:</u><br>14.12% pat. had positive cCT results<br><br><u>(2) Ergebnisse:</u><br>Sign. predictors for ICH (1P. each):<br>- severe headache (aOR 99.81, 95% CI 37.38 – 266.5)<br>- transient LOC (aOR 5.85, 95% CI 2.92 – 11.71)<br>- PTA (aOR 1.84, 95% CI 0.9 – 3.76)<br>- FND (aOR 10.43, 95% CI 4.97 – 21.89)<br>- Clin. signs of skull fracture (aOR 34.13, 95% CI 4.37 – 266.53)	(1) MINORS: 20 von 24 Punkten<br><br><u>(2) Author’s conclusion:</u><br>„In conclusion, a user-friendly model for predicting intracranial hemorrhage in mild TBI was developed. A clinical predictive score of .3 was associated with intracranial hemorrhage in mild TBI.“

factors are associated with intracranial hemorrhage in mild TBI patients. […]“<br><br>(5) Thailand		73.4% >60y.;<br>45.6% male;<br>91.6% GCS 15,<br>4.9% GCS 14,<br>3.5% GCS 13;<br>15.3% ASS,<br>3.5% CP,<br>3.5% Warfarin	- Base of skull fracture (aOR 139.38, 95% CI 13.64 - 1,424.24)<br><br><3P.: LR for pos. cCT 0.13 (95% CI 0.07 – 0.23),<br>3-6P.: MR for pos. cCT 1.47 (95% CI 1.03 – 2.09)<br>>6P.: HR for pos. cCT 20.61 (95% CI 12.74 – 33.33)	

Legende: LoE = Level of Evidence; INR = international normalized ratio; GCS = Glasgow Coma Scale; FND = focal neurological deficit; ED = Emergency Department; y. = years; SD = standard deviation; pos. = positive; cCT = cranial computed tomography; ICH = intracranial hemorrhage; LOC = loss of consciousness; pat. = patients; RHCT = repeated head computed tomography; neg. = negative; CI = confidence interval; AP = anti-platelet therapy; sign. = significantly; sens. = sensitivity; spec. = specificity; PPV = positive predictive value; NPV = negative predictive value; DOACS = neue orale Antikoagulantien; VKAs = Vitamin-K-Antagonisten; mTBI = mild traumatic brain injury; OAT = orale Antikoagulation; h. = hours; d. = days; RF = risk factor; PTA = posttraumatic amnesia; ASS = Acetylsalicylsäure; CP = Clopidogrel; clin. = clinically; RR = relative risk; IH = intracranial hemorrhage; AC = anticoagulation; MHI = mild head injury; OR = odd’s ratio; CCHR = canadian ct head rule; NMH = niedermolekulare Heparine; IQR = interquartile range; ICI = intracranial injury; stat. = statistically; ICL = intracranial lesion; SSRI = Selektive Serotonin-Wiederaufnahme-Hemmer; NOC = new orleans criteria; MHT = mild head trauma; ACAP = anticoagulation and/or antiplatelet therapy; DG = diagnostic group; VD = validation group; P. = point; anterogr. = anterograde; tICH = traumatic intracranial hemorrhage; aOR = adjusted odd’s ratio; AT = antithrombotic therapy; ROC = receiver operating characteristics; PTL = posttraumatic lesion; GLF = ground-level fall; ass. = associated; nICHg = non intracranial-hemorrhage-group; ICHg = intracranial hemorrhage-group; BP = blood pressure; CHAID = chi-square automatic interaction detection; AUC = area under the curve; LR = low risk; MR = moderate risk; HR = high risk

F2

(1) Reference, (2) study type, (3) OCEMB Level of Evidence, (4) study aim (repeat head CT), (5) country	Inclusion criteria, exclusion criteria	Study population	(1) Incidence intracranial lesions (+ surgical interventions, mortality), (2) results repeat head CT	(1) Critical Appraisal, (2) Author's Conclusion
(1) Bauman, Z. M., Ruggero, J. M., Squindo, S., Mceachin, C., Jaskot, M., Ngo, W., ... & Lopez, P. P. (2017). Repeat head CT? Not necessary for patients with a negative initial head CT on anticoagulation or antiplatelet therapy suffering low-altitude falls. The American Surgeon, 83(5), 429-435.<br><br>(2) prospective single-center case series<br><br>(3) LoE 4<br><br>(4) „[…] we wanted to validate the necessity of the repeat HCT in a prospective manner. We hypothesized	Inclusion: >18y., low-altitude fall (any fall less than 6 feet), on ACAP therapy within 7 d. of the fall<br><br>Exclusion: refused RHCT or treating surgeon did not obtain RHCT	N= 1501<br><br>Study site: educational community-based Level 2 trauma center<br>On hospital admission all ACAP therapy was held. After initial neg. cCT, admission to general ward with neurological checks every 2h., RHCT after 12h.<br><br>Characteristics:<br>$\bar{X}$ age 79.9 y. (SD 11.4y.);<br>39% male;<br>$\bar{X}$ GCS 14.6 (SD 1.2);<br>85% visible head trauma	(1) Inzidenz pos. cCT:<br>- 8.1% ICH on initial cCT; 10.7% mortality in this group, 0.9% in whole sample (every death occured in pat. with ICH on initial cCT, p<0.001)<br>- 0.51% delayed ICH, no surgical intervention, no TBI mortality<br><br>(2) Ergebnisse:<br>- Patients taking warfarin were 27% more likely to have pos. initial cCT (p=0.018)<br>- INR did not appear to have any stat. significance on ICH developement or neurosurgical intervention	(1) MINROS: 12 von 16 Punkten<br><br>(2) Author's conclusion:<br>„[…] we conclude that patients on ACAP therapy who suffer a low-altitude fall should undergo an initial HCT and a period of neurologic observation that does not have to exceed 12 hours. Only changes in neurologic status should trigger a repeat HCT.“

that obtaining a routine scheduled repeat HCT after an initial negative HCT for ACAP therapy patients experiencing a low-altitude fall was not necessary.“<br><br>(5) USA				
(1) Campiglio, L., Bianchi, F., Cattalini, C., Belvedere, D., Rosci, C. E., Casellato, C. L., ... & Priori, A. (2017). Mild brain injury and anticoagulants: less is enough. Neurology: Clinical Practice, 7(4), 296-305.<br><br>(2) retrospective single-center case series<br><br>(3) LoE 4<br><br>(4) „To assess the utility of a 48-hour observation protocol with 2 head CT scans at a 48-hour interval, we retrospectively examined the incidence of delayed ICH in all anticoagulated patients	<u>Inclusion:</u> ≥18y., blunt HI, no LOC or less than 30 m., no amnesia or less than 24h. PTA, GCS 13-15, preinjury use of AC medication (warfarin, NMH, DOAK, concomitant AP treatments included)<br><br><u>Exclusion:</u> -	<u>N=</u> 284<br><br><u>Study site:</u> ED of a university hospital<br>Protocol: after initial neg. cCT observation in EDOU for 48h. Evaluation every 2h. by physicians and nurses. RHCT after 48h.<br><br><u>Characteristics:</u><br>X̅ age 79.09y. (SD 11.62); 35.2% male;<br>68.3% fall from standing, 10.9% syncope;<br>76.7% GCS 15,<br>23.2% GCS 14;<br>11.3% LOC;<br>3.5% amnesia	<u>(1) Inzidenz pos. cCT:</u><br>- 2.0 % pos. initiales cCT<br>- 1.4% delayed ICH on RHCT; no neurological deterioriation, no neurosurgical intervention<br><u>(2) Ergebnisse:</u><br>Total cost (48h. observation + 2 cCT): 654€	(<u>1</u>): MINORS: 14 von 16 Punkten<br><br><u>(2) Author’s conclusion:</u><br>„The utility of a 48-hour in-hospital observation in patients with mild head injuries on OACs followed by a repeated CT scan is questionable. These patients could be safely discharged after a 24-hour observation with correct surveillance at home or monitored in the ED without systematically using a second CT scan in the absence of clinical signs of brain bleeding to substantially save resources.“

admitted to our ED over 5 consecutive years.“<br><br>(5) Italien				
(1) Cohan, C. M., Beattie, G., Dominguez, D. A., Glass, M., Palmer, B., & Victorino, G. P. (2020). Routine repeat head CT does not change management in trauma patients on novel anticoagulants. journal of surgical research, 249, 114-120.<br><br>(2) retrospective single-center cohort study<br><br>(3) LoE 3<br><br>(4) „[…] We hypothesized that the incidence of ICH-d in patients on NOACs would be low, similar to that of warfarin, and that routine repeat CTH after initial negative CTH in patients on NOACs may not be indicated.“<br><br>(5) USA	Inclusion: adult trauma pateints, on prehospital oral anticoagulants, blunt trauma, had initial cCT<br><br>Exclusion: pos. initial cCT, AP therapy alone, nonoral anticoagulation, equivocal initial cCT	N= 332<br><br>Study site: level 1 trauma center Hospital protocol: RHCT after 6h., nurses make hourly GCS assessment<br><br>Characteristics:<br>Warfarin group (N=191):<br>$\bar{X}$ age 78 y. (SE 1.0y.);<br>48% male;<br>$\bar{X}$ GCS 14.7 (SE 0.1);<br>88% falls<br><br>DOAK group (N=141):<br>$\bar{X}$ age 77y. (SE 1.1y.);<br>50% male;<br>$\bar{X}$ GCS 14.7 (SE 0.05);<br>89.2% falls	(1) Inzidenz pos. cCT:<br>- 2.6% delayed ICH in warfarin pat. (one pat. had neurosurgical intervention and died)<br>- 2.1% delayed ICH in DOAK pat. (no neurosurgical intervention, no death)<br><br>(2) Ergebnisse:<br>- Incidence rates between warfarin and DOAK group were not different for delayed ICH (p=0.77)<br>- Increased odds of developing ICH-d for ISS (OR 1.2, 95% CI 1.18 – 1.27)	(1): 18 von 24 Punkten<br><br>(3) Author’s conclusion: „Our findings suggest there is limited utility in repeat imaging for patients on NOACs after traumatic injury. Limiting unnecessary imaging in this substantial and growing population of older anticoagulated patients may save time, reduce costs, and improve the allocation of resources.“

(1) Covino, M., Manno, A., Della Pepa, G. M., Piccioni, A., Tullo, G., Petrucci, M., ... & Franceschi, F. (2021). Delayed intracranial hemorrhage after mild traumatic brain injury in patients on oral anticoagulants: is the juice worth the squeeze?. European Review for Medical and Pharmacological Sciences, 25(7), 3066-3073.<br><br>(2) retrospective single center cross-sectional study<br><br>(3) LoE 3<br><br>(4) „This study's primary endpoint is to analyze the role of anticoagulants as risk factors for developing delayed ICH after MTBI."<br><br>(5) Italien	<u>Inclusion:</u> mTBI (GCS ≥ 13, LOC < 30 Min., PTA <24h.), initial cCT neg., had RHCT after 24h.<br><br><u>Exclusion:</u> no mTBI, <18y., pregnant women, inherited coagulation disease, pos. Initial cCT	<u>N=</u> 685<br><br><u>Study site:</u> ED of an urban teaching hospital (about 77,000 pat. per year), major trauma center<br>AC pat. with mTBI protocol: prolonged observation & RHCT after 24h.<br>Pat. with mTBI and no AC: 6h. observation<br><br><u>Characteristics:</u><br>median age 79y. (IQR 64.5 – 86); 48% male;<br>30.7% on AC therapy (52.8% VKA, 47.2% DOAC);<br>65.8% high-energy trauma	<u>(1) Inzidenz pos. cCT:</u><br>- 2.2% pos. RHCT for delayed ICH in all pat. (1 neurosurgical intervention, 1 death)<br>- 2.3% delayed ICH in pat. on AC, 0.6% in non-AC pat.; not stat. sign. (p=0.371)<br><br><u>(2) Ergebnisse:</u><br>Delayed ICH sign. assoc. with deterioration during observation:<br>- GCS <15 after 6h. (p=0.002),<br>- 2≥ times vomiting (p=0.032),<br>- Epilepsy (0.022)	<u>(1):</u> MINORS: 20 von 24 Punkten<br><br><u>(2) Author's Conclusion:</u><br>„Our results underline the limited role of anticoagulation therapy as a risk factor for delayed ICH after MTBI. While the clinical observation could be justified for these patients, a control CT scan should be limited to patients experiencing a clinical deterioration."
(1) Huang, J. L., Woehrle, T. A., Conway, P., McCarty, C. A., Eyer, M. M., & Eyer, S. D.	<u>Inclusion:</u> warfarin therapy, MHI, > 18y., INR >1.2, blunt	<u>N=</u> 204<br><br><u>Study site:</u> level 2 trauma center	<u>(1) Inzidenz pos. cCT:</u><br>1.7% delayed ICH on RHCT (no neurosurgical	<u>(1):</u> MINORS: 10 von 16 Punkten

(2019). Evaluation of a protocol for early detection of delayed brain hemorrhage in head injured patients on warfarin. European Journal of Trauma and Emergency Surgery, 45, 481-487.<br><br>(2) retrospective single-center cross-sectional study<br><br>(3) LoE 3<br><br>(4) „[…] Our study was intended to assess the rate of early delayed ICH and identify specific risk factors of warfarin associated head injury that would predict those at highest risk for early delayed ICH.“<br><br>(5) USA	mechanism of trauma, GCS 13-15, neg. initiales cCT<br><br><u>Exclusion:</u> trauma >12h. before admission, INR ≤ 1.2, GCS < 13, no RHCT	Hospital protocol: warfarin pat. with initial neg. cCT are admitted for 24h. with RHCT<br><br><u>Characteristics:</u><br>$\bar{X}$ age 71 y. (SD 1.9, range 29-96);<br>49% male;<br>81% groundlevel falls;<br>94% GCS 15,<br>5% GCS 14,<br>1% GCS 13;<br>41% symptoms of head trauma;<br>87% initial cCT performed within 1h. of admission	intervention)<br><br><u>(2) Ergebnisse:</u><br>The small number of patients in this group precluded secondary analysis of associated factors for early delayed ICH	<u>(2) Author's conclusion:</u><br>„[…] our study strengthens the recommendation for ED discharge of most patients with a minor traumatic brain injury who are also anticoagulated. The follow-up head CT scan will be positive in few patients, but a clinically significant result is rare. Routine follow-up head CTs for all anticoagulated patients with mild TBI are a wasteful use of resources and<br>incur unnecessary expense.
(1) Kaen, A., Jimenez-Roldan, L., Arrese, I., Delgado, M. A., Lopez, P. G., Alday, R., ... & Lobato, R. D. (2010). The value of sequential computed tomography scanning in	<u>Inclusion:</u> >16y., MHI (GCS 14 -15), AC therapy (heparin or warfarin), trauma within 48h. before admission, normal initial cCT<br><br><u>Exclusion:</u> -	<u>N=</u> 137<br><br><u>Study site:</u> university hospital; Pat. were admitted to observation unit for 24h. after initial cCT, serial neurologic	<u>(1) Inzidenz pos. cCT:</u><br>- 1.4% delayed ICH, no neurological deterioriation, no neurosurgical intervention	<u>(1):</u> MINORS: 12 von 16 Punkten<br><br><u>(2) Author's conclusion:</u><br>„[…] we do not recommend control CT scanning on a routine basis

anticoagulated patients suffering from minor head injury. Journal of Trauma and Acute Care Surgery, 68(4), 895-898.<br><br>(2) prospective single-center cross-sectional study<br><br>(3) LoE 3<br><br>(4) „[…] we designed this study to evaluate whether it is useful to perform a control CT scan before discharge in anticoagulated patients with a MHI showing no clinical deterioration during the observation period.“<br><br>(5) Spanien		exams every 4-6h., RHCT after 20 -24h.<br><br><u>Characteristics:</u><br>$\bar{X}$ age 76y. (± 9y.);<br>33% male;<br>89% falls;<br>median interval from trauma to initial cCT 3.2h.;<br>RHCT $\bar{X}$ interval of 20h. ± 1h.; 89% GCS 15,<br>11% GCS 14	<u>(2) Ergebnisse:</u><br>Stat. sign. difference between delayed ICH vs. no delayed ICH:<br>- Antiaggregation (p = 0.01)<br>- LOC (p = 0.004)	in this population of patients. […] it seems that close neurologic observation during a period of 24 hours may suffice to rule out potential surgical complications. An exception are patients anticoagulated exposed to loss of consciousness or antiagregation treatments who could be at significantly higher risk for developing posttraumatic intracranial bleeding.“
(1) Leitner, L., El-Shabrawi, J. H., Bratschitsch, G., Eibinger, N., Klim, S., Leithner, A., & Puchwein, P. (2021). Risk adapted diagnostics and hospitalization following mild traumatic brain injury. Archives of orthopaedic and	<u>Inclusion:</u> >18y. mTBI (GCS 13-15) indicated for cCT and/or hospitalization<br><br><u>Exclusion:</u> -	<u>N=</u> 1788<br><br><u>Study site:</u> Level 1 trauma center Initial cCT according to guidelines of German Society for Neurosurgery (mainly findings from CCHR and NOC)	<u>(1) Inzidenz pos. cCT:</u><br>- >65y.: 63.7% ICH on initial cCT; 8.5% mortality in those with ICH<br>- 28.3% of those with RHCT had progredient bleeding; 6.0% neurosurgical	<u>(1):</u> MINORS: 20 von 24 Punkten<br><br><u>(3) Author's Conclusion:</u><br>„Our data suggest consideration of follow-up CT, in initially negative mild TBI cases, only if at least two of these risk

trauma surgery, 141, 619-627.<br><br>(2) retrospective single-center cohort study<br><br>(3) LoE 3<br><br>(4) „Study aim was the analysis of representative subgroups, their risk factors predicting ICH, CT progression and mortality, to allow (1) improved risk estimation on hospitalized mild TBI patients and (2) reduction of unnecessary diagnostics and hospitalization in low-risk cases.“<br><br>(5) Österreich		<u>Characteristics:</u><br>$\bar{X}$ age 58y. (±22.7y., 44.5% >65y.);<br>55.7% male;<br>44.2% RHCT	interventions, 2.5% of all cases died within 30 days<br><br><u>(2) Ergebnisse:</u><br>Sign. independent RF for progression in RHCT in >65y.:<br>- Neurocranial fracture (OR 1.15, p=0.005),<br>- SAB (OR 1.14, p<0.001),<br>- SDH (OR 1.16, p<0.001),<br>- Intracerebral bleeding (OR 1.09, p=0.006)	factors are present: age > 65, anticoagulation therapy, neurocranial fracture. […] Follow-up CT is suggested in all ICH cases following TBI since progression can never be fully ruled out in any risk constellation, which is supported by our data.“
(1) Lim, B. L., Manauis, C., & Asinas-Tan, M. L. (2016). Outcomes of warfarinized patients with minor head injury and normal initial CT scan. The American journal of emergency medicine, 34(1), 75-78.	<u>Inclusion:</u> ≥16y., MHI (GCS>13), normal initial cCT, warfarin for ≥1 week, with our without concomitant AP therapy, presenting within 48h. of trauma	<u>N=</u> 298<br><br><u>Study site:</u> ED of a 1500-bed adult tertiary hospital (annual census of 170000 pat.), receives referrals from all regions of the country Hospital protocol: initial cCT for MHI Pat. on warfarin,	<u>(1) Inzidenz pos. cCT:</u><br>- 11 RHCT, 1 abnormal in a symptomatic pat. (0.3% of entire population), requiering neurosurgery<br><br><u>(2) Ergebnisse:</u>	<u>(1)</u>: MINORS: 14 von 16 Punkten<br><br><u>(2) Author's Conclusion:</u><br>„Warfarinized patients with minor HI and a normal initial CT scan result rarely developed delayed ICH. There were

(2) retrospective single-center cohort study<br><br>(3) LoE 3<br><br>(4) „Study aim was the analysis of representative subgroups, their risk factors predicting ICH, CT progression and mortality, to allow (1) improved risk estimation on hospitalized mild TBI patients and (2) reduction of unnecessary diagnostics and hospitalization in low-risk cases.“<br><br>(5) Singapur	<u>Exclusion:</u> multiple trauma, FND, open or depressed base of skull fractures	observation, RHCT at discretion of physician<br><br><u>Characteristics:</u><br>median age 71y. (IQR 17);<br>45.6% male;<br>91.6% falls;<br>99.0% initial GCS 15,<br>1.0% initial GCS 14;<br>10.4% concomitant AP therapy;<br>56.4% asymptomatic before initial cCT;<br>99.0% admitted (4.1% to ED observational ward)	- 99.4% developed no symptoms of deterioration<br>- 3.7% had RHCT, the 0.6% symptomatic pat. included; median 2 days (IQR 2) after admission<br>- no readmissions at 2 weeks after discharge due to delayed ICH<br>- no stat. sign. difference between ≥65y. vs. <65y.; concomitant AP therapy vs. No concomitant AP therapy; supratherapeutic INR vs. No supratherapeutic INR; FFP administered vs. No FFP administered	no predictive factors of development of delayed ICH. The approach of monitoring of such patients for neurologic deterioration before a second CT was safe.“
(1) Macedo, M., Grima, J., Yangouyian, M., & Kim, B. S. (2017). Delayed Intracranial Hemorrhage in Patients Taking Warfarin with Head Trauma. Spartan Medical Research Journal, 1(2).<br><br>(2) prospective single-center cohort study<br><br>(3) LoE 3	<u>Inclusion:</u> ≥18y., MHT, taking Warfarin, ED after ground level fall<br><br><u>Exclusion:</u> <18y., not taking Warfarin	<u>N=</u> 51<br><br><u>Study site:</u> ED of a community hospital (annual ED census of 100,000 visits)<br>Hospital protocol: AC pat. with isolated HI and no other reason for admission were admitted to overnight observation unit for 24h. and RHCT<br><br><u>Characteristics:</u>	<u>(1) Inzidenz pos. cCT:</u><br>- 4% positive initial cCT (no progression on RHCT)<br>- No delayed ICH on RHCT<br><br><u>(2) Ergebnisse:</u><br>38% pat. had RHCT; 2 pat. with initial bleeding (11% of those with RHCT) had no progression	(1): MINORS: 11 von 16 Punkten<br><br><u>(2) Author's conclusion:</u><br>„Given the uncertain nature and timeline of delayed intracranial bleeds, these results indicate that effective patient and family education may be of far greater utility than costly

(4) „This study was conducted to evaluate the efficacy and utility of admission and repeat head CT scan protocol for patients who had sustained minor head injuries after a ground level fall, presented with a normal or near-normal GCS and were on Warfarin therapy.“<br><br>(5) USA		median age 71.8y. (range 47-98y.); 41% male;<br>94% GCS 15,<br>4% GCS 14,<br>2% GCS 13;<br>41% 24h. observation,<br>29% discharged;<br>27% admitted to hospital		24-hour observation protocols. The study findings support previous works demonstrating that ongoing observations and costly repeat imaging are unwarranted in many cases and ultimately consume valuable hospital resources.“
(1) Marques, R. S. F., Antunes, C., Machado, M. J., Ramos, R., Duarte, N., Oliveira, L., ... & Sousa, N. (2021). Reappraising the need for a control CT in mild head injury patients on anticoagulation. European Journal of Trauma and Emergency Surgery, 47, 1461-1466.<br><br>(2) retrospective single-center case series<br><br>(3) LoE 4	Inclusion: ≥18y., anticoagulated, head trauma, initial cCT neg.<br><br>Exclusion: <18y., initial cCT pos., INR < 1.2 in case of warfarin or acenocoumarol	N= 201<br><br>Study site: university hospital, first cCT around 4h. after trauma (RHCT around 24h. after trauma)<br><br>Characteristics:<br>$\bar{X}$ age 81.6y.;<br>43.3% male;<br>99% low energy injury mechanism;<br>57.5% warfarin,<br>38% DOAC	(1) Inzidenz pos. cCT:<br>- 1.67% delayed ICH on RHCT (no surgical intervention, no death)<br><br>(2) Ergebnisse:<br>algorithm proposed for RHCT (keine Information, wie er erstellt wurde)	(1): MINORS: 10 von 16 Punkten<br><br>(2) Author's conclusion:<br>„[…] de novo intracranial hemorrhages in control head CT of anticoagulated patients are infrequent. We therefore suggest a new decision algorithm for these patients (Fig. 1); we propose that, with certain exceptions, such patients may be discharged after the first CT without intracranial hemorrhage providing that they are

(4) „[…] to evaluate the prevalence of de novo intracranial hemorrhages in control head CTs, and the relevance of this procedure in patients on anticoagulation therapy that suffer from head trauma but do not display neurological deterioration.“<br><br>(5) Portugal				accompanied by a caregiver and properly informed about red flags.“
(1) McCammack, K. C., Sadler, C., Guo, Y., Ramaswamy, R. S., & Farid, N. (2015). Routine repeat head CT may not be indicated in patients on anticoagulant/antiplatelet therapy following mild traumatic brain injury. Western Journal of Emergency Medicine, 16(1), 43.<br><br>(2) retrospective single-center cohort study<br><br>(3) LoE 3	Inclusion: mild closed TBI (GCS 13-15, initial and follow-up (6h.) cCT, ACAP<br><br>Exclusion: -	N= 144<br><br>Study site: university hospital Hospital protocol: pat. on ACAP with mTBI and initial neg. cCT receive 6h. RHCT<br><br>Characteristics:<br>X̅ age 74y. (median 77y., range 25-96y.);<br>46.5% male;<br>86.1% fall/syncope	(1) Inzidenz pos. cCT:<br>- 6.9% initial ICH (6 stability of findings, 3 interval worsening, 1 improved)<br>- 0.7% delayed ICH (n=1), no neurosurgical intervention<br><br>(2) Ergebnisse:<br>No evidence of trauma-related re-admission 30 d. after event	(1): MINORS: 22 von 24 Punkten<br><br>(2) Author’s conclusion: „a six-hour follow-up HCT is of extremely low yield in general and is likely not indicated, as our data suggests that clinically significant delayed ICH does not occur.“

(4) „[…] to examine the current protocol at our institution for the evaluation of patients on ACAP agents presenting to the ED after mild TBI, including the overall yield of the routine six hour<br>follow-up HCT to exclude delayed ICH. […]“<br><br>(5) USA				
(1) Menditto, V. G., Lucci, M., Polonara, S., Pomponio, G., & Gabrielli, A. (2012). Management of minor head injury in patients receiving oral anticoagulant therapy: a prospective study of a 24-hour observation protocol. Annals of emergency medicine, 59(6), 451-455.<br><br>(2) prospective single-center case series<br><br>(3) LoE 4<br><br>(4) „We evaluated the incidence of delayed intracranial bleeding in	Inclusion: ≥14y., mTBI (GCS 14-15), presenting within 48h. after trauma, taking warfarin for at least 1 w., ISS <15<br><br>Exclusion: pos. initial cCT	N= 87<br><br>Study site: Level 2 trauma center (annual ED census of 57,000)<br>Guideline: AC pat. with mTBI receive initial cCT, 24h. observation (ED observation unit), after that RHCT recommended<br><br>Characteristics:<br>median age 82y. (range 60-93; SD 9y.);<br>32% male;<br>100% GCS 15,<br>21% syncope,<br>79% accidental trauma	(1) Inzidenz pos. cCT:<br>- 16% pos. initial cCT<br>- 6% (95% CI 1%-11%; n=5) had delayed ICH; 3 admitted, 1 had craniotomy<br>(2) Ergebnisse:<br>- Median time trauma to cCT: 4.5h.; trauma and RHCT 24.5h.<br>- 1 pat. with delayed ICH showed neurologic deterioration<br>- 2 pat. with neg. RHCT readmitted after 2 and 8 d., had subdural hematoma; no neurosurgery	(1): MINORS: 12 von 16 Punkten<br><br>(2) Author’s conclusion: „In conclusion, our data support the general effectiveness of the European Federation of Neurological Societies’ recommendations for 24-hour observation followed by a repeated head CT scan for anticoagulated patients with a minor head injury. This protocol will identify most occurrences of delayed bleeding.“

anticoagulated patients after a minor head injury who were treated according to European guidelines in our emergency department (ED) observational unit.“<br><br>(5) Italien			- Most predicitive of delayed ICH: INR ≥ 3 (RR 14, 95% CI 4 – 49)	
(1) Mourad, M., Senay, A., & Kharbutli, B. (2021). The utility of a second head CT scan after a negative initial CT scan in head trauma patients on new direct oral anticoagulants (DOACs). Injury, 52(9), 2571-2575.<br><br>(2) retrospective single-center cohort study<br><br>(3) LoE 4<br><br>(4) „[…] In order to assess the utility of repeat imaging in this growing patient population, we undertook a retrospective review of all consecutive head traumas patients on DOAC therapy presenting to our institution.“	<u>Inclusion:</u> adult patients, confirmed or suspected blunt head trauma, DOAC, had at least one cCT<br><br><u>Exclusion:</u>n<18y. transferred outside of hospital Health system	<u>N=</u> 498<br><br><u>Study site:</u> Level 3 trauma center Hospital protocol: DOAC pat. within 12h. of head trauma are admitted for observation after initial cCT; RHCT after 12h. of observation<br><br><u>Characteristics:</u><br>X̄ age 76y. (SD 14 y.);<br>44.4% male;<br>96.6% low height or ground level fall;<br>X̄ GCS in pat. with neg. first cCT: 14.9 (SD 0.6)<br>X̄ GCS in pat. with pos. first cCT: 14.8 (SD 0.4)<br>87.7% of pat. with neg. first cCT had RHCT	<u>(1) Inzidenz pos. cCT:</u><br>- 3.8% pos. initial cCT<br>- 0.5% pos. RHCT in those with initial neg. cCT<br><br><u>(2) Ergebnisse:</u><br>- Initial cCT pos. Pat. had sign. higher median ISS than initial cCT neg Pat. (6.0 vs 1.0, p<0.001)<br>- Type of DOAK, GCS and type of trauma mechanism did not have stat. sign. impact on likelihood of initial pos. cCT<br>- RHCT pos. pat. had sign. higher median ISS than RHCT neg. pat. (11.5 vs 1.0, p=0.022) and lower median GCS (12.5 vs 15, p=0.033)<br>- No stat. sign. Relationship between	<u>(1):</u> MINORS: 17 von 24 Punkten<br><br><u>(2) Author’s conclusion:</u><br>„These findings do not support routinely obtaining a repeat CT head within 24 hours after a negative initial head CT scan. Close patient follow-up after discharge might be warranted. Patients presenting with lower GCS and higher ISS had a higher chance of having an intracranial hemorrhage. However, observa- tion and selectively repeating head imaging may be beneficial in select high risk patients […]“

(5) USA			pos. RHCT and type of DOAK, sex, mechanism of injury	
(1) Scantling, D., Fischer, C., Gruner, R., Teichman, A., McCracken, B., & Eakins, J. (2017). The role of delayed head CT in evaluation of elderly blunt head trauma victims taking antithrombotic therapy. European Journal of Trauma and Emergency Surgery, 43, 741-746.<br><br>(2) retrospective single-center cross-sectional study<br><br>(3) LoE 3<br><br>(4) „We hypothesized that routine repeat head CT in the asymptomatic elderly taking antithrombotic medication would not identify clinically relevant delayed intracranial hemorrhage."<br><br>(5) USA	<u>Inclusion:</u> >65y., GCS 14-15, AC/AP therapy, had initial cCT and RHCT (12h. after admission) without prompting by change in clinical status<br><br><u>Exclusion:</u> pos. Initial cCT	<u>N=</u> 234<br><br><u>Study site:</u> level 1 trauma center Hospital protocol: all pat. >65y. with AP/AC use receive RHCT 12h. after admission<br><br><u>Characteristics:</u><br>$\bar{X}$ age 80.9y.;<br>43.5% male;<br>88% GCS 15,<br>12% GCS 14;<br>80% falls;<br>41% LOC;<br>$\bar{X}$ time to RHCT 14h.	<u>(1) Inzidenz pos. cCT:</u><br>- n=2 delayed ICH (0.85%, 95% CI 0.1 – 3.1%)<br><br><u>(2) Ergebnisse:</u><br>- both pat. with delayed ICH taking ASS, both with GCS 15, discharged to home and rehabilitation facility<br>- 3.4% died during admission<br>- No pattern was identified to predict delayed ICH	<u>(1):</u> MINORS: 13 von 16 Punkten<br><br><u>(2) Author's conclusion:</u><br>„We feel that eliminating routine repeat head CT after minor head trauma will not miss any clinically significant ICH [...] We feel that a short period of observation and repeated neurological exam should be sufficient in this population."

(1) Singleton, J. M., Bilello, L. A., Greige, T., Balaji, L., Tibbles, C. D., Edlow, J. A., ... & Rosen, C. L. (2021). Outcomes of a novel ED observation pathway for mild traumatic brain injury and associated intracranial hemorrhage. The American Journal of Emergency Medicine, 45, 340-344.<br><br>(2) prospective single-center cohort study<br><br>(3) LoE 3<br><br>(4) „[…] we sought to create an ED-based observation protocol for patients with cmTBI. We hypothesized that ED-based observation would be a safe and efficient disposition for appropriately selected patients.“<br><br>(5) USA	<u>Inclusion:</u> ≥16y., GCS ≥14, isolated HI with minor pos. cCT<br><br><u>Exclusion:</u> AC/AP use (except ASS), GCS ≤ 13, skull fracture with ass. ICH, epidural hematoma, midline shift or mass effect, multiple traumatic injuries	<u>N=</u> 138<br><br><u>Study site:</u> Level 1 trauma center (annual ED census of appr. 57,000)<br>RHCT was performed within 6-12h. when recommended by neurosurgeon<br><br><u>Characteristics:</u><br>$\bar{X}$ age 64.6y. (median 67, SD ± 20.7);<br>53.6% male;<br>80.4% transferred from outside hospitals for neurosurgical evaluation; 71.7% falls;<br>63.8% RHCT;<br>42% ASS	<u>(1) Inzidenz pos. cCT:</u><br>- 63.8% RHCT mit 6.8% (6/88) hematoma expansion, no neurosurgical intervention<br><br><u>(2) Ergebnisse:</u><br>- 81.9% pat. successfully discharged home<br>- Correlated with pathway non-completion requiring admission: inreased age (median age 79 vs. 66, p=0.02), ASS (p=0.04) but not correlated with admission due to ICH<br>- 18% admitted, 2.8% specifically for concerns related to ICH (no neurosurgical intervention)	<u>(1):</u> MINORS: 19 von 24 Punkten<br><br><u>(2) Author's conclusion:</u> „ […] cmTBI patients with normal neurologic exams, GCS ≥ 14, and without extracranial traumatic injuries or other social or acute medical issues requiring inpatient management may be safely discharged from the ED after a brief period of observation.“
(1) Tauber, M., Koller, H., Moroder, P., Hitzl, W., & Resch, H. (2009). Secondary	<u>Inclusion:</u> admitted for neurological observation in ED after mild HI (GCS 15),	<u>N=</u> 100<br><br><u>Study site:</u> level 1 trauma center	<u>(1) Inzidenz pos. cCT:</u><br>- 4 delayed ICH, none showed signs of	<u>(1):</u> MINORS: 13 von 16 Punkten

intracranial hemorrhage after mild head injury in patients with low-dose acetylsalicylate acid prophylaxis. Journal of Trauma and Acute Care Surgery, 67(3), 521-525.<br><br>(2) prospective single-center cross-sectional study<br><br>(3) LoE 2<br><br>(4) „[…]The hypothesis was stated that patients 65 years of age and older with a negative primary head computed tomography (CT)-scan would have an uneventful course with a negative follow-up head CT-scan after a minimum of 12 hours. […]“<br><br>(5) Österreich	≥65y., taking ASS regularly, initial cCT neg., systolic blood pressure <150mmHg<br><br><u>Exclusion:</u> other AP/AC therapies, moderate or severe HI, hematological or oncological diseases	Hospital protocol: 24h. observation after mTBI in pat. >65y. on ASS, RHCT after minimum of 12h. from initial cCT<br><br><u>Characteristics:</u><br>$\overline{X}$ age 81y. ± 10y.;<br>39% male;<br>96% low-velocity fall;<br>43% superficial skull injuries including wounds and lacerations	detoriation; 1 neurosurgical intervention (survived), 1 death<br><br><u>(2) Ergebnisse:</u><br>- $\overline{X}$ time to initial cCT: 6.4h. ± 3.9 h.<br>- $\overline{X}$ to RHCT 19h. ± 9h. (minimum 12h.)	<u>(2) Author’s conclusion:</u><br>„Therefore, for this selected group of patients admission for neurologic observation with RHCT after 12 hours to 24 hours is recommended. If RHCT is not possible, extended in-hospital observation for at least 48 hours seems indicated.“

(1) Turcato, G., bauman, A., Zaboli, A., Park, N., Riccardi, A., Santini, M., ... & Ghiadoni, L. (2022). Risk of delayed intracranial haemorrhage after an initial negative CT in patients on DOACs with mild traumatic brain injury. The American Journal of Emergency Medicine, 53, 185-189.<br><br>(2) retrospective multicenter cohort study<br><br>(3) LoE 3<br><br>(4) „[…] to assess the incidence of delayed ICH after 24 h from an initial CT free of injury in a large cohort of patients taking DOACs with MTBI […] to determine which trauma-related characteristics and clinical risk factors may be associated with the risk of delayed ICH in these patients.“<br><br>(5) Italien	Inclusion: mTBI (GCS 14-15), DOAC, repeat cCT after initial negative cCT after 24h. of observation in ED (or 30-day medical follow-up, not included in main analysis of the study)<br><br>Exclusion: <18y., trauma to ED > 48h., last intake of DOAC >24h. before trauma	N= 1426<br><br>Study site: 5 EDs (50000 – 900000 annual visits per ED)<br>Protocol: mTBI pat. on OAT receive initial cCT + 24h. observation; RHCT at discretion of physician<br><br>Characteristics:<br>median age 83y. (IQR 78-88y.);<br>45.2% male;<br>74.9% fall;<br>68.3% had RHCT	(1) Inzidenz pos. cCT:<br>- 6% immediate ICH<br>- 1.5% delayed ICH (no serious outcome)<br><br>(2) Ergebnisse:<br>No pat. with initial cCT 8h. after trauma had delayed ICH.<br>Associated with delayed ICH:<br>- LOC (21.4% vs. 2.4%, p=0.005),<br>- PTA (35.7% vs. 9.1%, p=0.007)	(1): MINORS: 10 von 16 Punkten<br><br>(2) Author's conclusion:<br>„Study results confirmed earlier evidence on the reduced incidence of delayed ICH in this patient group and its low impact on patient outcome […] among patients who had their first CT within 8 h of trauma, assessment of risk factors may indicate a risk profile of delayed ICH even in patients taking DOACs.“

(1) Valiuddin, H., Calice, M., Alam, A., Millard, J., Boehm, K., Valiuddin, Y., ... & Keyes, D. (2021). Incidence of traumatic delayed intracranial Hemorrhage among patients using direct oral anticoagulants. The Journal of Emergency Medicine, 61(5), 489-498.<br><br>(2) retrospective single-center cohort study<br><br>(3) LoE 3<br><br>(4) „In this study, we sought to investigate the incidence of DICH in patients with traumatic head injury using DOACs to identify patients with head trauma who can be managed without a repeat HCT and hospital ad-mission.“<br><br>(5) USA	<u>Inclusion:</u> suspected head injury, on DOAK, initial cCT neg.<br><br><u>Exclusion:</u> ICH on initial cCT, dual anticoagulant or antiplatelet therapy aside from ASS	<u>N=</u> 287<br><br><u>Study site:</u> academic community hospital, level 2 trauma center, ED annual volume approx. 52,000 cases<br>Hospital protocol: after neg. initial cCT admission to observation unit with RHCT after 6-12h.<br><br><u>Characteristics:</u><br>Median age 78.9y. (IQR 73 – 87)<br>45.6% male<br>Median GCS 15 (IQR 15-15)<br>94.1% fall	<u>(1) Inzidenz pos. cCT:</u><br>- 1.74% delayed ICH on RHCT (n=1 death attributed to ICH)<br><br><u>(2) Ergebnisse:</u><br>Correlation between age and systolic blood pressure only (Pearson correlation coefficient 0.180, p=0.003)	<u>(1):</u> MINORS: 18 von 24 Punkten<br><br><u>(2) Author's conclusion:</u><br>„[…] we found an incidence of DICH of 0.7–1.7%, showing a clinically significant rate of only 0.7%. An association was noted between DICH and higher ISS. […] taking into account lower degree of injury severity, along with low mechanism and reasonable out- patient follow-up in the future, it may be possible to use shared decision-making to develop an appropriate plan of care that entails discharging home with standard head injury precautions and no repeat HCT.“

Legende: CT = computed tomography; LoE = level of evidence; HCT = head computed tomography; ACAP = anticoagulation and/or antiplatelet therapy; d. = days; RHCT = repeated head computed tomography; cCT = cranial computed tomography; neg. = negative; h. = hours; y. = years; SD = standard deviation; GCS = Glasgow Coma Scale; ICH = intracranial hemorrhage; pos. = positive; TBI = traumatic brain injury; INR = international normalized ratio; ED = emergency department; HI = head injury; LOC = loss of consciousness; m. =

minutes; PTA = posttraumatic amnesia; AC = anticoagulation; NMH = niedermolekulare Heparine; DOAK = neue orale Antikoagulantien; AP = antiplatelet therapy; EDOU = emergency department observation unit; OAC = orale Antikoagulation; CTH = head computed tomography; ICH-d = delayed intracranial hemorrhage; NOACs = neue orale Antikoagulantien; SE = standard error; OR = odd's ratio; ISS = injury severity score; CI = confidence interval; MTBI = mild traumatic brain injury; IQR = interquartile range; MHI = mild head injury; sign. = significantly; SAB = Subarachnoidalblutung; SDH = Subduralblutung; FND = focal neurological deficit; FFP = fresh frozen plasma; w. = week; ASS = Acetylsalicylsäure; cmTBI = complicated mild traumatic brain injury

F3

(1) Reference, (2) study type, (3) OCEMB Level of Evidence, (4) study aim, (5) country	Inclusion criteria, exclusion criteria	Study population	(1) Incidence intracranial lesions (+ surgical interventions, mortality) (2) results	(1) Critical Appraisal, (2) Author's Conclusion
(1) Campiglio, L., Bianchi, F., Cattalini, C., Belvedere, D., Rosci, C. E., Casellato, C. L., ... & Priori, A. (2017). Mild brain injury and anticoagulants: less is enough. Neurology: Clinical Practice, 7(4), 296-305.<br><br>(2) retrospective single-center case series<br><br>(3) LoE 4<br><br>(4) „To assess the utility of a 48-hour observation protocol with 2 head CT scans at a 48-hour interval, we retrospectively examined the incidence of delayed ICH in all anticoagulated patients	Inclusion: ≥18y., blunt HI, no LOC or less than 30 m., no amnesia or less than 24h. PTA, GCS 13-15, preinjury use of AC medication (warfarin, NMH, DOAK, concomitant AP treatments included)<br><br>Exclusion: -	N= 284<br><br>Study site: ED of a university hospital<br>All AC-pat. with initial neg. cCT had 48h. observation, evaluation by physician/nurse every 2h.<br><br>Characteristics:<br>$\overline{X}$ age 79.09y. (SD 11.62);<br>35.2% male;<br>68.3% fall from standing,<br>10.9% syncope;<br>76.7% GCS 15,<br>23.2% GCS 14;<br>11.3% LOC;<br>3.5% amnesia	(1) Inzidenz pos. cCT:<br>- 2.0 % pos. initiales cCT<br>- 1.4% delayed ICH on RHCT; no neurological deterioriation, no neurosurgical intervention<br>(2) Ergebnisse:<br>Total cost (48h. observation + 2 cCT): 654€	(1): MINORS: 14 von 16 Punkten<br><br>(2) Author's conclusion:<br>„The utility of a 48-hour in-hospital observation in patients with mild head injuries on OACs followed by a repeated CT scan is questionable. These patients could be safely discharged after a 24-hour observation with correct surveillance at home or monitored in the ED without systematically using a second CT scan in the absence of clinical signs of brain bleeding […]"

admitted to our ED over 5 consecutive years.“<br><br>(5) Italien				
(1) Cipriano, A., Pecori, A., Bionda, A. E., Bardini, M., Frassi, F., Leoli, F., ... & Santini, M. (2018). Intracranial hemorrhage in anticoagulated patients with mild traumatic brain injury: significant differences between direct oral anticoagulants and vitamin K antagonists. Internal and Emergency Medicine, 13, 1077-1087.<br><br>(2) prospective single-center cohort study<br><br>(3) LoE 3<br><br>(4) „[…]to evaluate the effectiveness and safety of a clinical management protocol according to which patients with MTBI on OAT undergo a CT scan at presentation to the emergency department (ED)	<u>Inclusion:</u> >18y., mTBI (blunt head injury with GCS 13-15), on OAT, single patient visit at the ED for trauma<br><br><u>Exclusion:</u> presentation >48h. after trauma, ineffective OAT (inadequate VKA intake > 1 week before trauma or last dose of DOAC > 24h.), INR < 1.5<br><br>Exclusion to long-term follow-up: immediate traumatic ICH at initial cCT	<u>N=</u> 206<br><br><u>Study site:</u> ED of a level 2 trauma center<br>Hospital protocol: MTBI on OAT receive inital cCT + RHCT after 24 h. of observation (neurological exam every 4-6h.). GCS 15 and asymptomatic: no RHCT after 24 h. observation<br><br><u>Characteristics:</u><br>$\bar{X}$ age 81.53 ± 8.44y.;<br>40.8% male;<br>91.3% accidental fall/syncope;<br>99.0% GCS 15,<br>1.0% GCS 14;<br>58.7% VKA;<br>41.3% DOAK;<br>4.9% concomitant AP therapy (3.4% ASS, 1.5% CP)	<u>(1) Inzidenz pos. cCT:</u><br>11.2% immediate ICH on initial cCT (95% CI 6.5 – 15.5%);<br>in those with immediate ICH 4.3% mortality;<br>in general population 0.5% mortality<br><br><u>(2) Ergebnisse:</u><br>Sign. higher prevalence of following RF in pat. with immediate ICH:<br>- High-energy impact (RR 3.47, 95% CI 1.56 – 7.75),<br>- Trauma above the clavicles (RR 3.90, 95% CI 1.20 – 12.68),<br>- Clin. Relevant fractures (RR 2.85, 95% CI 1.33 – 6.09)<br>- PTA (RR 2.58, 95% CI 1.07 – 6.21)	<u>(1):</u> MINORS: 22 von 24 Punkten<br><br><u>(2) Author’s conclusion:</u> „In the present study, patients on OAT suffering an MTBI and presenting a negative cranial CT scan at presentation underwent a 24-h clinical follow-up, and, if asymptomatic, were discharged home without being subjected to a second CT scan. This protocol has proven to be safe, with a very<br>low rate of delayed complications.“

and admission for a 24-h period of close neurological observation […]"<br><br>(5) Italien				
(1) Leitner, L., El-Shabrawi, J. H., Bratschitsch, G., Eibinger, N., Klim, S., Leithner, A., & Puchwein, P. (2021). Risk adapted diagnostics and hospitalization following mild traumatic brain injury. Archives of orthopaedic and trauma surgery, 141, 619-627.<br><br>(2) retrospective single-center cohort study<br><br>(3) LoE 3<br><br>(4) „Study aim was the analysis of representative subgroups, their risk factors predicting ICH, CT progression and mortality, to allow (1) improved risk estimation on hospitalized mild TBI patients and (2) reduction of unnecessary diagnostics and	Inclusion: >18y. mTBI (GCS 13-15) indicated for cCT and/or hospitalization<br><br>Exclusion: -	N= 1788<br><br>Study site: Level 1 trauma center<br>Hospital protocol: absolute indication for observation (neurosurgical interventions, unconsciousness, posttraumatic changes in cCT) vs. relative indications (doubt, severe headache, nausea, substance intoxication, unclear domestic care)<br><br>Characteristics:<br>X̅ age 58y. (±22.7y., 44.5% >65y.);<br>55.7% male;<br>44.2% RHCT	(1) Inzidenz pos. cCT:<br>- >65y.: 63.7% ICH on initial cCT; 8.5% mortality in those with ICH<br>- 28.3% of those with RHCT had progredient bleeding; 6.0% neurosurgical interventions, 2.5% of all cases died within 30 days<br><br>(2) Ergebnisse:<br>- Pat. were hospitalized für 8.5 ± 10.5 d.<br>- mTBI pat. >65y. without ICH were hospitalized 6.2d ± 8.2d., cases with ICH sign. longer (7.9 ± 6.2 d., p = 0.002)	(1): MINORS: 20 von 24 Punkten<br><br>(2) Author's Conclusion:<br>„Evidence, retrieved from mild TBI cases treated at our Level I Trauma Center within a decade, allowed the identification of risk factors correlated with complications and mortality, enabling more risk-adjusted hospitalization and diagnostics."

hospitalization in low-risk cases."<br><br>(5) Österreich				
(1) Macedo, M., Grima, J., Yangouyian, M., & Kim, B. S. (2017). Delayed Intracranial Hemorrhage in Patients Taking Warfarin with Head Trauma. Spartan Medical Research Journal, 1(2).<br><br>(2) prospective single-center cohort study<br><br>(3) LoE 3<br><br>(4) „[…] The primary goal of the study was to evaluate the utility of a 24-hour observation protocol for potential complications from the fall that could be attributed to a delayed intracranial bleed."<br><br>(5) USA	<u>Inclusion:</u> ≥18y., MHT, taking Warfarin, ED after ground level fall<br><br><u>Exclusion:</u> <18y., not taking Warfarin	<u>N=</u> 51<br><br><u>Study site:</u> ED of a community hospital (annual ED census of 100,000 visits) Hospital protocol: AC pat. with isolated HI and no other reason for admission were admitted to overnight observation unit for 24h. and RHCT<br><br><u>Characteristics:</u><br>median age 71.8y. (range 47-98y.);<br>41% male;<br>94% GCS 15, 4% GCS 14, 2% GCS 13;<br>41% 24h. observation,<br>29% discharged;<br>27% admitted to hospital	<u>(1) Inzidenz pos. cCT:</u><br>- 4% positive initial cCT (no progression on RHCT)<br>- No delayed ICH on RHCT<br><br><u>(2) Ergebnisse:</u><br>38% pat. had RHCT; 2 pat. with initial bleeding (11% of those with RHCT) had no progression	(1): MINORS: 11 von 16 Punkten<br><br><u>(2) Author's conclusion:</u><br>„Given the uncertain nature and timeline of delayed intracranial bleeds, these results indicate that effective patient and family education may be of far greater utility than costly 24-hour observation protocols. The study findings support previous works demonstrating that ongoing observations and costly repeat imaging are unwarranted in many cases and ultimately consume valuable hospital resources."
(1) Marques, R. S. F., Antunes, C., Machado, M. J., Ramos, R., Duarte, N.,	<u>Inclusion:</u> ≥18y., anticoagulated, head trauma, initial cCT neg.	<u>N=</u> 201	<u>(1) Inzidenz pos. cCT:</u>	<u>(1):</u> MINORS: 10 von 16 Punkten

Oliveira, L., ... & Sousa, N. (2021). Reappraising the need for a control CT in mild head injury patients on anticoagulation. European Journal of Trauma and Emergency Surgery, 47, 1461-1466.<br><br>(2) retrospective single-center case series<br><br>(3) LoE 4<br><br>(4) „[…] We also access the need for short-term hospitalization in this group of patients, and based on evidence gathered, we propose a reappraisal of the procedure algorithm."<br><br>(5) Portugal	 Exclusion: <18y., initial cCT pos., INR < 1.2 in case of warfarin or acenocoumarol	Study site: university hospital, first cCT around 4h. after trauma (RHCT around 24h. after trauma)<br><br>Characteristics:<br>X̅ age 81.6y.;<br>43.3% male;<br>99% low energy injury mechanism;<br>57.5% warfarin,<br>38% DOAC	- 1.67% delayed ICH on RHCT (no surgical intervention, no death)<br><br>(2) Ergebnisse:<br>algorithm proposed for RHCT (und damit Observation) (keine Information, wie er erstellt wurde)	(2) Author's conclusion: „[…] we propose that, with certain exceptions, such patients may be discharged after the first CT without intracranial hemorrhage providing that they are accompanied by a caregiver and properly informed about red flags."
(1) Menditto, V. G., Lucci, M., Polonara, S., Pomponio, G., & Gabrielli, A. (2012). Management of minor head injury in patients receiving oral anticoagulant therapy: a prospective study of a 24-hour observation protocol.	Inclusion: ≥14y., mTBI (GCS 14-15), presenting within 48h. after trauma, taking warfarin for at least 1 w., ISS <15<br><br>Exclusion: pos. initial cCT	N= 87<br><br>Study site: Level 2 trauma center (annual ED census of 57,000)<br>Guideline: AC pat. with mTBI receive initial cCT, 24h. observation (EDOU;	(1) Inzidenz pos. cCT:<br>- 16% pos. initial cCT<br>- 6% (95% CI 1%-11%; n=5) had delayed ICH; 3 admitted, 1 had craniotomy<br>(2) Ergebnisse:	(1): MINORS: 12 von 16 Punkten<br><br>(2) Author's conclusion: „In conclusion, our data support the general effectiveness of the European Federation of Neurological Societies'

Annals of emergency medicine, 59(6), 451-455.<br><br>(2) prospective single-center case series<br><br>(3) LoE 4<br><br>(4) „We evaluated the incidence of delayed intracranial bleeding in anticoagulated patients after a minor head injury who were treated according to European guidelines in our emergency department (ED) observational unit.“<br><br>(5) Italien		neurologic exams every 4 – 6h.) and then RHCT<br><br><u>Characteristics:</u><br>median age 82y. (range 60-93; SD 9);<br>32% male;<br>100% GCS 15,<br>21% syncope,<br>79% accidental trauma	- Median time trauma to cCT: 4.5h.; trauma and RHCT 24.5h.<br>- 1 pat. with delayed ICH showed neurologic deterioration<br>- 2 pat. with neg. RHCT readmitted after 2 and 8 d., had subdural hematoma; no neurosurgery<br>Most predicitive of delayed ICH: INR ≥ 3 (RR 14, 95% CI 4 – 49)	recommendations for 24-hour observation followed by a repeated head CT scan for anticoagulated patients with a minor head injury. This protocol will identify most occurrences of delayed bleeding. An initial international normalized ratio greater than 3 suggests higher risk.“
(1) Schoonman, G. G., Bakker, D. P., & Jellema, K. (2014). Low risk of late intracranial complications in mild traumatic brain injury patients using oral anticoagulation after an initial normal brain computed tomography scan: education instead of hospitalization. European	<u>Inclusion:</u> mTBI (GCS 13-15), LOC < 60min, PTA <4h., at least 1 week of VKA, INR > 1.1, cCT within 12h. of trauma<br><br><u>Exclusion:</u> missing data, any traumatic radiological abnormality on initial cCT	<u>N</u>= 211<br><br><u>Study site:</u> ED of a large level 1 trauma hospital<br>Hospital protocol: VKA pat. with mTBI must be admitted für 24h. observation<br><br><u>Characteristics:</u><br>$\bar{X}$ age 77y. (SD 11.7);<br>45.97% male;<br>76.78% hospitalized;	<u>(1) Inzidenz pos. cCT:</u><br>2.37% secondary deterioration (n=5; 1 neurosurgical intervention, 1 death)<br><br><u>(2) Ergebnisse:</u><br>- 1 pat. deteriorated clinically within 24h. after trauma	<u>(1):</u> MINORS: 12 von 16 Punkten<br><br><u>(2) Author’s conclusion:</u> „In conclusion, a low risk of secondary deterioration within 24 h in mTBI patients taking OAC with a normal first head CT scan was found. Our study does not support the recommendation of the

journal of neurology, 21(7), 1021-1025.<br><br>(2) retrospective single-center case study<br><br>(3) LoE 4<br><br>(4) „[…] to develop a save and affordable management strategy for the mTBI patient using OAC. Therefore data were retrospectively collected on patients with mTBI who used OAC to determine the incidence of secondary clinical deterioration after an initial normal head CT scan.“<br><br>(5) Niederlande		73.46% fall from standing	- 4 pat. deteriorated between 2 – 28d. after trauma<br>- In 3/5 pat. with delayed ICH, the initial cCT was not normal in retrospect<br>Not possible to test for RF, to few pat.	current guidelines that these patients should be clinically observed for at least 24 h.“
(1) Singleton, J. M., Bilello, L. A., Greige, T., Balaji, L., Tibbles, C. D., Edlow, J. A., ... & Rosen, C. L. (2021). Outcomes of a novel ED observation pathway for mild traumatic brain injury and associated intracranial hemorrhage. The American	Inclusion: ≥16y., GCS ≥14, isolated HI with minor pos. cCT<br><br>Exclusion: AC/AP use (except ASS), GCS ≤ 13, skull fracture with ass. ICH, epidural hematoma, midline	N= 138<br><br>Study site: Level 1 trauma center (annual ED census of appr. 57,000)<br>Serial neurological exams by a trained ED nurse at 4h. intervals	(1) Inzidenz pos. cCT:<br>63.8% RHCT mit 6.8% (6/88) hematoma expansion, no neurosurgical intervention<br><br>(2) Ergebnisse:<br>- 81.9% pat. successfully discharged home	(1): MINORS: 19 von 24 Punkten<br><br>(2) Author's conclusion: „[…] Based on these results, cmTBI patients with normal neurologic exams, GCS ≥ 14, and without extracranial traumatic injuries or other

Journal of Emergency Medicine, 45, 340-344.<br><br>(2) prospective single-center cohort study<br><br>(3) LoE 3<br><br>(4) „[…] we sought to create an ED-based observation protocol for patients with cmTBI. We hypothesized that ED-based observation would be a safe and efficient disposition for appropriately selected patients."<br><br>(5) USA	shift or mass effect, multiple traumatic injuries	<u>Characteristics:</u><br>$\overline{X}$ age 64.6y. (median 67, SD ± 20.7);<br>53.6% male;<br>80.4% transferred from outside hospitals for neurosurgical evaluation;<br>71.7% falls;<br>63.8% RHCT;<br>42% ASS	- Correlated with pathway non-completion requiring admission: inreased age (median age 79 vs. 66, p=0.02), ASS (p=0.04) but not correlated with admission due to ICH<br>18% admitted, 2.8% specifically for concerns related to ICH (no neurosurgical intervention)	social or acute medical issues requiring inpatient management may be safely discharged from the ED after a brief period of observation."
(1) Yun, B. J., Borczuk, P., Wang, L., Dorner, S., White, B. A., & Raja, A. S. (2018). Evaluation of a low-risk mild traumatic brain injury and intracranial hemorrhage emergency department observation protocol. Academic emergency medicine, 25(7), 769-775.	<u>Inclusion:</u> ≥18y., mTBI (GCS 13-15), acute traumatic ICH<br><br><u>Exclusion:</u> alcohol withdrawal risk, psychiatric instability, spontaneous ICH, additional traumatic injuries requiering admission, medically complex needs, epidural hematoma, cerebellar hemorrhage, SDH ≥ 1cm, IPH with mass effect	<u>N=</u> 296<br><br><u>Study site:</u> urban 999-bed quarternary care academic Level 1 trauma center (approx. 111,000 annual visits), ED with EDOU capacity of 32 beds (staffed with nurse practitioners, PAs, emergency physician supervision)	<u>(1) Inzidenz pos. cCT:</u><br>-<br><br><u>(2) Ergebnisse:</u><br>EDOU-protocol was ass. with an independently stat. sign. decreased aOR for admission or worsening ICH on RHCT (aOR 0.45, 95% CI 0.25 – 0.82)	<u>(1):</u> MINORS: 17 von 24 Punkten<br><br><u>(2) Author's conclusion:</u><br>„After implementing a low risk mild traumatic brain injury and intracranial hemorrhage ED observation protocol, there was a decrease in the rate of inpatient admissions and worsening ICH on repeat CT

(2) retrospective single-center cross-sectional study<br><br>(3) LoE 3<br><br>(4) „Therefore, the purpose of this study was to evaluate outcomes pre- and post-implementation of a low risk traumatic ICH observation protocol in an academic ED/EDOU setting.“<br><br>(5) USA	or midline shift, depressed shull fracture, posttraumatic seizure, anticoagulation, platelet count < 100K/µl	Included pat. were placed in EDOU with neurosurgical consultation<br><br><u>Characteristics:</u><br>Pre-Protocol (n=143):<br>$\bar{X}$ age 73y. (SD 17.73);<br>62% male;<br>93% GCS 15, 3% GCS 14, 2% GCS 13;<br>52.4% SDH <1 cm, 46.2% SAH, 17.5% IPH<br><br>Post-protocol (n=153):<br>$\bar{X}$ age 74 (SD 16);<br>68% male;<br>97% GCS 15, 3% GCS 14;<br>53.6% SAH, 43.1% SDH < 1cm	- Admission rate: pre-protocol 26% vs. post-protocol 13%<br>- RHCT rate: pre-protocol 86% vs. post-protocol 62%<br><br>EDOU-Protocoll:<br>- Consider continuous telemetry for 6-24h.<br>- Neurologic reassessment every 2h. for 6h., then every 4h. (deterioriation: RHCT, page physician)<br>- Physical therapy consultation<br>- Occupational therapy and case management consultation if needed<br>- Discharge after 24h. if stable	from the EDOU. While there was no difference in EDOU LOS, a protocol may help reduce variations in indication and provide physicians guidance on the appropriate type of patients to place in the observation unit.“

Legende: CT = computed tomography; LoE = level of evidence; HCT = head computed tomography; ACAP = anticoagulation and/or antiplatelet therapy; d. = days; RHCT = repeated head computed tomography; cCT = cranial computed tomography; neg. = negative; h. = hours; y. = years; SD = standard deviation; GCS = Glasgow Coma Scale; ICH = intracranial hemorrhage; pos. = positive; TBI = traumatic brain injury; INR = international normalized ratio; ED = emergency department; HI = head injury; LOC = loss of consciousness; m. = minutes; PTA = posttraumatic amnesia; AC = anticoagulation; NMH = niedermolekulare Heparine; DOAK = neue orale Antikoagulantien; AP = antiplatelet therapy; EDOU = emergency department observation unit; OAC = orale Antikoagulation; CTH = head computed tomography; ICH-d = delayed intracranial hemorrhage; NOACs = neue orale Antikoagulantien; SE = standard error; OR = odd's ratio; ISS = injury severity score; CI = confidence interval; MTBI = mild traumatic brain injury; IQR = interquartile range; MHI = mild head injury; sign. = significantly; SAB = Subarachnoidalblutung; SDH = Subduralblutung; FND = focal neurological deficit; FFP = fresh frozen plasma; w. = week; ASS = Acetylsalicylsäure; cmTBI = complicated mild traumatic brain injury

Sonstige Publikationen

(1) Reference, (2) study type, (3) OCEMB Level of Evidence, (4) study aim, (5) country	Inclusion criteria, exclusion criteria	Study population	Results	Author's conclusion
(1) Goldschlager, T., Rosenfeld, J. V., & Winter, C. D. (2007). 'Talk and die'patients presenting to a major trauma centre over a 10 year period: a critical review. Journal of clinical neuroscience, 14(7), 618-623.<br><br>(2) retrospective single-center case series<br><br>(3) LoE 4<br><br>(4) „We have examined the medical records of the adult patients who 'talked and died' over a 10-year period presenting to a major teaching hospital with a well developed trauma system. The evaluation of all aspects of patient demographics and management permits the	Inclusion: >16y., death directly attributable to HI, MHI (GCS 13-15)<br><br>Exclusion: -	N= 15<br><br>Study site: major trauma center<br><br>Characteristics:<br>$\overline{X}$ age 61y.,<br>53% ≥ 70y.;<br>73.33% male;<br>40% minor falls;	(1) Inzidenz pos. cCT:<br>-<br><br>(2) Ergebnisse:<br>- 2.6% of pat. who died as a result of HI had initial MHI (GCS 13-15)<br>- Lucid interval 30 min. – 12h.<br>- 46.67% acute SDH<br><br>RF that may have contributed to deterioriation:<br>- Delay in initial cCT<br>- Lack of early communication/transfer to neurosurgery<br>- Premature discharge<br>- Lack of timely correction of thrombocytopenia and coagulopathy	Author's conclusion: „The incidence of 'talk and die' patients presenting to a major Australasian trauma centre between January 1994 and December 2003 following a mild head injury is 2.6%. […] identify certain factors that may have been contributory to the patients' deterioration. Such instances include delays in obtaining a CT, lack of early communication and transfer to a neurosurgical department, discharges that were premature and the lack of timely correction of thrombocytopenia and coagulopathy.“

identification of potentially preventable factors that may have been contributory to the patients' deterioration."<br><br>(5) Australien				
(1) Techar, K., Nguyen, A., Lorenzo, R. M., Yang, S., Thielen, B., Cain-Nielsen, A., ... & Tignanelli, C. J. (2019). Early imaging associated with improved survival in older patients with mild traumatic brain injuries. Journal of surgical research, 242, 4-10.<br><br>(2) retrospective multicenter cross-sectional study<br><br>(3) LoE 3<br><br>(4) „Therefore, the goal of this study was to identify the optimal time to imaging and the impact of delayed imaging on clinical outcomes for older (age > 50) patients presenting to the emergency department (ED) […] with a mild (GCS > 13) TBI."	<u>Inclusion:</u> ≥50y., versch. ICD-9/ICD-10 codes, GCS ≥ 14, AIS head ≥ 1, cCT within 1.5h. of arrival<br><br><u>Exclusion:</u> direct admission, cCT within 5 min., full trauma activation, ISS > 20	<u>N=</u> 6336<br><br><u>Study site:</u> 33 level 1 and 2 trauma centers<br><br><u>Characteristics:</u><br>Early cCT (≤ 35 min., n=2535):<br>median age 64.3y. (IQR 55.0, 78.0);<br>62.33% male;<br>79.5% GCS 15,<br>20.5% GCS 14;<br>10.9% AC therapy;<br>38.0% AP therapy<br><br>Late cCT (> 35min., n=3801):<br>median age 71.0 (IQR 58.7, 83.0);<br>54% male;<br>82.6%<br>GCS 15, 17.4%<br>GCS 14;<br>10.9% AC therapy;<br>44.1% AP therapy	<u>(1) Inzidenz pos. cCT:</u><br>- 6.06% received RHCT<br>- 0.92% neurosurgical interventions (8.6% in those w/ RHCT)<br><br><u>(2) Ergebnisse:</u><br>- Mortality nadired at 35 min. to cCT<br>- Each 1-min. delay in time to cCT from admission was ass. w/ 2% increase in mortality (OR 1.02, 95% CI 1.01 – 1.03)<br><br>Early (≤35 min.) vs. late (>35 min.) cCT<br>- All-cause in-hospital mortality (OR 0.58, 95% CI 0.35 – 0.95)<br>- FFP in 4h. in AC pat. (OR 1.5, 95% CI 1.04 – 2.2)<br>- ED length of stay (OR 0.9, 95% CI 0.87 – 0.92)	<u>Author's conclusion:</u> „Each 1-min delay in head CT was associated with a 2% increase in mortality, potentially from slower delivery of therapeutic interventions. Head CT within 35 min should be evaluated as a quality metric for older patients with mild TBI."

(5) USA				
(1) Moyer, J. A., Shah, J., Nowakowski, K., Martin, A., McNicholas, A., Muller, A., ... & Ong, A. W. (2018). Does Antithrombotic Drug Use Mandate Trauma Team Activation in Awake Geriatric Patients with Intracranial Hemorrhage?. The American Surgeon, 84(7), 1180-1184.<br><br>(2) retrospective single-center cross-sectional study<br><br>(3) LoE 3<br><br>(4) „Although antithrombotic drug use has accordingly been incorporated into the triage criteria for TTA at our institution, it is not clear whether TTA in awake geriatric patients with intracranial hemorrhage (ICH) on antithrombotic agents is associated with improved outcomes. We hypothesized that for this cohort, TTA results in expeditious evaluation and	<u>Inclusion:</u> radiographically confirmed ICH after fall, AIS 2, ≥65y., GCS ≥ 13<br><br><u>Exclusion:</u> subacute or chronic ICH, transferred from another facility without accompanying cCT images	<u>N=</u> 419<br><br><u>Study site:</u> level 1 trauma center of a teaching hospital TTA criteria: GCS ≤ 13 regardless of AC/AP therapy or GCS ≥ 14 w/ evidence of TBI (cephalohematoma and LOC) and on AC/AP therapy<br><br><u>Characteristics:</u><br>median age 82y. (IQR 75 - 88);<br>20.5% group 1 (AC with or without AP, n=86):<br>54% male,<br>median GCS 15;<br>39.5% TTA<br><br>50.4% group 2 (only AP, n=211):<br>53.08% male;<br>median GCS 15;<br>18.01% TTA<br><br>29.1% group 3 (no AC/AP, n=122):<br>41% male;	<u>(1) Inzidenz pos. cCT:</u><br>Mortality:<br>- Group 1: 12.79%<br>- Group 2: 11.37%<br>- Group 3: 10.66%<br><br>Neurosurgical interventions:<br>- Group 1: 4.65%<br>- Group 2: 4.74%<br>- Group 3: 4.92%<br><br><u>(2) Ergebnisse:</u><br>- AC/AP therapy and TTA status were not independently ass. w/ any of the outcomes<br>- TTA pat. had shorter time to cCT (median 20 min., IQR 13-33 vs. median 80 min., IQR 56 – 131; p<0.0001)	<u>Author's conclusion:</u> „In elderly patients with GCS ≥13 on antithrombotic drugs found to have ICH, TTA facilitated expeditious evaluation and treatment, with shorter times to CT and initiation of reversal agents, compared with no TTA. However, this was not associated with reduced mortality or need for neurosurgical intervention within 48 hours of admission."

treatment and is thus associated with mortality benefit."<br><br>(5) USA		median GCS 14 in TTA, median GCS 15 in non-TTA; 31.97% TTA		
(1) Cheng, P. L., Lin, H. Y., Lee, Y. K., Hsu, C. Y., Lee, C. C., & Su, Y. C. (2014). Higher mortality rates among the elderly with mild traumatic brain injury: a nationwide cohort study. Scandinavian journal of trauma, resuscitation and emergency medicine, 22(1), 1-7.<br><br>(2) prospective multicenter cohort study<br><br>(3) LoE 3<br><br>(4) „Our study aimed to investigate the correlation between mild TBI and death in the elderly by utilizing a large administration database to overcome the obstacles noted above."<br><br>(5) Taiwan	Inclusion: cohort: alive in 2005, >65y., had mTBI (ICD-9 codes)<br><br>Exclusion: diagnosis of mTBI before January 1, 2005; hospitalized for TBI	N= 90114<br><br>Study site: -<br><br>Characteristics:<br>mTBI group (n=5997):<br>$\bar{X}$ age 75.8y. (SD 7.3);<br>47.5% male;<br>65.1% low socioeconomic status<br><br>control group (n=84117):<br>$\bar{X}$ age 73.6y. (SD 6.5);<br>49.9% male;<br>67.3% low socioeconomic status	(1) Inzidenz pos. cCT:<br>-<br><br>(2) Ergebnisse:<br>- mTBI pat. were sign. older, sign. more likely to have diabetes, hypertension, history of alcohol intox., ischemic stroke, ICH, dementia, higher CCI score<br>- average duration from TBI to death 1.40y. (95% CI 1.32 – 1.48)<br>- crude HR of death between the groups was 1.51 (95% CI 1.42 – 1.62)	Author's conclusion: „Mild TBI is an independent significant risk factor of death<br>in the elderly. The result indicates that more emphasis on head injury prevention would be worthwhile."

Legende: LoE = Level of Evidence, y. =years, HI = head injury, MHI = mild head injury, GCS = Glasgow Coma Scale, $\overline{X}$ = mean, pos. = positive, cCT = cranial Computed Tomography, pat. = patient, min. = minute, h. = hour, SDH = subdural hematoma, RF = risk factor, ISS = injury severity score, AC = anticoagulation, AP = antiplatelet, RHCT = repeat head Computed tomography, w/ = with, ass. = associated, OR = odd's ratio, CI = confidence interval, FFP = fresh frozen plasma, ED = emergency department, ICH = intracranial hemorrhage, TTA = trauma team activation, AIS = abbreviated injury scale, TBI = traumatic brain injury, IQR = interquartile range, mTBI = mild traumatic brain injury, SD = standard deviation, sign. = significantly, HR = hazard ratio
