# Supplementary material for: Diagnostic algorithm for the management of patients ≥ 65 years with a mild traumatic brain injury in the emergency department
Source: Unfallchirurgie (Heidelb). 2026 Aug 3;129(10):696–708. [Article in German] doi: 10.1007/s00113-026-01737-4 (PMC13601116; doi:10.1007/s00113-026-01737-4)
Supplement: Supplementary file 3 [file 113_2026_1737_MOESM3_ESM.pdf]

Appendix 3- Bewertungsschema Evidenz

Stufe 1: Starke Evidenz	Konsistente Ergebnisse (protektiver Faktor oder Risikofaktor) in ≥ 2 eingeschlossenen Studien mit suffizienter Berichterstattung
Stufe 2: Moderate Evidenz	Konsistente Ergebnisse (protektiver Faktor oder Risikofaktor) in einer Studie mit suffizienter Berichterstattung und ≥ 1 Studie mit moderater Berichterstattung
Stufe 3: Limitierte Evidenz	eine Studie mit suffizienter Berichterstattung unabhängig der Stichprobengröße / Konsistente Ergebnisse (protektiver Faktor oder Risikofaktor) in > 1 Studie mit moderater/insuffizienter Berichterstattung
Stufe 4: Inkonsistente Evidenz	Inkonsistente Ergebnisse unabhängig von der Berichterstattung

Tabelle 3, Legende: SE = starke Evidenz; ME = moderate Evidenz; LE = limitierte Evidenz; IE = inkonsistente Evidenz
