# Supplementary material for: Diagnostic algorithm for the management of patients ≥ 65 years with a mild traumatic brain injury in the emergency department
Source: Unfallchirurgie (Heidelb). 2026 Aug 3;129(10):696–708. [Article in German] doi: 10.1007/s00113-026-01737-4 (PMC13601116; doi:10.1007/s00113-026-01737-4)
Supplement: Supplementary file 4 [file 113_2026_1737_MOESM4_ESM.pdf]

Appendix 4- Regressionsanalysen Primärstudien

Tabellenaufbau:

* = p< 0.05; ** = p < 0.001; *** = p < 0.0001

NICE = NICE Head injury: assessment and early management; CCHR = Canadian CT Head Rule; NEXUS II = National Emergency X-Radiography Utilization Study II; NOC = New Orleans Criteria

1) Brown, A. J., Witham, M. D., & George, J. (2011). Development of a risk score to guide brain imaging in older patients admitted with falls and confusion. The British Journal of Radiology, 84(1004), 756-757.

Risikofaktor	Definition	Statistisch signifikant in multivariater Regressionsanalyse assoziiert mit pathologischer cCT	Statistisch signifikant in univariater Regressionsanalyse assoziiert mit pathologischer cCT	Nicht statistisch signifikant	Risikofaktor in NICE, CCHR, NEXUS II, NOC enthalten ?
Atrial fibrillation	-	Nicht berichtet		x	
History of falls	-	Nicht berichtet		x	
Face/scalp trauma	-	Nicht berichtet		x	NOC, NEXUS II
New focal neurological signs	-	Nicht berichtet		x	NEXUS II, NICE,
Warfarin	-	Nicht berichtet		x	NICE
Dementia	-	Nicht berichtet		x	
GCS < 14	-	Nicht berichtet		x	NEXUS II, NICE, CCHR
INR above therapeutic range	-	Nicht berichtet		x	NEXUS II, NICE
History of alcohol abuse	-	Nicht berichtet		x	NOC
Vomiting	-	Nicht berichtet		x	NEXUS II, NOC, NICE, CCHR
Headache	-	Nicht berichtet		x	NOC
Previous stroke	-	Nicht berichtet		x	

Legende: cCT = cranial computed tomography; GCS = Glasgow Coma Scale; INR = international normalized ratio; * = p< 0.05; ** = p < 0.001; *** = p < 0.0001; NICE = NICE Head injury: assessment and early management; CCHR = Canadian CT-Head Rule; NEXUS II = National Emergency X-Radiography Utilization Study II; NOC = New Orleans Criteria

2) Lee, Y. B., & Kwon, S. J. (2009). A more detailed classification of mild head injury in adults and treatment guidelines. Journal of Korean Neurosurgical Society, 46(5), 451-458.

Risikofaktor	Definition	Statistisch signifikant in multivariater Regressionsanalyse assoziiert mit pathologischer cCT	Statistisch signifikant in univariater Regressionsanalyse assoziiert mit pathologischer cCT	Nicht statistisch signifikant	CCHR, NEXUS II, NOC, NICE
Male	-		***		
Age ≥ 65	-	OR 6.40 (95% CI 3.81 – 10.75); ***	***		NICE, NOC, NEXUS II, CCHR
GCS 15	Initial GCS at admission			x	
GCS 14	Initial GCS at admission	OR 4.50 (95% CI 2.47 – 8.19); ***	***		NEXUS II
GCS 13	Initial GCS at admission	OR 11.10 (95% CI 4.22 – 29.25), ***	***		NICE
LOC	Less than 20 minutes	OR 1.84 (95% CI 1.24 - 2.71); ***	***		
Nausea/Vomiting	-			x	NICE, CCHR, NOC, NEXUS II
Dizziness	-			x	
Amnesia	-			x	NICE, CCHR, NOC
Neurological deficit	-		***		NICE, NEXUS II
Coagulopathy	-		***		NICE, NEXUS II
Alcohol consumption	-		***		NOC
Signs of basal skull fracture	-		***		NICE, CCHR
Skull fracture	-	OR 25.50 (95% CI 12.92 – 50.18); ***	***		NICE, CCHR, NEXUS II

Legende: cCT = cranial computed tomography; GCS = Glasgow Coma Scale; OR = Odd's Ratio; CI = confidence interval; LOC = loss of consciousness; * = p< 0.05; ** = p < 0.001; *** = p < 0.0001; NICE = NICE Head injury: assessment and early management; CCHR = Canadian CT-Head Rule; NEXUS II = National Emergency X-Radiography Utilization Study II; NOC = New Orleans Criteria

3) Fournier, N., Gariepy, C., Prévost, J. F., Belhumeur, V., Fortier, É., Carmichael, P. H., ... & Émond, M. (2019). Adapting the Canadian CT head rule age criteria for mild traumatic brain injury. Emergency Medicine Journal, 36(10), 617-619.

Keine Regressionsanalyse durchgeführt

4) O'Brien, T., Mitra, B., Le Sage, N., Tardif, P. A., Emond, M., D'Astous, M., & Mercier, E. (2020). Clinically significant traumatic intracranial hemorrhage following minor head trauma in older adults: a retrospective cohort study. Brain injury, 34(6), 836-841.

Keine Daten der Regressionsanalyse berichtet

5) Pages, P. J., Boncoeur-Martel, M. P., Dalmay, F., Salle, H., Caire, F., Mounayer, C., & Rouchaud, A. (2020). Relevance of emergency head CT scan for fall in the elderly person. Journal of Neuroradiology, 47(1), 54-58.

Risikofaktor	Definition	Statistisch signifikant in multivariater Regressionsanalyse assoziiert mit pathologischer cCT	Statistisch signifikant in univariater Regressionsanalyse assoziiert mit pathologischer cCT	Nicht statistisch signifikant	CCHR, NEXUS II, NOC, NICE
Male sex	-	RR 2.19 (95% CI 1.12 – 4.29); *	Nicht berichtet		
Past history of post-traumatic brain injury	-	RR 7.17 (95% CI 1.98 - 26.05); *	Nicht berichtet		
Consciousness impairment	Decrease in GCS	RR 1.56 (95% CI 1.28 – 1.97); ***	Nicht berichtet		NICE, CCHR, NEXUS II
Focal neurological deficit	At admission	RR 6.36 (95% CI 1.13 – 35.92); *	Nicht berichtet		NICE, NEXUS II
Anticoagulation medication	-			x	NICE
Disturbance of hemostasis	-			x	NICE, NEXUS II

Legende: cCT = cranial computed tomography; RR = relative risk; CI = confidence interval; * = p< 0.05; ** = p < 0.001; *** = p < 0.0001; NICE = NICE Head injury: assessment and early management; CCHR = Canadian CT-Head Rule; NEXUS II = National Emergency X-Radiography Utilization Study II; NOC = New Orleans Criteria

6) Ibañez Pérez De La Blanca, M. A., Fernández Mondéjar, E., Gómez Jimènez, F. J., Alonso Morales, J. M., Lombardo, M. D. Q., & Viso Rodriguez, J. L. (2018). Risk factors for intracranial lesions and mortality in older patients with mild traumatic brain injuries. Brain injury, 32(1), 99-104.

Risikofaktor	Definition	Statistisch signifikant in multivariater Regressionsanalyse assoziiert mit pathologischer cCT	Statistisch signifikant in bivariater Regressionsanalyse assoziiert mit pathologischer cCT	Nicht statistisch signifikant	CCHR, NEXUS II, NOC, NICE
Traffic accident	-	OR 3.052 (95% CI 1.163 – 8.011); *			NICE, CCHR
GCS 14	-	OR 3.942 (95%CI 2.197 – 7.071); **	OR 3.15 (95% CI 1.92 – 5.19); **		NICE, CCHR, NEXUS II

Transient LOC	-	OR 1.985 (95% CI 1.211 – 3.254); **	OR 2.14 (95% CI 1.36 – 3.36); **		
Nausea	-	OR 3.649 (95% CI 2.027 – 6.569); **	OR 3.44 (95% CI 2.02 – 5.84); **		
Vomiting	-		OR 3.22 (95% CI 1.68 – 6.14); **		NICE, NOC, CCHR, NEXUS II
Antiplatelet medication	ASS, Clopidogrel	OR 1.718 (95% CI 1.027 – 2.872); *			NICE
SSRI/Benzodiazepines	-			x	
Focal neurological deficit	-			x	NICE, NEXUS II
Dizziness	-			x	
Headache	-		OR 1.71 (95% CI 1.13 – 2.59); *		NOC
Amnesia	-		OR 2.38 (95% CI 1.45 – 3.90); **		NICE, CCHR
Seizure	-			x	NOC
Cranio-facial lesion	-			x	NOC, NEXUS II
Fall > 1 meter	-			x	
Alcohol intoxication	-			x	NOC
Renal failure	-			x	
Tumor	-			x	
Hypertension	-			x	
Dyslipidemia	-			x	
Chronic neurologic disease	-			x	
Diabetes	-			x	
Thrombocytopenia	-			x	NEXUS II, NICE
Anticoagulation medication	VKA			x	NICE

Legende: cCT = cranial computed tomography; OR = Odd's Ratio; CI = confidence interval; GCS = Glasgow Coma Scale; SSRI = selective serotonine reuptake inhibitor; LOC = loss of consciousness; ASS = Aspirin; * = p< 0.05; ** = p < 0.001; *** = p < 0.0001; NICE = NICE Head injury: assessment and early management; CCHR = Canadian CT-Head Rule; NEXUS II = National Emergency X-Radiography Utilization Study II; NOC = New Orleans Criteria

7) Leitner, L., El-Shabrawi, J. H., Bratschitsch, G., Eibinger, N., Klim, S., Leithner, A., & Puchwein, P. (2021). Risk adapted diagnostics and hospitalization following mild traumatic brain injury. Archives of orthopaedic and trauma surgery, 141, 619-627.

Risikofaktor	Definition	Statistisch signifikant in multivariater Regressionsanalyse assoziiert mit pathologischer cCT	Statistisch signifikant in univariater Regressionsanalyse assoziiert mit pathologischer cCT	Nicht statistisch signifikant	CCHR, NEXUS, NOC, NICE

Age	-	OR 1.01; **	Nicht berichtet		
Anticoagulation medication	-	OR 1.1; **	**		NICE
Neurocranial fracture	-	OR 1.2; **	**		NEXUS II, NICE, CCHR
Female sex	-		*		
Renal insufficiency	-		**		
History of stroke	-		**		
ASS intake	-		**		

Legende: cCT = cranial computed tomography; ASS = Aspirin; OR = Odd's Ratio; * = p< 0.05; ** = p < 0.001; *** = p < 0.0001; NICE = NICE Head injury: assessment and early management; CCHR = Canadian CT-Head Rule; NEXUS II = National Emergency X-Radiography Utilization Study II; NOC = New Orleans Criteria

8) Mori, K., Abe, T., Matsumoto, J., Takahashi, K., & Takeuchi, I. (2021). Indications for Computed Tomography in Older Adult Patients With Minor Head Injury in the Emergency Department. Academic Emergency Medicine, 28(4), 435-443.

Risikofaktor	Definition	Statistisch signifikant in multivariater Regressionsanalyse assoziiert mit pathologischer cCT	Statistisch signifikant in univariater Regressionsanalyse assoziiert mit pathologischer cCT	Nicht statistisch signifikant	CCHR, NEXUS, NOC, NICE
High-risk mechanism of injury		OR 3.03 (95% CI 1.33 – 6.92) OR 3.03 (95% CI 1.33 – 6.92)	OR 2.34 (95% CI 1.32 – 4.13)	-	NICE, CCHR
Vomiting		OR 28.45 (95% CI 6.76 – 119.77)	OR 15.19 (95% CI 5.18 – 44.54)	-	NOC
Witnessed LOC		OR 5.47 (95% CI 1.92 – 15.56)	OR 6.63 (95% CI 3.11 – 14.13)	-	
Anterograde amnesia		OR 3.97 (95% CI 1.15 – 13.62)	OR 8.61 (95% CI 4.12 – 18.02)	-	NOC, CCHR
≥ 75y.		Nicht berichtet		x	NOC, NEXUS II, NICE, CCHR
≥ 80y.		Nicht berichtet		x	NOC, NEXUS II, NICE, CCHR
≥ 85y.		Nicht berichtet		x	NOC, NEXUS II, NICE, CCHR
Female sex		Nicht berichtet		x	
GCS < 15			OR 2.07 (95% CI 1.18 – 3.64)	-	NEXUS II, NICE, CCHR
GCS < 14		Nicht berichtet		x	NEXUS II, NICE, CCHR

Syst. RR > 180		Nicht berichtet		x	
Antiplatelet medication		Nicht berichtet		x	
Chronic renal failure with dialysis		Nicht berichtet		x	
Chronic alcohol abuse		Nicht berichtet		x	NOC
Headache	Pain scale ≥8/10	Nicht berichtet		x	NOC
Retrograde amnesia		-	OR 5.21 (95% CI 2.57 – 10.57)	-	
Distracting injury		Nicht berichtet		x	NOC
Toxic		Nicht berichtet		x	NOC
Hematoma		Nicht berichtet		x	

Legende: cCT = cranial computed tomography; OR = Odd's ratio; CI = confidence interval; LOC = loss of consciousness; y. = years; GCS = Glasgow Coma Scale; Syst. = systolic; RR = Blutdruck, * = p< 0.05; ** = p < 0.001; *** = p < 0.0001; NICE = NICE Head injury: assessment and early management; CCHR = Canadian CT-Head Rule; NEXUS II = National Emergency X-Radiography Utilization Study II; NOC = New Orleans Criteria

9) Yuksen, C., Sittichanbuncha, Y., Patumanond, J., Muengtaweepongsa, S., & Sawanyawisuth, K. (2018). Clinical predictive score of intracranial hemorrhage in mild traumatic brain injury. Therapeutics and clinical risk management, 213-218.

Risikofaktor	Definition	Statistisch signifikant in multivariater Regressionsanalyse assoziiert mit pathologischer cCT	Statistisch signifikant in univariater Regressionsanalyse assoziiert mit pathologischer cCT	Nicht statistisch signifikant	CCHR, NEXUS II, NOC, NICE
Posttraumatic vomiting more than two times	-		**		NEXUS II, NICE, CCHR
Severe headache	Visual analog scale > 7	aOR 99.81 (95% CI 37.38 – 266.50); **	**		NOC
Transient LOC	>15 minutes or witnessed LOC	aOR 5.85 (95% CI 2.92 – 11.71); **	**		
Posttraumatic amnesia	<1h.		**		CCHR, NOC
Focal neurological deficit	-	aOR 10.43 (95% CI 4.97 – 21.89); **	**		NICE, NEXUS II
Clinical signs of skull fracture	-	aOR 34.13 (95% CI 4.37 – 266.53); **	**		NEXUS II, NICE, CCHR
Base of skull fracture	-	aOR 139.38 (95% CI 13.64 – 1424,24); **	**		NICE, CCHR

Legende: cCT = cranial computed tomography; LOC = loss of consciousness; h. = hours; aOR = adjusted Odd's Ratio; CI = confidence interval; * = p< 0.05; ** = p < 0.001; *** = p < 0.0001; NICE = NICE Head injury: assessment and early management; CCHR = Canadian CT-Head Rule; NEXUS II = National Emergency X-Radiography Utilization Study II; NOC = New Orleans Criteria

10) Riccardi, A., Frumento, F., Guiddo, G., Spinola, M. B., Corti, L., Minuto, P., & Lerza, R. (2013). Minor head injury in the elderly at very low risk: a retrospective study of 6 years in an emergency department (ED). The American journal of emergency medicine, 31(1), 37-41.

Keine Regressionsanalyse durchgeführt

11) Mack, L. R., Chan, S. B., Silva, J. C., & Hogan, T. M. (2003). The use of head computed tomography in elderly patients sustaining minor head trauma. The Journal of emergency medicine, 24(2), 157-162.

Risikofaktor	Definition	Statistisch signifikant in multivariater Regressionsanalyse assoziiert mit pathologischer cCT	Statistisch signifikant in univariater Regressionsanalyse assoziiert mit pathologischer cCT	Nicht statistisch signifikant	CCHR, NEXUS, NOC, NICE
Chronic altered mental status	-		*		
Age	-			x	
Male	-		**		
GCS < 15	-			x	NEXUS II, NICE, CCHR
Aspirin	-			x	
Coumadin	-			x	NICE
Ethanol	-			x	NOC
LOC	-		*	-	
Confusion	-			x	
Balance disorder	-			x	
Nausea/emesis	-			x	CCHR, NICE, NOC, NEXUS II
Dizziness	-			x	
Headache	-			x	NOC
Amnesia	-			x	NOC, NICE, CCHR
Contusion or hematoma	-			x	
Laceration /abrasion	-			x	NOC, NEXUS II
Fracture	-			x	NEXUS II, NOC, CCHR
Agitation	-			x	
New altered mental status	-			x	

Legende: cCT = cranial computed tomography; GCS = Glasgow Coma Scale; * = p< 0.05; ** = p < 0.001; *** = p < 0.0001; NICE = NICE Head injury: assessment and early management; CCHR = Canadian CT-Head Rule; NEXUS II = National Emergency X-Radiography Utilization Study II; NOC = New Orleans Criteria

12) Brewer, E. S., Reznikov, B., Liberman, R. F., Baker, R. A., Rosenblatt, M. S., David, C. A., & Flacke, S. (2011). Incidence and predictors of intracranial hemorrhage after minor head trauma in patients taking anticoagulant and antiplatelet medication. Journal of Trauma and Acute Care Surgery, 70(1), E1-E5.

Risikofaktor	Definition	Statistisch signifikant in multivariater Regressionsanalyse assoziiert mit pathologischer cCT	Statistisch signifikant in logistischer Regressionsanalyse assoziiert mit pathologischer cCT	Nicht statistisch signifikant	CCHR, NEXUS, NOC, NICE
LOC	-	Nicht berichtet	*	-	
Gender	-	Nicht berichtet		x	
Clopidogrel	-	Nicht berichtet		x	
Warfarin	-	Nicht berichtet		x	NICE
ASA	-	Nicht berichtet		x	
Mechanism of injury	-	Nicht berichtet		x	NICE
Presence of fracture	-	Nicht berichtet		x	NICE, CCHR, NEXUS II
External evidence of head injury above the clavicles	-	Nicht berichtet		x	NOC
Age	-	Nicht berichtet		x	
INR	-	Nicht berichtet		x	
PTT	-	Nicht berichtet		x	

Legende: cCT = cranial computed tomography; LOC = loss of consciousness; ASA = Aspirin; INR = international normalized ratio; PTT = partielle Thromboplastinzeit; * = $p < 0.05$; ** = $p < 0.001$; *** = $p < 0.0001$; NICE = NICE Head injury: assessment and early management; CCHR = Canadian CT-Head Rule; NEXUS II = National Emergency X-Radiography Utilization Study II; NOC = New Orleans Criteria

13) McCammack, K. C., Sadler, C., Guo, Y., Ramaswamy, R. S., & Farid, N. (2015). Routine repeat head CT may not be indicated in patients on anticoagulant/antiplatelet therapy following mild traumatic brain injury. Western Journal of Emergency Medicine, 16(1), 43.

Risikofaktor	Definition	Statistisch signifikant in multivariater Regressionsanalyse assoziiert mit pathologischer cCT	Statistisch signifikant in univariater Regressionsanalyse assoziiert mit pathologischer cCT	Nicht statistisch signifikant	CCHR, NEXUS, NOC, NICE
Male sex			Nicht berichtet	x	

GCS < 15		**	Nicht berichtet		NEXUS II, NICE, CCHR
LOC		OR 9.44 (95% CI 2.57 – 34.73); *	Nicht berichtet		
Pertinent neurological examination findings		OR 24.62 (95% CI 5.80 – 104.42); **	Nicht berichtet		NICE
Warfarin			Nicht berichtet	x	NICE
Clopidogrel		OR 11.17 (95% CI 2.33 – 53.59); *	Nicht berichtet		
Aspirin		OR 4.74 (95% CI 1.16 – 19.36); *	Nicht berichtet		
Dipyridamole			Nicht berichtet	x	
Dabigatran			Nicht berichtet	x	
Combination therapy		OR 4.11 (95% CI 1.59 – 10.82); *	Nicht berichtet		
INR			Nicht berichtet	x	
Prothrombin time			Nicht berichtet	x	
Partial thromboplastin time			Nicht berichtet	x	
Platelet count			Nicht berichtet	x	

Legende: cCT = cranial computed tomography; GCS = Glasgow Coma Scale; LOC = loss of consciousness; INR = international normalized ratio, OR = Odd's Ratio; CI = confidence interval; * = p< 0.05; ** = p < 0.001; *** = p < 0.0001; NICE = NICE Head injury: assessment and early management; CCHR = Canadian CT-Head Rule; NEXUS II = National Emergency X-Radiography Utilization Study II; NOC = New Orleans Criteria

14) Nishijima, D. K., Offerman, S. R., Ballard, D. W., Vinson, D. R., Chettipally, U. K., Rauchwerger, A. S., ... & Clinical Research in Emergency Services and Treatment (CREST) Network. (2013). Risk of traumatic intracranial hemorrhage in patients with head injury and preinjury warfarin or clopidogrel use. Academic Emergency Medicine, 20(2), 140-145.

Risikofaktor	Definition	Statistisch signifikant in multivariater Regressionsanalyse assoziiert mit pathologischer cCT	Statistisch signifikant in univariater Regressionsanalyse assoziiert mit pathologischer cCT	Nicht statistisch signifikant	CCHR, NEXUS II, NOC, NICE
Vomiting	-	aOR 3.68 (95% CI 1.55 – 8.76)	Nicht berichtet		NOC, NEXUS II, NICE, CCHR
Abnormal mental status	-	aOR 3.08 (95% CI 1.60 – 5.94)	Nicht berichtet		
Clopidogrel	-		Nicht berichtet		
Headache	-		Nicht berichtet		NOC
Drug or alcohol intoxication	-		Nicht berichtet	x	NOC

≥65y.	-		Nicht berichtet	x	NICE, CCHR, NEXUS II, NOC
Warfarin			Nicht berichtet	x	NEXUS II
Concomitant ASS	-		Nicht berichtet	x	
Non-groundlevel fall	-		Nicht berichtet	x	
LOC or amnesia	-		Nicht berichtet	x	NOC, NICE
Trauma above the clavicles	-		Nicht berichtet	x	NOC

Legende: cCT = cranial computed tomography; LOC = loss of consciousness; ASS = Aspirin; aOR = adjusted Odd's Ratio; CI = confidence interval; * = p< 0.05; ** = p < 0.001; *** = p < 0.0001; NICE = NICE Head injury: assessment and early management; CCHR = Canadian CT-Head Rule; NEXUS II = National Emergency X-Radiography Utilization Study II; NOC = New Orleans Criteria

15) Hamden, K., Agresti, D., Jeanmonod, R., Woods, D., Reiter, M., & Jeanmonod, D. (2014). Characteristics of elderly fall patients with baseline mental status: high-risk features for intracranial injury. The American Journal of Emergency Medicine, 32(8), 890-894.

Risikofaktor	Definition	Statistisch signifikant in multivariater Regressionsanalyse assoziiert mit pathologischer cCT	Statistisch signifikant in univariater Regressionsanalyse assoziiert mit pathologischer cCT	Nicht statistisch signifikant	CCHR, NEXUS II, NOC, NICE
LOC	-	OR 2.77 (95% CI 1.23 – 6.27)	Nicht berichtet		
Signs of trauma to head/face	-	OR 13.2 (95% CI 2.7 – 64.1)	Nicht berichtet		NEXUS II
Position at time of injury	-		Nicht berichtet	x	
History of striking the head	-		Nicht berichtet	x	
Complaint of new headache	-		Nicht berichtet	x	NOC
ASS	-		Nicht berichtet	x	
Other antiplatelet use	-		Nicht berichtet	x	
Anticoagulation medication	-		Nicht berichtet	x	NICE
Significant traumatic injuries	-		Nicht berichtet	x	

Legende: cCT = cranial computed tomography; LOC = loss of consciousness; ASS = Aspirin; OR = Odd's Ratio; CI = confidence interval; y. = years; * = p< 0.05; ** = p < 0.001; *** = p < 0.0001; NICE = NICE Head injury: assessment and early management; CCHR = Canadian CT-Head Rule; NEXUS II = National Emergency X-Radiography Utilization Study II; NOC = New Orleans Criteria

16) Fabbri, A., Servadei, F., Marchesini, G., Stein, S. C., & Vandelli, A. (2010). Predicting intracranial lesions by antiplatelet agents in subjects with mild head injury. Journal of Neurology, Neurosurgery & Psychiatry, 81(11), 1275-1279.

Risikofaktor	Definition	Statistisch signifikant in multivariater Regressionsanalyse assoziiert mit pathologischer cCT	Statistisch signifikant in univariater Regressionsanalyse assoziiert mit pathologischer cCT	Nicht statistisch signifikant	CCHR, NEXUS II, NOC, NICE
GCS 14	-	OR 17.8 (95% CI 13.3 – 23.9); **	Nicht berichtet		NICE, CCHR, NEXUS II
Suspected skull fracture	-	OR 13.9 (95% CI 8.9 – 21.7); **	Nicht berichtet		NICE, CCHR, NEXUS II
Neurological deficit	-	OR 115.7 (95% CI 76.9 – 174.1); **	Nicht berichtet		NEXUS II, NICE
LOC	-	OR 3.0 (95% CI 2.3 – 3.9); **	Nicht berichtet		
Amnesia	-		Nicht berichtet	x	NICE, CCHR
Age ≥ 65y.	-		Nicht berichtet	x	NICE, CCHR, NOC, NEXUS II
Coagulopathy	-	OR 7.4 (95% CI 5.0 – 11.1); **	Nicht berichtet		NEXUS II, NICE
Seizure	-	OR 3.4 (95% CI 2.1 – 5.6); **	Nicht berichtet		NOC
Visible trauma	-		Nicht berichtet	x	
Headache	-		Nicht berichtet	x	NOC
Injury mechanism	-		Nicht berichtet	x	
Intoxication	-		Nicht berichtet	x	NOC
Previous neurosurgery	-		Nicht berichtet	x	
Antiplatelet drugs	-	OR 2.8 (95% CI 2.0 – 3.9); **	Nicht berichtet		
Vomiting	-	OR 17.1 (95% CI 12.0 – 24.4); **	Nicht berichtet		NICE, NOC, NEXUS II, CCHR

Legende: cCT = cranial computed tomography; y. = years; GCS = Glasgow Coma Scale; LOC = loss of consciousness; OR = Odd's Ratio; CI = confidence interval; y. = years; * = p< 0.05; ** = p < 0.001; *** = p <

0.0001; NICE = NICE Head injury: assessment and early management; CCHR = Canadian CT-Head Rule; NEXUS II = National Emergency X-Radiography Utilization Study II; NOC = New Orleans Criteria

17) Teeratakulpisarn, P., Angkasith, P., Wannakul, T., Tanmit, P., Prasertcharoensuk, S., Thanapaisal, C., ... & Sawanyawisuth, K. (2021). What are the strongest indicators of intracerebral hemorrhage in mild traumatic brain injury?. Trauma Surgery & Acute Care Open, 6(1), e000717.

Risikofaktor	Definition	Statistisch signifikant in multivariater Regressionsanalyse assoziiert mit pathologischer cCT	Statistisch signifikant in univariater Regressionsanalyse assoziiert mit pathologischer cCT	Nicht statistisch signifikant	CCHR, NEXUS II, NOC, NICE
GCS 13-14 after 2h. of observation	-	aOR 18.10 (95% CI 1.91 – 171.29)	OR 19.73 (95% CI 2.17 – 179.06)		NICE, CCHR
Open skull or base of skull fracture	-	aOR 8.24 (95% CI 1.33 – 50.75)	OR 9.73 (95% CI 1.75 – 54.13)		NEXUS II, NICE, CCHR
Vomiting > 2 times	-			Nicht berichtet	NOC, NEXUS II, NICE, CCHR
FND	-			Nicht berichtet	NEXUS II, NICE
Post-traumatic seizure	-			Nicht berichtet	NOC
≥60y.	-			Nicht berichtet	NOC
High-energy trauma	-			Nicht berichtet	CCHR, NICE
Motor vehicle crash	-		OR 9.22 (95% CI 3.23 – 26.31)		CCHR, NICE
Multiple injuries	-		OR 2.95 (95% CI 1.02 -8.54)		

Legende: cCT = cranial computed tomography; GCS = Glasgow Coma Scale; h. = hours; FND = focal neurological deficit; y. = years; OR = Odd's Ratio; aOR = adjusted Odd's Ratio; CI = confidence intervals; * = p< 0.05; ** = p < 0.001; *** = p < 0.0001; NICE = NICE Head injury: assessment and early management; CCHR = Canadian CT-Head Rule; NEXUS II = National Emergency X-Radiography Utilization Study II; NOC = New Orleans Criteria

18) Colas, L., Graf, S., Ding, J., Bertolotti, G., Thellier, N., Budzik, J. F., & Verclytte, S. (2021). Limited benefit of systematic head CT for mild traumatic brain injury in patients under antithrombotic therapy. Journal of Neuroradiology.

Keine Regressionsanalyse durchgeführt

19) Savioli, G., Ceresa, I. F., Ciceri, L., Sciutti, F., Belliato, M., Iotti, G. A., ... & Bressan, M. A. (2020). Mild head trauma in elderly patients: Experience of an emergency department. Heliyon, 6(7), e04226.

Risikofaktor	Definition	Statistisch signifikant in multivariater Regressionsanalyse assoziiert mit pathologischer cCT	Statistisch signifikant in univariater Regressionsanalyse assoziiert mit pathologischer cCT	Nicht statistisch signifikant	CCHR, NEXUS II, NOC, NICE
1-year increase in age	-	OR 1023/year; **	Nicht berichtet		
≥ 75y.	-	OR 2.82; **	Nicht berichtet		NOC, NICE, NEXUS II, CCHR

Legende: * = p< 0.05; ** = p < 0.001; *** = p < 0.0001; NICE = NICE Head injury: assessment and early management; CCHR = Canadian CT-Head Rule; NEXUS II = National Emergency X-Radiography Utilization Study II; NOC = New Orleans Criteria

20) Cipriano, A., Pecori, A., Bionda, A. E., Bardini, M., Frassi, F., Leoli, F., ... & Santini, M. (2018). Intracranial hemorrhage in anticoagulated patients with mild traumatic brain injury: significant differences between direct oral anticoagulants and vitamin K antagonists. Internal and Emergency Medicine, 13, 1077-1087.

Risikofaktor	Definition	Statistisch signifikant in multivariater Regressionsanalyse (*penalized approach*) assoziiert mit pathologischer cCT	Statistisch signifikant in univariater Regressionsanalyse assoziiert mit pathologischer cCT	Nicht statistisch signifikant	CCHR, NEXUS II, NOC, NICE
Age, year	-			x	
Male sex	-			x	
VKAs treatment	-	OR 3.364	OR 3.772 (95% CI 1.234 – 11.526); *		NICE
High-energy impact	-	OR 2.488	OR 4.615 (95% CI 1.554 – 13.704); *		NICE, CCHR
Trauma above the clavicles	-	OR 3.175	OR 4.424 (95% CI 1.269 – 15.427); *		NOC
LOC	-			x	NEXUS II, NICE
PTA	-	OR 2.570	OR 3.111 (95% CI 1.012 – 9.562), *		NICE, NOC
Presence of fractures	-	OR 2.569	OR 3.345 (95% CI 1.381 – 8.103), *		NEXUS II, NICE, CCHR
Concomitant antiplatelet treatment	-			x	

Low platelet count	< 150000/mm³	-	-	x	NEXUS II, NICE

Legende: * = p< 0.05; ** = p < 0.001; *** = p < 0.0001; cCT = cranial computed tomography; OR = Odd's Ratio; CI = confidence interval; VKA = Vitamin-K-Antagonist; LOC = loss of consciousness; PTA = posttraumatic amnesia; NICE = NICE Head injury: assessment and early management; CCHR = Canadian CT-Head Rule; NEXUS II = National Emergency X-Radiography Utilization Study II; NOC = New Orleans Criteria

21) Reddy, S., Sharma, R., Grotts, J., Ferrigno, L., & Kaminski, S. (2014). Incidence of intracranial hemorrhage and outcomes after ground-level falls in geriatric trauma patients taking preinjury anticoagulants and antiplatelet agents. The American surgeon, 80(10), 975-978.

Risikofaktor	Definition	Statistisch signifikant in multivariater Regressionsanalyse assoziiert mit pathologischer cCT	Statistisch signifikant in univariater Regressionsanalyse assoziiert mit pathologischer cCT	Nicht statistisch signifikant	CCHR, NEXUS II, NOC, NICE
GCS < 15	-	OR 2.29 (95% CI 1.32 – 3.98); *	Nicht berichtet		NEXUS II, NICE, CCHR
Loss of consciousness	-	OR 3.14 (95% CI 1.81 – 5.46); **	Nicht berichtet		NICE
Hypertension	-	OR 1.91 (95% CI 1.06 – 3.42); *	Nicht berichtet		

Legende: * = p< 0.05; ** = p < 0.001; *** = p < 0.0001; cCT = cranial computed tomography; GCS = Glasgow Coma Scale; OR = Odd's Ratio; CI = confidence interval; NICE = NICE Head injury: assessment and early management; CCHR = Canadian CT-Head Rule; NEXUS II = National Emergency X-Radiography Utilization Study II; NOC = New Orleans Criteria

22) Nishijima, D. K., Offerman, S. R., Ballard, D. W., Vinson, D. R., Chettipally, U. K., Rauchwerger, A. S., ... & Holmes, J. F. (2012). Immediate and delayed traumatic intracranial hemorrhage in patients with head trauma and preinjury warfarin or clopidogrel use. Annals of emergency medicine, 59(6), 460-468.

Keine Regressionsanalyse durchgeführt

23) Cipriano, A., Park, N., Pecori, A., Bionda, A., Bardini, M., Frassi, F., ... & Ghiadoni, L. (2021). Predictors of post-traumatic complication of mild brain injury in anticoagulated patients: DOACs are safer than VKAs. Internal and Emergency Medicine, 16, 1061-1070.

Risikofaktor	Definition	Statistisch signifikant in multivariater Regressionsanalyse assoziiert mit pathologischer cCT	Statistisch signifikant in univariater Regressionsanalyse assoziiert mit pathologischer cCT	Nicht statistisch signifikant	CCHR, NEXUS II, NOC, NICE

Post-traumatic amnesia	-	*	Nicht berichtet		CCHR, NICE, NOC
Trauma above the clavicles	-	*	Nicht berichtet		NOC
High blood glucose	-	*	Nicht berichtet		
High blood pressure at arrival	-	*	Nicht berichtet		
Low prothrombin activity	-	*	Nicht berichtet		NEXUS II, NICE

Legende: * = p< 0.05; ** = p < 0.001; *** = p < 0.0001; cCT = cranial computed tomography; NICE = NICE Head injury: assessment and early management; CCHR = Canadian CT-Head Rule; NEXUS II = National Emergency X-Radiography Utilization Study II; NOC = New Orleans Criteria

24) Galliazzo, S., Bianchi, M. D., Virano, A., Trucchi, A., Donadini, M. P., Dentali, F., ... & Ageno, W. (2019). Intracranial bleeding risk after minor traumatic brain injury in patients on antithrombotic drugs. Thrombosis Research, 174, 113-120.

Risikofaktor	Definition	Statistisch signifikant in multivariater Regressionsanalyse assoziiert mit pathologischer cCT	Statistisch signifikant in univariater Regressionsanalyse assoziiert mit pathologischer cCT	Nicht statistisch signifikant	CCHR, NEXUS II, NOC, NICE
Antiplatelet medication	-		OR 1.88 (95% CI 1.09 – 3.24)		
VKA	-			x	NICE
DOAC	-			x	
Double therapy	-			x	
Age ≥ 65 years	-		OR 2.04 (95% CI 1.19 – 3.50)		NOC, NEXUS II, NICE, CCHR
Sex	-			x	
GCS < 15	-	OR 14.1 (95% CI 6.59 – 30.19)	OR 7.95 (95% CI 3.12 – 20.28)		NEXUS II, NICE, CCHR
LOC	-			x	NICE
Amnesia	-	OR 6.49 (95% CI 3.57 – 11.82)	OR 8.41 (95% CI 4.96 – 14.26)		NICE, CCHR, NOC

Neurological signs	-			x	NICE, NEXUS II
Seizure	-			x	NOC
Headache	-			x	NOC
Vomiting	-	OR 4.45 (95% CI 1.47 – 13.50)	OR 8.76 (95% CI 3.62 – 21.19)		NICE, CCHR, NOC, NEXUS II
Clinical signs of cranial fracture	-	OR 8.41 (95% CI 2.12 – 33.33)	OR 20.1 (95% CI 6.20 – 65.0)		NEXUS II, NICE, CCHR
Complicated contused lacerated wound	-			x	
Critical dynamic	-		OR 5.45 (95% CI 2.20 – 13.55)		
History of epilepsy	-			x	
Previous stroke/TIA/neurosurgery	-			x	
Drug/alcohol intoxication	-			x	NOC
History of cerebral neoplasia	-			x	
Scalp lesions	-	OR 2.31 (95% CI 1.09 – 4.89)	OR 3.60 (95% CI 1.77 – 7.30)		NOC, NEXUS II

Legende: * = p< 0.05; ** = p < 0.001; *** = p < 0.0001; cCT = cranial computed tomography; VKA = Vitamin-K-Antagonist; DOAC = neue orale Antikoagulantien; GCS = Glasgow Coma Scale; LOC = loss of consciousness; TIA = transitorische ischämische Attacke; OR = Odd's Ratio; CI = confidence interval; NICE = NICE Head injury: assessment and early management; CCHR = Canadian CT-Head Rule; NEXUS II = National Emergency X-Radiography Utilization Study II; NOC = New Orleans Criteria

25) Sauter, T. C., Ziegenhorn, S., Ahmad, S. S., Hautz, W. E., Ricklin, M. E., Leichtle, A. B., ... & Exadaktylos, A. K. (2016). Age is not associated with intracranial haemorrhage in patients with mild traumatic brain injury and oral anticoagulation. Journal of negative results in biomedicine, 15(1), 1-5.

Risikofaktor	Definition	Statistisch signifikant in multivariater Regressionsanalyse assoziiert mit pathologischer cCT	Statistisch signifikant in univariater Regressionsanalyse assoziiert mit pathologischer cCT	Nicht statistisch signifikant	CCHR, NEXUS II, NOC, NICE
Age	-	Nicht berichtet			

GCS	-	Nicht berichtet	OR 0.419 (CI 0.258 – 0.680); **		NEXUS II, NICE, CCHR
Creatinine	-	Nicht berichtet			
INR	-	Nicht berichtet			
Gender	-	Nicht berichtet			
Atrial fibrilliation	-	Nicht berichtet			
Thromboembolic event	-	Nicht berichtet	OR 0.486 (95% CI 0.257 – 0.918); *		
Mechanical heart valve	-	Nicht berichtet			
ASS	-	Nicht berichtet			
Clopidogrel	-	Nicht berichtet			
Road traffic accident	-	Nicht berichtet			NICE, CCHR
Fall	-	Nicht berichtet			
Direct trauma	-	Nicht berichtet			

Legende: * = p< 0.05; ** = p < 0.001; *** = p < 0.0001; cCT = cranial computed tomography; GCS = Glasgow Coma Scale; INR = international normalized ratio; ASS = Aspirin; OR = Odd's Ratio; CI = confidence interval; NICE = NICE Head injury: assessment and early management; CCHR = Canadian CT-Head Rule; NEXUS II = National Emergency X-Radiography Utilization Study II; NOC = New Orleans Criteria

26) Moore, M. M., Pasquale, M. D., & Badellino, M. (2012). Impact of age and anticoagulation: need for neurosurgical intervention in trauma patients with mild traumatic brain injury. Journal of Trauma and Acute Care Surgery, 73(1), 126-130.

Nicht über Regressionsanalyse berichtet

27) Mason, S., Kuczawski, M., Teare, M. D., Stevenson, M., Goodacre, S., Ramlakhan, S., ... & Rothwell, J. (2017). AHEAD study: an observational study of the management of anticoagulated patients who suffer head injury. BMJ open, 7(1), e014324.

Risikofaktor	Definition	Statistisch signifikant in multivariater Regressionsanalyse assoziiert mit pathologischer cCT	Statistisch signifikant in univariater Regressionsanalyse assoziiert mit pathologischer cCT	Nicht statistisch signifikant	CCHR, NEXUS II, NOC, NICE
GCS < 15	-	Nicht berichtet	RR 4.82 (95% CI 3.66 – 6.35); **		NICE, NEXUS II, CCHR
Vomiting	-	- (in Pat. mit GCS = 15)	RR 3.94 (95% CI 2.32 – 6.70); **		NOC, NICE, NEXUS II, CCHR

Amnesia	-	RR 3.48 (95% CI 2.13 – 5.70); p< 0.001 (in Pat. mit GCS = 15)	RR 4.37 (95% CI 3.05 – 6.25); **		NICE, CCHR, NOC
Headache	-	- (in Pat. mit GCS = 15)	RR 2.11 (95% CI 1.33 – 3.34); **		NOC
LOC	-	- (in Pat. mit GCS = 15)	RR 4.14 (95% CI 2.92 – 5.88); **		NICE

Legende: * = p< 0.05; ** = p < 0.001; *** = p < 0.0001; cCT = cranial computed tomography; GCS = Glasgow Coma Scale, LOC = loss of consciousness; RR = relative risk; CI = confidence interval; Pat. = Patient:innen; NICE = NICE Head injury: assessment and early management; CCHR = Canadian CT-Head Rule; NEXUS II = National Emergency X-Radiography Utilization Study II; NOC = New Orleans Criteria

28) Turcato, G., Zannoni, M., Zaboli, A., Zorzi, E., Ricci, G., Pfeifer, N., ... & Bonora, A. (2019). Direct oral anticoagulant treatment and mild traumatic brain injury: risk of early and delayed bleeding and the severity of injuries compared with vitamin K antagonists. The Journal of emergency medicine, 57(6), 817-824.

Risikofaktor	Definition	Statistisch signifikant in multivariater Regressionsanalyse assoziiert mit pathologischer cCT	Statistisch signifikant in univariater Regressionsanalyse assoziiert mit pathologischer cCT	Nicht statistisch signifikant	CCHR, NEXUS II, NOC, NICE
VKA treatment	-	OR 2.327 (95% CI 1.117 – 4.847); *	*		NICE
Previous neurosurgery	-			x	
High-energy impact	-	OR 11.229 (95% CI 3.265 – 38.617); **	**		NICE, CCHR
Alcohol abuse	-			x	NOC
Antiplatelet treatment	-			x	
Amnesia	-	OR 2.814 (95% CI 1.102 – 6.556); *	**		NOC, NICE, CCHR
LOC	-	OR 5.286 (95% CI 1.102 – 25.348); *	**		NICE
Posttraumatic seizures	-			x	NOC
Vomiting	-			x	NICE, NEXUS II, NOC, CCHR
GCS < 15	-	OR 2.742 (95% CI 1.297 – 5.797); *	**		NEXUS II, NICE, CCHR
Worsening headache	-			x	NOC

Trauma beyond clavicles	-		**		NOC
Presence of cranial fracture	-		**		NEXUS II, NICE, CCHR

Legende: * = p< 0.05; ** = p < 0.001; *** = p < 0.0001; cCT = cranial computed tomography; VKA = Vitamin-K-Antagonist; LOC = loss of consciousness; GCS = Glasgow Coma Scale; OR = Odd's Ratio; CI = confidence interval; NICE = NICE Head injury: assessment and early management; CCHR = Canadian CT-Head Rule; NEXUS II = National Emergency X-Radiography Utilization Study II; NOC = New Orleans Criteria

29) Turcato, G., Zaboli, A., Pfeifer, N., Maccagnani, A., Tenci, A., Giudiceandrea, A., ... & Brigo, F. (2021). Decision tree analysis to predict the risk of intracranial haemorrhage after mild traumatic brain injury in patients taking DOACs. The American Journal of Emergency Medicine, 50, 388-393.

Risikofaktor	Definition	Statistisch signifikant in multivariater Regressionsanalyse assoziiert mit pathologischer cCT	Statistisch signifikant in univariater Regressionsanalyse assoziiert mit pathologischer cCT	Nicht statistisch signifikant	CCHR, NEXUS II, NOC, NICE
Age	-	Nicht berichtet			
Major trauma dynamic	-	Nicht berichtet	**		NICE, CCHR
Antiplatelet therapy	-	Nicht berichtet			
Previous neurosurgery	-	Nicht berichtet	*		
Dementia or severe disabling psychiatric disease	-	Nicht berichtet			
Acute intoxication	-	Nicht berichtet			NOC
Post-traumatic transitory loss of consciousness	-	Nicht berichtet	**		NICE
GCS < 15	-	Nicht berichtet	**		NOC, NEXUS II, NICE, CCHR
Visible trauma above the clavicles	-	Nicht berichtet	**		NOC
Vomiting	-	Nicht berichtet	**		NOC, NEXUS

					II, NICE, CCHR
Post-traumatic headache	-	Nicht berichtet			NOC
Post-traumatic seizure		Nicht berichtet	*		NOC
Signs of possible fracture oft he skull base	-	Nicht berichtet	**		NOC, NEXUS II, CCHR
Sex	-	Nicht berichtet			
HAS-BLED ≥ 3	-	Nicht berichtet	*		
Systolic BP	-	Nicht berichtet	*		
Diastolic BP	-	Nicht berichtet			
HR	-	Nicht berichtet			
Oxygen saturation	-	Nicht berichtet			
Haemoglobin	-	Nicht berichtet			
Platelets	-	Nicht berichtet			
Blood sugar	-	Nicht berichtet			
Creatinine	-	Nicht berichtet			
Prothrombin time	-	Nicht berichtet			
Activated partial thromboplastin time	-	Nicht berichtet			

Legende: * = p< 0.05; ** = p < 0.001; *** = p < 0.0001; cCT = cranial computed tomography; GCS = Glasgow Coma Scale; HAS-BLED = HAS-BLED-Score; BP = blood pressure; HR = heart rate; NICE = NICE Head injury: assessment and early management; CCHR = Canadian CT-Head Rule; NEXUS II = National Emergency X-Radiography Utilization Study II; NOC = New Orleans Criteria
