# Supplementary material for: Diagnostic algorithm for the management of patients ≥ 65 years with a mild traumatic brain injury in the emergency department
Source: Unfallchirurgie (Heidelb). 2026 Aug 3;129(10):696–708. [Article in German] doi: 10.1007/s00113-026-01737-4 (PMC13601116; doi:10.1007/s00113-026-01737-4)
Supplement: Supplementary file 5 [file 113_2026_1737_MOESM5_ESM.pdf]

Appendix 5 - MINORS

F1

TABELLE 9: MINORS F2

**MINORS-*Item***	**Alrajhi 2015**	**Brewer 2011**	**Brown 2011**	**Cipriano 2018**	**Cipriano 2021**	**Colas 2021**	**Fabbri 2010**	**Fournier 2019**	**Galliazzo 2019**	**Hamden 2014**	**Ibañez 2018**	**Lee 2009**	**Leitner 2021**	**Mack 2003**	**Mason 2017**	**McCammack 2015**	**Moore 2012**	**Mori 2021**	**Nishijima 2012**	**Nishijima 2013**	**O'Brien 2020**	**Pages 2020**	**Reddy 2014**	**Riccardi 2013**	**Sauter 2016**	**Savioli 2020**	**Teeratakulpisarn 2021**	**Turcato 2019**	**Turcato 2021**	**Yuksen 2018**
a)	2	2	2	2	2	2	2	2	2	2	2	2	2	2	2	2	2	2	2	2	2	2	2	2	2	2	2	2	2	2
b)	2	2	2	2	2	2	2	2	2	2	2	2	2	2	2	2	2	2	2	2	2	2	2	2	2	2	2	2	2	2
c)	2	2	2	2	2	2	2	2	2	2	2	2	2	2	2	2	2	2	2	2	2	2	2	2	2	2	2	2	2	2
d)	2	2	2	2	2	2	2	2	2	2	2	2	2	2	2	2	2	2	2	2	2	2	1	2	2	2	2	2	2	2
e)	0	0	0	2	2	2	0	1	0	0	0	0	0	0	0	2	0	2	1	1	0	0	0	0	0	0	0	0	0	0
f)	1	2	2	2	2	2	2	2	2	2	2	2	2	2	2	2	2	2	2	2	2	2	2	2	2	2	2	2	2	2
g)	2	2	2	2	2	2	2	2	2	2	2	2	2	2	2	2	2	2	2	2	2	2	2	2	2	2	2	2	2	2
h)	2	0	0	0	2	2	0	2	0	2	2	2	0	0	2	0	0	2	0	0	0	0	0	0	0	0	0	0	0	0
i)	-	-	-	2	2	-	-	-	2	2	-	-	2	2	-	2	2	2	2	-	-	2	2	2	2	2	2	2	2	2
j)	-	-	-	2	2	-	-	-	2	2	-	-	2	2	-	2	2	1	2	-	-	2	2	2	2	2	2	2	2	2
k)	-	-	-	2	2	-	-	-	2	2	-	-	2	2	-	2	2	0	2	-	-	2	2	2	2	2	2	2	2	2
l)	-	-	-	2	2	-	-	-	2	2	-	-	2	2	-	2	2	2	2	-	-	2	2	2	2	2	2	2	2	2

**GP**	13/16	12/16	12/16	22/24	24/24	16/16	12/16	15/16	20/24	22/24	14/16	14/16	20/24	20/24	14/16	22/24	20/24	21/24	21/24	13/16	12/16	20/24	19/24	20/24	20/24	20/24	20/24	20/24	20/24	20/24

Tabelle 9, Legende: 2 Punkte = berichtet und adäquat; 1 Punkt = berichtet, aber inadäquat; 0 Punkte = nicht berichtet; GP = Gesamtpunktzahl/Maximalpunktzahl

Tabelle 13: F3 + F4 MINORS

MINORS-Item	Bauman 2017 (9)	Campiglio 2017 (94)	Cipriano 2018 (8)	Cohan 2020 (95)	Covino 2021 (13)	Huang 2019 (96)	Kaen 2010 (10)	Leitner 2021 (23)	Lim 2016 (11)	Macedo 2017 (97)	Marques 2021 (98)	McCammack 2015 (5	Menditto 2012 (99)	Mourad 2021 (100)	Scantling 2017 (12)	Schoonman 2014	Singleton 2021 (101)	Tauber 2009 (102)	Turcato 2022 (103)	Valiuddin 2021 (104)	Yun 2018 (106)
a)	2	2	2	2	2	2	2	2	2	2	2	2	2	2	2	2	2	2	2	2	2
b)	2	2	2	2	2	1	2	2	2	2	2	2	2	2	2	2	2	2	2	2	0
c)	2	2	2	2	2	2	2	2	2	2	2	2	2	2	2	2	2	2	2	2	2
d)	2	2	2	2	2	1	2	2	2	1	2	2	2	2	1	2	1	1	2	2	2
e)	2	0	2	0	0	0	0	0	0	0	0	2	0	0	0	0	0	2	0	2	2
f)	2	2	2	2	2	2	2	2	2	2	2	2	2	2	2	2	2	2	2	1	2
g)	0	2	2	0	2	2	2	2	2	2	0	2	2	0	2	2	2	2	0	0	0
h)	0	2	0	0	0	0	0	0	2	0	0	0	0	0	2	0	0	0	0	0	0
i)	-	-	2	2	2	-	-	2	-	-	-	2	-	2	-	-	2	-	-	2	2
j)	-	-	2	2	2	-	-	2	-	-	-	2	-	2	-	-	2	-	-	2	1
k)	-	-	2	2	2	-	-	2	-	-	-	2	-	2	-	-	2	-	-	2	2
l)	-	-	2	2	2	-	-	2	-	-	-	2	-	1	-	-	2	-	-	1	2
GP	12/16	14/16	22/24	18/24	20/24	10/16	12/16	20/24	14/16	11/16	10/16	22/24	12/16	17/24	13/16	12/16	19/24	13/16	10/16	18/24	17/24

Tabelle 13, Legende: 2 Punkte = berichtet und adäquat; 1 Punkt = berichtet, aber inadäquat; 0 Punkte = nicht berichtet; GP = Gesamtpunktzahl/Maximalpunktzahl
