# Supplementary material for: Diagnostic algorithm for the management of patients ≥ 65 years with a mild traumatic brain injury in the emergency department
Source: Unfallchirurgie (Heidelb). 2026 Aug 3;129(10):696–708. [Article in German] doi: 10.1007/s00113-026-01737-4 (PMC13601116; doi:10.1007/s00113-026-01737-4)
Supplement: Supplementary file 6 [file 113_2026_1737_MOESM6_ESM.pdf]

Appendix 6- Vote counting based on direction of effect

F1

Referenz	Studiendesign	Anzahl Patient:innen	1) Empfehlung für eine initiale cCT bei vorhandenen Einflussfaktoren	2) Empfehlung für eine initiale cCT für alle Patient:innen, unabhängig von individuellen Einflussfaktoren
Fabbri 2010	P-U-KS	14.228	▲	▼
Mason 2017	R-M-KS	3.534	-	-
Savioli 2020	R-U-KS	2.851	-	-
Riccardi 2013	R-U-KS	2.149	▲	-
Galliazzo 2019	R-U-KS	1.846	-	-
Leitner 2021	R-U-KS	1.788	-	-
Turcato 2021	R-M-FS	1.146	-	-
Mori 2021	R-U-FKS	1.118	▲	-
Nishijima 2012	P-M-KS	1.064	-	▲
Nishijima 2013	P-M-QS	982	▼	▲
Lee 2009	R-U-KS	898	-	▲
Colas 2021	P-U-QS	840	-	-
Hamden 2014	P-U-KS	799	-	-
Yuksen 2018	R-U-KS	708	-	-
Reddy 2014	R-U-KS	562	-	-
Ibañez 2018	P-U-KS	504	-	-
Pages 2020	R-U-KS	500	▲	▼
Cipriano 2021	P-U-KS	473	-	-
Turcato 2019	R-U-KS	451	-	-
O'Brien 2020	R-M-KS	311	-	▲
Sauter 2016	R-U-QS	260	▼	▲
Cipriano 2018	P-U-KS	206	-	-
Alrajhi 2015	R-M-KS	176	-	-
McCammack 2015	R-U-KS	144	▲	-
Brewer 2011	R-U-QS	141	▼	▲
Mack 2003	R-U-KS	133	▼	▲
Fournier 2019	R-U-KS	104	▲	▼
Moore 2012	R-U-QS	101	-	▲
Teeratakulpisarn 2021	R-U-KS	100	-	-
Brown 2011	P-U-FS	66	▲	-

Tabelle 11, Legende:
Maßnahme empfohlen: ▲
Maßnahme nicht empfohlen: ▼
Maßnahme nicht untersucht: -
Code Studiendesign: R = retrospektiv, P = prospektiv, U = unizentrisch, M = multizentrisch, KS = Kohortenstudie
FS = Fallserie, QS = Querschnittsstudie, FKS = Fall-Kontroll-Studie
Farbkodierung: suffiziente Berichterstattung (MINORS 16-13 bzw. 24-19 von maximal 16 bzw. 24 Punkten)
moderate Berichterstattung (MINORS 12-9 bzw. 19-15 von maximal 16 bzw. 24 Punkten)
insuffiziente Berichterstattung (MINORS ≤ 8 bzw. 14 von maximal 16 bzw. 24 Punkten)

F2 + F3

Studie	Studiendesign	Anzahl Patietnt:innen	1) Routinemäßige Kontroll-cCT	2) Überwachung ohne routinemäßige Kontroll-cCT	3) Entlassung ohne Überwachung und routinemäßiger Kontroll-cCT
Leitner 2021	R-U-KS	1.788	▲	-	-
Bauman 2017	P-U-FS	1.501	▼	▲	(▲)
Turcato 2022	R-M-KS	1.426	▼	-	-
Covino 2021	R-U-QS	685	▼	(▲)	-
Mourad 2021	R-U-KS	498	▼	-	-
Cohan 2020	R-U-KS	332	(▼)	-	-
Lim 2016	R-U-KS	298	-	▲	-
Valiuddin 2021	R-U-KS	287	(▼)	-	(▲)
Scantling 2017	R-U-QS	234	▼	▲	-
Schoonman 2014	R-U-FS	211	-	▼	-
Cipriano 2018	P-U-KS	206	▼	▲	-
Huang 2019	R-U-QS	204	▼	-	-
Marques 2021	R-U-FS	201	▼	-	(▲)
Kaen 2010	P-U-QS	137	▼	▲	-
Tauber 2009	P-U-QS	100	▲	(▲)	-
Menditto 2012	P-U-FS	87	▲	-	-

Tabelle 16, Legende: cCT = kranielle Computertomographie;
Maßnahme empfohlen: ▲
Maßnahme nicht empfohlen: ▼
Maßnahme nicht untersucht: -
() = Maßnahme laut Studie möglich, aber nicht explizit empfohlen
Code Studiendesign: R = retrospektiv, P = prospektiv, U = unizentrisch, M = multizentrisch, KS = Kohortenstudie
FS = Fallserie, QS = Querschnittsstudie, FKS = Fall-Kontroll-Studie
Farbkodierung: suffiziente Berichterstattung (MINORS 16-13 bzw. 24-19 von maximal 16 bzw. 24 Punkten)
moderate Berichterstattung (MINORS 12-9 bzw. 19-15 von maximal 16 bzw. 24 Punkten)
insuffiziente Berichterstattung (MINORS ≤ 8 bzw. 14 von maximal 16 bzw. 24 Punkten)

Studie	Studiendesign	Anzahl Patient:innen	1) Routinemäßige Kontroll-cCT	2) Überwachung ohne routinemäßige Kontroll-cCT	3) Entlassung ohne Überwachung und routinemäßiger Kontroll-cCT
Leitner 2021	R-U-KS	1.788	▲	-	-
Yun 2018	R-U-QS	296	-	-	-
Singleton 2021	P-U-KS	138	-	▲	(▲)

Tabelle 17, Legende: cCT = kranielle Computertomographie;
Maßnahme empfohlen: ▲
Maßnahme nicht empfohlen: ▼
Maßnahme nicht untersucht: -
Code Studiendesign: R = retrospektiv, P = prospektiv, U = unizentrisch, M = multizentrisch, KS = Kohortenstudie FS = Fallserie, QS = Querschnittsstudie, FKS = Fall-Kontroll-Studie
Farbkodierung: suffiziente Berichterstattung (MINORS 16-13 bzw. 24-19 von maximal 16 bzw. 24 Punkten)
moderate Berichterstattung (MINORS 12-9 bzw. 19-15 von maximal 16 bzw. 24 Punkten)
insuffiziente Berichterstattung (MINORS ≤ 8 bzw. 14 von maximal 16 bzw. 24 Punkten)
