# Supplementary material for: Diagnostic algorithm for the management of patients ≥ 65 years with a mild traumatic brain injury in the emergency department
Source: Unfallchirurgie (Heidelb). 2026 Aug 3;129(10):696–708. [Article in German] doi: 10.1007/s00113-026-01737-4 (PMC13601116; doi:10.1007/s00113-026-01737-4)
Supplement: Supplementary file 7 [file 113_2026_1737_MOESM7_ESM.pdf]

Appendix 7- Sonstige Publikationen

Im Folgenden werden vier Studien vorgestellt, die die Einschlusskriterien der systematischen Literatursuche erfüllen, deren Ergebnisse aber nicht den Forschungsfragen F2 – F4 zuordbar sind. Die Ergebnisse sind demnach nicht in den Algorithmus eingeflossen.

1) Cheng et al. (2014) führten eine prospektive multizentrische Kohortenstudie durch mit dem Ziel, die Korrelation zwischen leichten SHT und der Mortalität älterer Patient:innen ≥ 65 Jahre zu untersuchen. Anhand von Daten des taiwanischen *National Health Insurance Programs* wurde das Mortalitätsrisiko zwischen älteren Patient:innen mit leichtem SHT (n = 5.997) und einer Kontrollgruppe (n = 84.117) über einen Fünf-Jahres-Zeitraum hinweg untersucht. In der untersuchten Population waren die Patient:innen mit leichtem SHT signifikant älter und zeigten signifikant mehr Erkrankungen an arterieller Hypertension, Diabetes, einem ischämischen Schlaganfall und Demenz als die Kontrollgruppe. Die SHT-Patient:innen hatten auch signifikant häufiger eine Alkoholintoxikation, intrakranielle Blutungen und einen höheren *Charlson Comorbidity Index* als die Patient:innen in der Kontrollgruppe. In der multivariaten Cox-Regression hatten Patient:innen mit leichtem SHT eine höhere *Hazard Ratio* als die Kontrollgruppe, auch nach Bereinigung der Kovariaten: Alter, Geschlecht, Urbanisationsgrad, sozioökonomischen Status, Diabetes, Hypertension, Alkoholintoxikation, Zustand nach ischämischem Apoplex, Zustand nach intrakranieller Hämorrhagie, malignen Erkrankungen, Demenz und *Charlson Comorbidity Index*. Die Autor:innen schlussfolgern, dass ein leichtes SHT ein signifikanter unabhängiger Risikofaktor für die Mortalität in älteren Patient:innen ist.

2) In einer weiteren Studie beschäftigten sich Goldschlager et al. mit Patient:innen, die nach klinischer Verschlechterung bei initial leichtem SHT verstarben. In einem 10-Jahres-Zeitraum konnten 15 Patient:innen mit einem eingängigen GCS von 13 – 15 identifiziert werden, die an den Folgen des SHT verstarben. Dies entsprach 2,3 % aller Patient:innen, die im Untersuchungszeitraum aufgrund eines SHT verstarben. Die betroffenen Patient:innen waren älter (53 % älter als 70 Jahre, n = 8), mit einem Sturz als Unfallmechanismus (60 %, n = 9) und entwickelten häufiger ein akutes Subduralhämatom (47 %, n = 7). Die Autor:innen berichteten von individuellen Behandlungsumständen, die ihrer Meinung nach den negativen Verlauf der Patient:innen begünstigten. Dazu zählten Verzögerung in der Durchführung der CT-Bildgebung, Mangel an Kommunikation und Verlegung zur neurochirurgischen Abteilung, verfrühte Entlassung und das nicht rechtzeitige Korrigieren von Thrombozytopenien und Koagulopathien.

3) Moyer et al. (2018) untersuchten die Notwendigkeit der *Trauma Team Activation* (TTA), vergleichbar mit dem Ausrufen eines Schockraums in Deutschland, bei geriatrischen Patient:innen mit intrakranieller Blutung und unter Einnahme von Antikoagulanzien bzw. Thrombozytenaggregationshemmern. Eingeschlossen wurden Patient:innen mit bildgebend bestätigter intrakranieller Blutung nach Sturz, ≥ 65 Jahre alt mit einem GCS ≥ 13 und einem *head-Abbreviated Injury Score* (AIS) - Kopf ≥ 2. Primärer Endpunkt waren Mortalität im stationären Aufenthalt bzw. Umstellung auf palliative Versorgung, sekundärer Endpunkt die Notwendigkeit neurochirurgischer Intervention. Patient:innen unter TTA erhielten im Vergleich schneller eine cCT: im Mittelwert 20 Minuten (IQR 13 – 33 Minuten) mit TTA als ohne TTA (Mittelwert 80 Minuten (IQR 56 – 131 Minuten; $p < 0.0001$)). Auch die Aufhebung der medikamentösen Antikoagulation (durch Gabe von *Fresh Frozen Plasma* oder Prothrombinkomplex-Konzentrat) erfolgte schneller unter TTA: Mittelwert 105 Minuten (IQR 67 – 161 Minuten) versus Mittelwert 255 Minuten (IQR 198 – 340 Minuten) ohne TTA ($p < 0.0001$). Es konnten jedoch keine signifikanten Unterschiede in Bezug auf primäre und sekundäre Endpunkte (Mortalität, neurochirurgische Intervention) bei Patient:innen mit TTA versus ohne TTA beobachtet werden.

4) Techar et al. (2019) verfolgten mit ihrer Studie das Ziel, das optimale Zeitfenster für die initiale cCT bei Patient:innen mit leichtem SHT (GCS > 13) und ≥ 50 Jahren zu untersuchen. Des Weiteren sollten die Folgen von Verzögerungen in der Bildgebung auf die klinischen Ergebnisse untersucht werden. Es wurde berichtet, dass die Mortalität am geringsten bei 35 Minuten bis zur Durchführung der cCT war. Jede einminütige Verzögerung war assoziiert mit einer Mortalitätssteigerung von 2 % (OR 1.02, 95 % KI 1.01 – 1.03). Im Vergleich zwischen Patient:innen mit Bildgebung innerhalb von 35 Minuten versus nach 35 Minuten konnten keine signifikanten Unterschiede im Auftreten größerer Komplikationen (z. B. Sepsis, pulmonale Embolie, Pneumonie, akutes Nierenversagen u.a.) festgestellt werden (OR 0.83, 95 % KI 0.60 – 1.17). Die Gruppe der Patient:innen mit früher Bildgebung (< 35 Minuten) hatte jedoch eine verminderte Sterblichkeit (1,1 % versus 1,9 %, p = 0.01) und erhielten wahrscheinlicher innerhalb von vier Stunden *Fresh Frozen Plasma* bei vorbestehender medikamentöser Antikoagulation (33.2 % versus 25.3 %, p = 0.02). Die Autor:innen führen die erhöhte Sterblichkeit in der Gruppe der Patient:innen mit Bildgebung nach 35 Minuten auf potenziell verzögerte therapeutische Interventionen zurück. Sie empfehlen, die initiale cCT bei älteren Patient:innen mit leichtem SHT innerhalb der ersten 35 Minuten nach Aufnahme in der Notaufnahme durchzuführen.
