# Supplementary material for: Diagnostic algorithm for the management of patients ≥ 65 years with a mild traumatic brain injury in the emergency department
Source: Unfallchirurgie (Heidelb). 2026 Aug 3;129(10):696–708. [Article in German] doi: 10.1007/s00113-026-01737-4 (PMC13601116; doi:10.1007/s00113-026-01737-4)
Supplement: Supplementary file 8 [file 113_2026_1737_MOESM8_ESM.pdf]

Appendix 8- Multivariate Regressionsanalysen Primärstudien

Studie	Aufgetretenes neurologisches Defizit	GCS ≤ 14[x]	Mehr als einmaliges Erbrechen[x]	Aufgetretene (auch kurze) Bewusstlosigkeit [x]	Aufgetretene Amnesie[x]	Medikamentöse Antikoagulation[x]	Bekannte Blutgerinnungsstörung	Alter ≥ 65 Jahre	Alter ≥ 75 Jahre	Klinische Anzeichen auf Schädel(basis)fraktur[x]	Krampfanfall	Kopfschmerzen	Alkohol- bzw. Drogenintoxikation [x]	Männliches Geschlecht	Posttraumatische Hirnschäden in der Vorgeschichte	Hochrasanztrauma[x]	Arterielle Hypertonie	Veränderter Bewusstseinszustand	Hoher Blutzuckerwert[x]
Cipriano 2018	-	-	-	▼	▲ *	▲ *	▼	-	-	▲ *	-	-	-	▼	-	▲ *	-	-	-
Cipriano 2021	-	-	-	-	*	-	*	-	-	-	-	-	-	-	-	-	*	-	*
Fabbri 2010	▲ *	▲ *	▲ *	▲ *	▼	▲ *	▲ *	-	▲	▲ *	▲ *	▼	▼	-	-	▲	-	-	-

Galliazzo 2019	▲	▲*	▲*	▲	▲*	▲	-	▲	-	▲*	▶	▼	▲	▲	-	-	-	-	-
Hamden 2014	-	-	-	▲*	-	▲	-	-	-	▲*	-	▲	-	-	▼	-	-	-	-
Ibañez 2018	-	▲*	▲	▲*	-	▲*	-	-	-	-	-	-	-	-	-	▲*	-	-	-
Lee 2009	▲	▲*	▲	▲	▲	-	▲	▲*	-	▲*	-	▲	▲	▲	-	-	-	-	-
Leitner 2021	-	-	-	-	-	▲*	-	-	-	▲*	-	-	-	-	-	-	-	-	-
Mack 2003	-			*			-	-	-						-	-	-		-
Mason 2017	-	-	▲	▲	▲*	-	-	-	-	-	-	▲	-	-	-	-	-	-	-
McCammack 2015	▲*		-	▲*	-	▲*	-	-	-	-	-	-	-	▼	-	-	-	-	-
Mori 2021	-	▲	▲*	▲	▲*	-	-	-		-	-			-	-	▲*		-	-
Nishijima 2013	-	-	▲*	▲	-	▲	-	-	-	-	-	▲	▲	-	-	-	-	▲*	-
Pages 2020	▲*	▲*	-	-	-			-	-	-	-	-	-	▲*	▲*	-	-	-	-
Reddy 2014	▲*	▲*	-	-	-	-	-	-	-	-	-	-	-	-	-	-	▲*	-	-
Savioli 2020	-	-	-	-	-	-	-	-	▲*	-	-	-	-	-	-	-	-	-	-
Teeratakulpisarn 2021	-	▲*	-	-	-	-	-	-	-	▲*	-	-	-	-	-	▲	-	-	-
Turcato 2019	-	▲*		▲*	▲*	▲*	-	-	-					-	-	▲*	-	-	-

Yuksen 2018	▲*	-	▲	▲*	▲	-	-	-	-	▲*	-	▲*	-	-	-	-	-	-	-
Evidenzstufe	SE	SE	SE	IE	IE	SE	IE	SE	ME	SE	IE	IE	IE	IE	IE	ME	LE	LE	-

Tabelle 10, Legende: GCS = *Glasgow Coma Scale*; ^{X} = Einflussfaktor mit unterschiedlichen Formulierungen/Definitionen in Primärstudien;

Risikofaktor (*Odd's Ratio* (OR) bzw. Relatives Risiko (RR) > 1): ▲ = N ≤ 100, ▲ = N ≥ 100, ▲ = N > 1000, ▲ = N > 10000;

Protektiver Faktor (OR bzw. RR < 1): ▼ = N ≤ 100, ▼ = N ≥ 100, ▼ = N > 1000; ▼ = N > 10000

Kein Effekt (OR bzw. RR = 1): ► = N ≤ 100, ► = N ≥ 100, ► = N > 1000; ► = N > 10000

* = statistisch signifikant

Freies Feld = untersucht, aber aufgrund von Berichterstattung nicht beurteilbar

Einflussfaktor nicht untersucht / keine multivariate Regressionsanalyse durchgeführt: -

Evidenzstufe: SE = Starke Evidenz, ME = Moderate Evidenz, LE = Limitierte Evidenz, IE = Inkonsistente Evidenz

Farbkodierung: suffiziente Berichterstattung (MINORS 16-13 bzw. 24-19 von maximal 16 bzw. 24 Punkten)

moderate Berichterstattung (MINORS 12-9 bzw. 19-15 von maximal 16 bzw. 24 Punkten)

insuffiziente Berichterstattung (MINORS ≤ 8 bzw. 14 von maximal 16 bzw. 24 Punkten)
